# Translating and evaluating historic phenotyping algorithms using SNOMED CT

**DOI:** 10.1093/jamia/ocac158

**Published:** 2022-09-09

**Authors:** Musaab Elkheder, Arturo Gonzalez-Izquierdo, Muhammad Qummer Ul Arfeen, Valerie Kuan, R Thomas Lumbers, Spiros Denaxas, Anoop D Shah

**Affiliations:** Institute of Health Informatics, University College London, London, UK; Institute of Health Informatics, University College London, London, UK; Health Data Research UK, London, UK; Institute of Health Informatics, University College London, London, UK; Institute of Health Informatics, University College London, London, UK; Institute of Health Informatics, University College London, London, UK; Barts Health NHS Trust, London, UK; University College London Hospitals NHS Trust, London, UK; Institute of Health Informatics, University College London, London, UK; Health Data Research UK, London, UK; British Heart Foundation Data Science Centre, London, UK; Institute of Health Informatics, University College London, London, UK; University College London Hospitals NHS Trust, London, UK

**Keywords:** terminology, phenotype, electronic health records, ontology, SNOMED CT

## Abstract

**Objective:**

Patient phenotype definitions based on terminologies are required for the computational use of electronic health records. Within UK primary care research databases, such definitions have typically been represented as flat lists of Read terms, but Systematized Nomenclature of Medicine—Clinical Terms (SNOMED CT) (a widely employed international reference terminology) enables the use of relationships between concepts, which could facilitate the phenotyping process. We implemented SNOMED CT-based phenotyping approaches and investigated their performance in the CPRD Aurum primary care database.

**Materials and Methods:**

We developed SNOMED CT phenotype definitions for 3 exemplar diseases: diabetes mellitus, asthma, and heart failure, using 3 methods: “primary” (primary concept and its descendants), “extended” (primary concept, descendants, and additional relations), and “value set” (based on text searches of term descriptions). We also derived SNOMED CT codelists in a semiautomated manner for 276 disease phenotypes used in a study of health across the lifecourse. Cohorts selected using each codelist were compared to “gold standard” manually curated Read codelists in a sample of 500 000 patients from CPRD Aurum.

**Results:**

SNOMED CT codelists selected a similar set of patients to Read, with F1 scores exceeding 0.93, and age and sex distributions were similar. The “value set” and “extended” codelists had slightly greater recall but lower precision than “primary” codelists. We were able to represent 257 of the 276 phenotypes by a single concept hierarchy, and for 135 phenotypes, the F1 score was greater than 0.9.

**Conclusions:**

SNOMED CT provides an efficient way to define disease phenotypes, resulting in similar patient populations to manually curated codelists.

## INTRODUCTION

Computational use of electronic health records (EHRs) for individual patient care (eg, clinical decision support, risk prediction models) or improving health services (eg, audit, service evaluation, research) requires patient phenotypes and outcomes to be defined based on data contained within the EHR.^1–3^ Many EHR systems record diagnoses using clinical terminologies, for example, the Clinical Modification of the International Classification of Diseases is used in the United States for coding encounter diagnoses for billing, and the Read Clinical Terminology (Read terms) has been used by GPs in the United Kingdom since 1985.^4^ Thus, definition of phenotypes often involves the creation of lists of clinical terms (often called “codelists,” “code sets,” or “value sets”). However, a single clinical meaning may be represented by more than 1 term, which may be in different parts of the terminology hierarchy. As a result, codelist generation can be an onerous task requiring the identification of all codes of potential relevance to a concept followed by manual adjudication.^5^

Systematized Nomenclature of Medicine—Clinical Terms (SNOMED CT) is a newer terminology^6^ which is becoming more commonly used in EHR data worldwide,^7^ and in the United States has been required for representing patient problems since 2013 in order for an EHR to receive government certification.^6^ The SNOMED CT ontology includes multiple hierarchies and concept attributes which may enable simpler, more efficient, and more reproducible phenotype definitions.^8^ However, these special features of SNOMED CT are currently under-used by researchers, particularly when using EHR data from UK primary care, which was historically coded using Read terms.

### SNOMED CT

SNOMED CT^6^ was initially developed in 1964 as the Systematized Nomenclature of Pathology,^9^ and merged with the Read Clinical Terminology to create SNOMED CT. It is now maintained by SNOMED International, formerly the International Health Terminology Standards Development Organisation^10^ and is mapped to multiple other terminologies.^11^ In the United Kingdom, SNOMED CT is replacing Read terms used for coding diagnoses in primary care.^4^

In SNOMED CT, alternative descriptions with the same meaning (eg, “Cardiac Insufficiency” and “Weak Heart”) are modeled as synonyms which belong to the same concept (“Heart failure [disorder]”), minimizing the need for extensive text searches. SNOMED CT contains a “relationship” table encoding attributes of concepts and links between concepts, such as parent–child “is a” relationships which link a concept to its subtypes. This makes it simple to define a codelist based on a small number of ancestor concepts. The polyhierarchical structure allows concepts to be linked to more than 1 ancestor, for example “Acute diastolic heart failure (disorder)” is a descendant of both “Acute heart failure (disorder)” and “Diastolic heart failure (disorder).” As well as parent–child relationships, SNOMED CT defines over 100 potential attributes for each concept,^12^ such as procedures associated with a body site, and enables new concepts to be defined by linking concepts using SNOMED CT expressions (also known as “post-coordination”).^13^ SNOMED CT has a mechanism for recording changes to concepts and their relationships over time.^14^ The NHS Digital SNOMED CT release includes a Query Table which allows inactive terms to be searched according to their original location in the SNOMED CT hierarchy.^15^

### Related work

The SNOMED CT “is a” relationships have been widely used to define groups of conditions for clinical and research purposes, particularly by the Observational Health Data Sciences and Informatics (OHDSI) community. OHDSI curate a set of vocabulary resources including SNOMED CT for the Observational Medical Outcomes Partnership (OMOP) Common Data Model. OMOP phenotypes can be defined using SNOMED CT hierarchies^16^ as has been used in the eMERGE network.^17^ SNOMED CT value sets used in Health Level 7 Fast Healthcare Interoperability Resources (HL7 FHIR) ValueSet resources for sending messages between EHR systems can also be defined using SNOMED CT expressions (“Content Logical Definition”) and expanded into a simple list of concepts.^18^

Chu et al compared the process of creation of hierarchy and list-based SNOMED CT value set creation for 10 conditions, and found that the hierarchy-based value sets were simpler (median 3 vs 78 concepts) and faster to construct.^8^ Willett et al found that 125 diagnosis value sets could be created using SNOMED CT hierarchies, requiring a median of only 2 concept hierarchies per value set, and were easy to understand and share.^19^ Winnenburg and Bodenreider derived metrics to quantify the completeness, correctness, and nonredundancy of published value sets compared to the hierarchy from the source terminology that has the same intended meaning.^20^

A number of software packages have been developed to assist in navigating SNOMED CT,^21^ interpreting SNOMED CT Expression Constraint Language^22^^,^^23^ and enabling fast evaluation of SNOMED CT expressions using graph-oriented databases.^24^

### Rationale and aim for the present study

EHR research databases from the UK primary care, such as the Clinical Practice Research Datalink,^25^^,^^26^ the Health Improvement Network,^27^ and UK Biobank,^28^ have been extensively used for research. Researchers using these databases have previously used Read term value sets to define phenotypes, as general practice data were previously recorded using Read terms. Hundreds of Read-based research phenotype definitions have been deposited in repositories such as ClinicalCodes.org,^29^ the CALIBER phenotype portal,^30^ and the new Health Data Research UK Phenotype Library.^31^

There is a lack of prior studies on the use of SNOMED CT with UK primary care research databases. We hypothesize that SNOMED CT can simplify the process of creating accurate and parsimonious definitions of disease diagnoses for use in Read or SNOMED CT-based EHR research databases.

In this study, we investigate the phenotyping process in detail for 3 exemplar diseases: diabetes mellitus, asthma, and heart failure, comparing patient cohorts derived using different phenotype definitions in the CPRD Aurum primary care database. Building on these findings and our previous semisupervised approach to phenotype definition,^28^ we also develop a semiautomated method for converting existing Read Version 2 phenotype definitions published by the CALIBER program^30^^,^^32^^,^^33^ to SNOMED CT. We apply this method to several hundred existing phenotype definitions to evaluate how well the method performs across a wide range of phenotypes. We also present a new package (Rdiagnosislist)^34^ for the R statistical system to make SNOMED CT convenient to use for researchers familiar with R.

## METHODS

### Overview

This study has 2 parts. First, we evaluated different methods of using SNOMED CT to create codelists for defining a small number of phenotypes, ranging from a simple hierarchy-based method to a more detailed, time-consuming but potentially more thorough method. Second, we chose a method that can be used semiautomatically at scale. We applied this method to the phenotyping task of identifying prevalent or historic medical conditions, which is commonly required for epidemiological studies using EHR. For both parts, we used as our reference Read V2 codelists which have been manually curated, validated, and previously used for EHR health research.

### Data source

We used a random sample of 500 000 patients from CPRD Aurum,^24^ a longitudinal database of routinely-collected EHR from UK primary care practices using the Egton Medical Information Systems EHR. CPRD Aurum was chosen because it contains data recorded using both Read and SNOMED CT.

The study period was from January 1, 2013 to December 31, 2018. We included patients aged over 18 years registered at practices contributing data of acceptable quality for research, and a minimum of 1 year of follow-up after the start date. In CPRD Aurum, all clinical concepts are denoted by a CPRD-specific identifier (“medcode”), and CPRD provides a dictionary linking each medcode with equivalent Read and SNOMED CT concepts. In order to provide a valid comparison between methods, we excluded CPRD data rows with observations that lacked Read V2 maps. We mapped all codelists to CPRD Aurum medcodes and excluded those that were not present in CPRD Aurum.

### Phenotype development using Read

We used Read version 2 codelists previously created by researchers for a range of EHR studies using the CALIBER data platform (based on the CPRD Gold primary care EHR database).^30^ These codelists had been created by keyword searches of term descriptions and by browsing the Read code hierarchy^32^ and were used in a study of 308 diseases across the lifecourse (“chronological map of human health”).^33^

### SNOMED CT terminology

We used the August 2020 SNOMED CT UK Edition (which includes the international release and the UK clinical and drug extensions), the UK Query Table and History Substitution Table August 2020 edition, UK Data Migration Workbench (Read to SNOMED mapping) April 2020 edition, and the CPRD Aurum medical dictionary. We obtained the SNOMED CT “Snapshot” source files from NHS Digital in Release Format 2. The SNOMED CT release is composed of 3 main tables: “concepts” which represents the clinical meanings, “descriptions” which contains the words or phrases used to describe each concept, and “relationships” which defines the connections between concepts. It also contains “reference sets” which are lists of SNOMED CT concepts for particular business uses, such as translation between SNOMED CT and other coding systems.

The software script to create the SNOMED CT phenotypes was developed in Python 3.8 using Spyder 4.1 IDE and the Networkx library version 2.5,^35^ which is a python package for network analysis. We also carried out analyses in R 4.1 using a new package that we developed, called “Rdiagnosislist.”^34^

### Diabetes mellitus, asthma, and heart failure phenotypes

**Figure 1.**
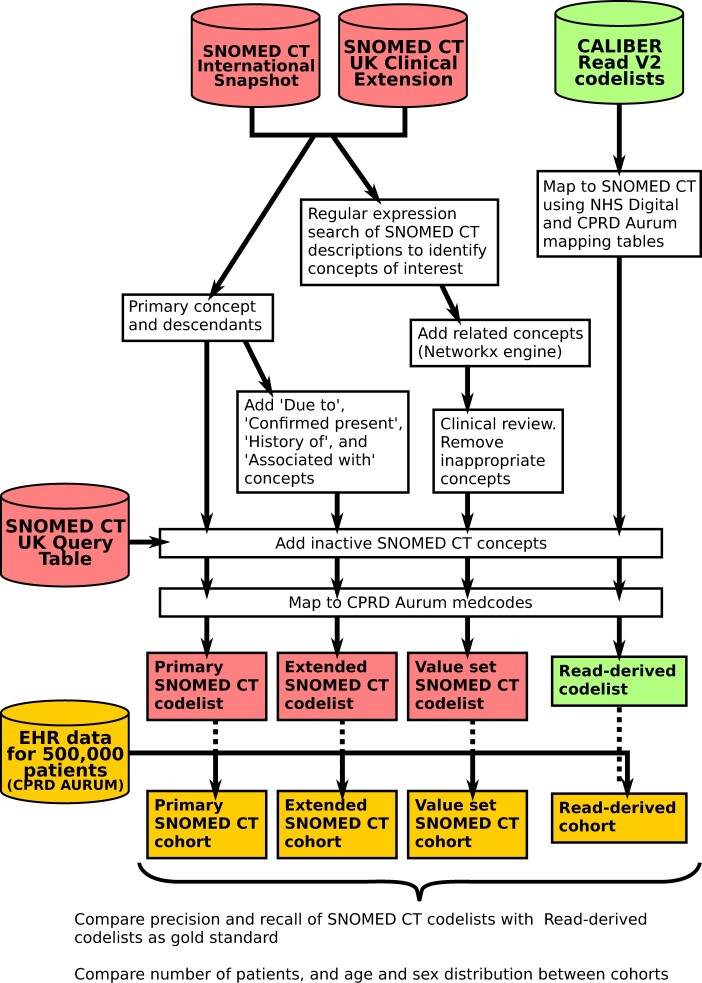
Flow diagram showing the creation and validation of “primary,” “extended,” and “value set” SNOMED CT codelists for diabetes, heart failure, and asthma.

**Figure 2.**
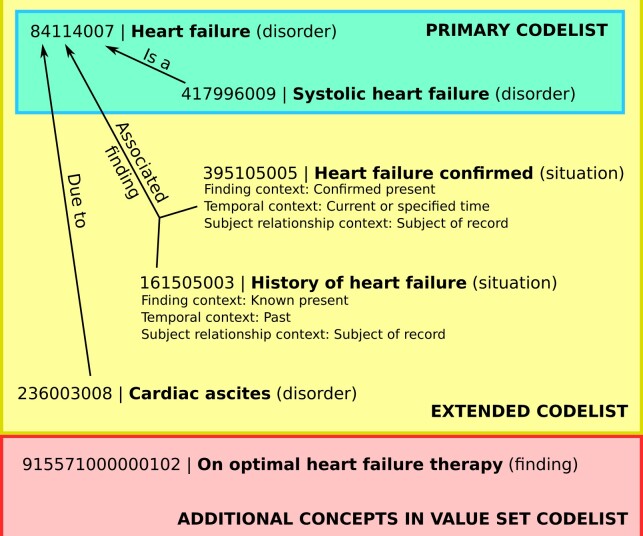
Examples of SNOMED CT concepts related to heart failure, showing which would be included in codelists created using the “primary,” “extended,” and “value set” methods.

For each disorder, we created codelists for phenotype definitions using 3 methods—a concise method using just the “is a” hierarchy (“primary”), a method that uses additional SNOMED CT relationships (“extended”), and a thorough but more labor-intensive method involving manual searches of term descriptions (“value set”) (Figures 1 and 2).

For the “value set” method, we aimed to identify all relevant SNOMED CT concepts regardless of their place in the hierarchy, by performing a keyword search of SNOMED CT descriptions. We used the SNOMED CT knowledge model to simplify the list of concepts by subsumption, and manually reviewed the list to decide if each concept would be included. This broad approach aimed to achieve complete coverage of phenotype-related codes including diagnostic findings, test findings, complications, related diseases, management, procedures, and follow-up. We additionally defined exclusion criteria for each codelist, consisting of historical, possible, suspected, or negated concepts, which were excluded from the final phenotype definition. We used python Networkx-based methods to explore the SNOMED CT hierarchy, develop SNOMED CT expressions, and visualize the connections between concepts.

For the “primary” method, the inclusion codelist consisted of a single concept describing the disease (“195967001 | Asthma [disorder],” “84114007 | Heart failure [disorder],” or “73211009 | Diabetes mellitus [disorder]”) and its descendants. For diabetes, we excluded “11687002 | Gestational diabetes mellitus (disorder)” and its descendants, to match the intent of the reference Read codelist, which did not include gestational diabetes. None of the descendants of the primary concept were excluded for the asthma or heart failure codelists.

We also created “extended” codelists including: (1) all concepts in the primary codelist, (2) all concepts related to the primary concept hierarchy using with the “Due to” or “Associated with” relationships, and (3) concepts linked by the relationship “Associated finding” with the additional criteria: “Finding context” attribute equals “Confirmed present” or “Known present,” and “Subject relationship context” equals “Subject of record.” The third category included concepts such as “Heart failure confirmed” and “History of heart failure,” which have the “situation” semantic tag and are not descendants of the “Heart failure” concepts. The aim of the extended codelists was to encompass as many concepts as possible that imply that the patient currently has the diagnosis or experienced it in the past.

For each codelist, we added inactive SNOMED CT concepts using the Query Table (Figure 1), because historic EHR data in CPRD may include inactive concepts.

### Phenotypes from the chronological map of human health

We developed an R script to download and process Read Version 2 codelists from a GitHub repository (https://github.com/spiros/chronological-map-phenotypes) for 276 diseases defined using primary care data in a study of diseases across the lifecourse.^33^ Each codelist consisted of Read terms for the disease diagnosis or history of the disease (terms for suspected diseases were not included). We used NHS Digital mapping files and the CPRD Aurum dictionary to map the Read codelists to SNOMED CT concepts, and denoted these converted codelists “Read-derived.”

**Figure 3.**
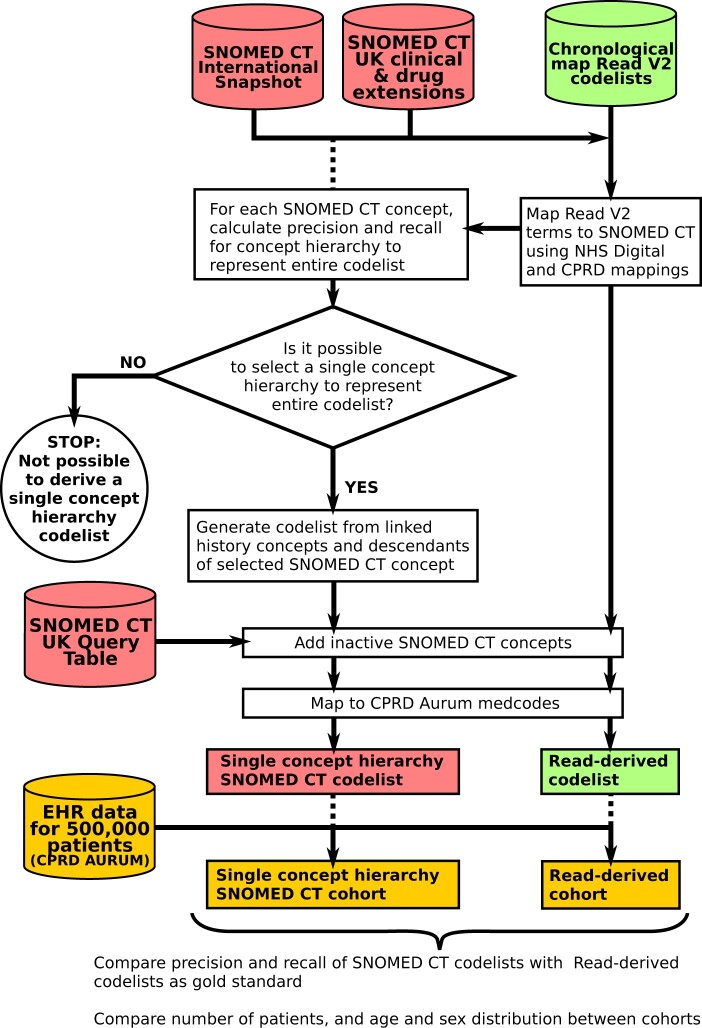
Flow diagram showing the creation and testing of SNOMED CT codelists for 276 conditions in the chronological map of health study.

We also sought to derive a parsimonious SNOMED CT expression to define each phenotype. For each mapped concept, parent of a mapped concept, or descendant of a mapped concept, we calculated the precision and recall for using the concept and its descendants to represent the entire codelist (Figure 3 and Table 1). We clinically reviewed the highest performing concepts for each phenotype, and judged whether any of these concepts had the same meaning as the phenotype itself. We used the functionality of the Rdiagnosislist package to display SNOMED CT hierarchies to assist this process. If it was possible to find a suitable concept to represent the entire codelist, this concept was selected and the codelist was generated using SNOMED CT relationships (“is a” descendants and linked personal history and confirmed situation concepts). Inactive SNOMED CT concepts were then added using the Query Table. We included personal history concepts as the codelists were intended to be used for classifying disease prevalence or history; definitions for incident disease should not include personal history concepts.

**Table 1.** Example of semiautomated codelist conversion from Read Version 2 to SNOMED CT, by calculating concept-based precision and recall for the hierarchy of each mapped concept and its parents. In this example, the SNOMED CT hierarchy for the concept “12295008 | Bronchiectasis” also includes Kartagener syndrome, which is not included in the Read codelist, hence the precision is 0.83 instead of 1

Read term(s)	SNOMED CT concept	Precision	Recall	F1 score
H34.00 Bronchiectasis	**12295008 | bronchiectasis (disorder)**—automatically selected as best match (highest F1 score)	0.83	1	0.91
H34z.00 Bronchiectasis NOS				
A115.00 Tuberculous bronchiectasis	23022004 | tuberculous bronchiectasis (disorder)	1	0.2	0.33
P861.00 Congenital bronchiectasis	77593006 | congenital bronchiectasis (disorder)	1	0.2	0.33
H340.00 Recurrent bronchiectasis	195984007 | recurrent bronchiectasis (disorder)	1	0.2	0.33
H341.00 Postinfective bronchiectasis	195985008 | post-infective bronchiectasis (disorder)	1	0.2	0.33
(Parent of mapped concept)	187251001 | sequelae of tuberculosis (disorder)	0.17	0.2	0.18
(Parent of mapped concept)	41427001 | disorder of bronchus (disorder)	0.05	1	0.1
(Parent of mapped concept)	123976001 | post-infectious disorder (disorder)	0.02	0.4	0.03
(Descendant of mapped concept)	42402006 | Kartagener syndrome (disorder)	0	0	0

### Evaluation of phenotypes

We extracted the earliest recorded diagnosis for each condition for patients in the study population using different phenotype definitions. We compared the precision, recall, and F1 scores of SNOMED CT phenotype definitions in selecting patient cohorts, taking the Read-derived cohorts as the gold standard. We also compared the number of patients, mean age, and sex distribution of cohorts defined using different methods.

### Ethics statement

CPRD has overarching annual research ethics approval from the United Kingdom’s Health Research Authority Research Ethics Committee (East Midlands—Derby, reference number: 05/MRE04/87). The study was approved by the MHRA (UK) Independent Scientific Advisory Committee (protocol number: 20_170R_ISAC).

## RESULTS

### Study population

The sample used in analysis consisted of 237 122 men and 262 878 women with a mean (SD) age of 40.2 (19.1) years at the mid-point of their registration period.

### Diabetes mellitus, asthma, and heart failure phenotypes

**Figure 4.**
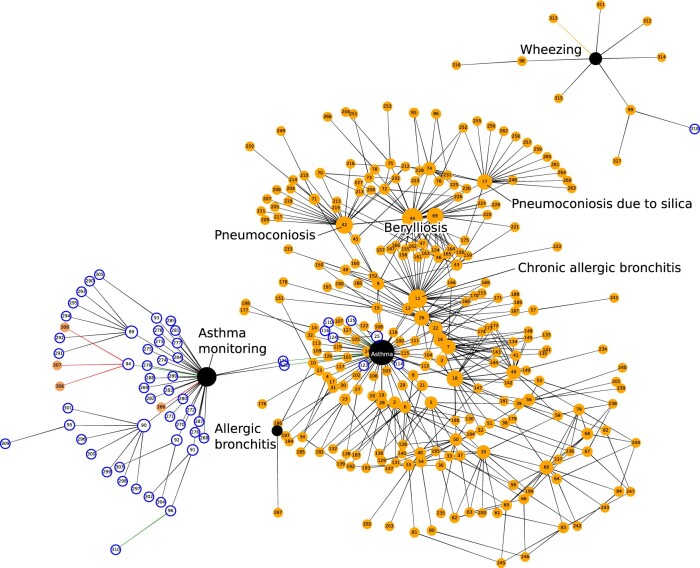
Concept network diagram for the asthma “value set” phenotype definition, with key concepts labeled. Key to node colors: black with white text = primary concept, orange = linked finding or disorder, white with blue outline = linked concepts of other semantic types eg, procedure or regime/therapy. Edges represent relationship types: black = “Is a,” green = “Has focus,” red = “Associated procedure,” and orange = “Associated finding.”

We successfully created value set SNOMED CT-based phenotype definitions based on keyword searches for words relevant to diabetes mellitus (Supplementary Table S1), asthma (Supplementary Table S2, Figure 4), and heart failure (Supplementary Table S3). The total number of concepts in the codelist was for 258 diabetes, 53 for asthma, and 44 for heart failure. The majority of concepts had the semantic tag “disorder,” comprising 58.5%–96.6% of concepts in each codelist. Other semantic types comprised a small number of concepts in each codelist except for the asthma value set, which contained 12 concepts (22.6%) with the semantic tag “regime/therapy” (Supplementary Table S4, Figure 4).

The vast majority of SNOMED CT concepts and patients were obtained by using “is a” SNOMED CT relationships with primary concepts (Table 2). Among diabetes concepts, the relationship “Has focus” selected 2051 additional patients (7.69%) compared to using “is a” relationships alone. The majority of these additional patients (1820) were selected by the “Diabetes mellitus screening” concept, but some of these patients may not actually have diabetes (if they underwent screening and the screening test was negative).

**Table 2.** Number of SNOMED CT concepts, medcodes, and patients included using different relationships in value set phenotype definitions. Among concepts linked to “Diabetes mellitus” by the relationship “Has focus,” the concept “Diabetes mellitus screening” captured the greatest number of additional patients (1820)

Phenotype	Relationship	*N* concepts	*N* CPRD medcodes	*N* patients	*N* (%) additional patients compared to self and “is a”
Diabetes mellitus	Self and “Is a”	131	409	26 681	–
	Associated procedure	10	16	5469	47 (0.18%)
	Due to	70	338	2590	30 (0.11%)
	Associated with	40	115	7308	40 (0.15%)
	Has focus	4	8	7991	2051 (7.69%)
	Associated finding	2	4	1107	4 (0.01%)
	Associated etiologic finding	1	1	57	1 (< 0.01%)
	All	258	895	28 849	2168 (8.13%)
Asthma	Self and “Is a”	49	110	57 287	–
	Has focus	4	4	784	39 (0.07%)
	All	53	114	57 326	39 (0.07%)
Heart failure	Self and “Is a”	43	84	9501	–
	Associated finding	1	1	82	20 (0.21%)
	All	44	85	9521	20 (0.21%)

Definitions for primary codelists in tabular form and as SNOMED CT expressions are given in Supplementary Table S5, and definitions for extended codelists are in Supplementary Table S6. Interactive HTML documents for exploring the extended codelist hierarchy are in Supplementary File 2 (diabetes mellitus), Supplementary File 3 (asthma), and Supplementary File 4 (heart failure).

Cohorts derived from value set codelists included more patients compared to those derived from primary or extended codelists. Considering Read-derived codelists as the gold standard, value set codelists had higher recall, but slightly lower precision than primary or extended codelists (Table 3). F1 scores exceeded 0.93 for all comparisons.

**Table 3.** Performance of different SNOMED CT-based phenotype definitions, compared to the original manually curated Read V2 codelists as a gold standard

Phenotype	Method	*N* concepts	*N* CPRD medcodes	*N* patients	Precision (95% CI)	Recall (95% CI)	F1 score
Diabetes mellitus	Read	216	784	28 233			
	Primary	152	593	24 882	0.999 (0.998–0.999)	0.880 (0.876–0.884)	0.936
	Extended	204	779	25 214	0.988 (0.987–0.990)	0.883 (0.879–0.886)	0.933
	Value Set	258	739	28 849	0.921 (0.918–0.924)	0.941 (0.938–0.944)	0.931
Asthma	Read	25	69	51 020			
	Primary	34	99	52 593	0.969 (0.967–0.970)	0.999 (0.998–0.999)	0.984
	Extended	36	102	55 552	0.918 (0.916–0.920)	1.000 (1.000–1.000)	0.957
	Value Set	53	107	57 326	0.890 (0.887–0.892)	1.000 (0.999–1.000)	0.941
Heart failure	Read	38	78	9454			
	Primary	27	54	8474	0.995 (0.993–0.996)	0.892 (0.885–0.898)	0.941
	Extended	30	59	8546	0.990 (0.988–0.992)	0.895 (0.888–0.901)	0.940
	Value Set	44	76	9521	0.982 (0.980–0.985)	0.989 (0.987–0.991)	0.986

There were minimal differences in the age and sex distributions between cohorts defined using the different methods, although some differences were statistically significant because of the large sample size. For the diabetes phenotype, the primary and extended cohorts had a slightly lower proportion of female patients than the Read cohort (difference in proportions 0.012 or 0.010, respectively). Some of the SNOMED CT asthma and heart failure cohorts were younger than cohorts selected using Read, for example the primary asthma cohort was mean 0.36 years (95% CI: 0.09–0.64) younger and the primary heart failure cohort was 0.67 years (95% CI; 0.29–1.05) younger than the Read cohort (Table 4).

**Table 4.** Cohort characteristics based on different SNOMED CT-based phenotype definitions, and comparison with the original manually curated Read V2 codelists

Phenotype	Method	*N* patients	*N* (%) female	Difference in sex proportion (95% CI)	*P* value	Mean (SD) age	Difference in mean age (95% CI)	*P* value
Diabetes mellitus	Read	28 233	13 051 (46.2)	–	–	56.9 (19.0)	–	–
	Primary	24 882	11 207 (45.0)	0.012 (0.003–0.020)	.006	57.0 (18.6)	–0.09 (–0.41 to 0.23)	.58
	Extended	25 214	11 411 (45.3)	0.010 (0.001–0.018)	.025	56.9 (18.6)	0.04 (–0.28 to 0.36)	.80
	Value set	28 849	13 327 (46.2)	0.000 (–0.008 to 0.008)	.95	56.8 (18.6)	0.08 (–0.23 to 0.39)	.61
Asthma	Read	51 020	27 312 (53.5)	–	–	26.1 (22.4)	–	–
	Primary	52 593	28 164 (53.6)	0.000 (–0.006 to 0.006)	.96	26.5 (22.7)	–0.36 (–0.64 to –0.09)	.010
	Extended	55 552	29 662 (53.4)	0.001 (–0.005 to 0.007)	.66	26.2 (22.5)	–0.13 (–0.40 to 0.14)	.36
	Value set	57 326	30 742 (53.6)	–0.001 (–0.007 to 0.005)	.76	27.7 (23.3)	–1.62 (–1.89 to –1.34)	<.001
Heart failure	Read	9454	4596 (48.6)	–	–	75.2 (13.1)	–	–
	Primary	8474	4229 (49.9)	–0.013 (–0.028 to 0.002)	.087	75.9 (12.9)	–0.67 (–1.05 to –0.29)	<.001
	Extended	8546	4259 (49.8)	–0.012 (–0.027 to 0.003)	.10	75.8 (13.0)	–0.59 (–0.97 to –0.21)	.002
	Value set	9521	4606 (48.4)	0.002 (–0.012 to 0.017)	.75	75.0 (13.5)	0.22 (–0.16 to 0.60)	.25

### Phenotypes from the chronological map of human health

On manual review of the Read to SNOMED CT mappings, we were able to represent 257 of the 276 phenotypes with a SNOMED CT concept, and in 59% (151/257), this was the concept that was suggested automatically (the concept hierarchy with the highest F1 score for the inclusion of SNOMED CT concepts against the gold standard of the original Read codelist mapped to SNOMED CT). Some phenotypes could not be represented as a single concept hierarchy because they are a collection of different diseases (eg, “enteropathic arthropathies”), or because they are defined by exclusion (eg, “other hemolytic anemia,” “stroke not otherwise specified”) (Supplementary Table S7). Full results are included in Supplementary File 5.

For 29 phenotypes, the SNOMED CT concept hierarchy selected an identical cohort to the mapped Read codelist, and in 135 (52.5%), the F1 score was greater than 0.9.

## DISCUSSION

### Summary of main findings

By examining 276 diseases, we have demonstrated that the SNOMED CT knowledge model can simplify the process of selecting patient cohorts and outcomes in EHR research databases, and enable the transformation of historic phenotyping algorithms. In cases where the SNOMED CT knowledge model corresponds well to the researcher’s intended definition of a phenotype, it may be possible to represent the phenotype using a single concept hierarchy. For the examples of diabetes mellitus, heart failure, and asthma, patient cohorts derived using different Read and SNOMED CT phenotyping methods were very similar. We have also produced an R package^34^ with sample code (Supplementary File 6) to enable researchers to easily adopt these methods to develop their own codelists in SNOMED CT, or convert codelists from other terminologies such as Read.

### Defining phenotypes using SNOMED CT

Conventionally, phenotype and outcome definitions consist of an enumerated list of terminology concepts (“codelist,” “code set,” or “value set”) created by the manual process of keyword searching, which does not take into account the ontology contained within the terminology system.^1^ Consistent with previous studies, we found that the SNOMED CT ontology can simplify the process of producing diagnosis codelists that are concise, understandable to clinicians, and useful for data analytics.^8^^,^^19^ We have extended these findings to a large EHR database containing data encoded using both Read V2 and SNOMED CT from hundreds of primary care practices in the United Kingdom. We have also developed semiautomated methods for converting a Read V2 codelist into a parsimonious SNOMED CT phenotype definition, implemented in an R package.^34^

Terminologies change over time as medical knowledge changes or errors in the terminology system are corrected.^14^ A codelist defined using SNOMED CT expressions or hierarchy will automatically include new concepts if they are modeled in an appropriate place in the hierarchy, and SNOMED CT enabled EHR systems could enable patient populations for decision support, audit, or research to be defined in a parsimonious way.

The principal parent–child “is a” relationship enabled the majority of relevant concepts to be captured. Additional concepts could be included by using other relationships, for example “Due to” could enable inclusion of concepts for which the current concept is a sequela or consequence, and “Associated with” provides a link to procedures associated with a diagnosis. SNOMED CT concepts with the semantic tag “situation” include concepts for suspected and historic conditions, as well as a few concepts for confirmed disorders, which may be important in some cases. For example, the latest UK release of SNOMED CT contains 2 important “situation” concepts related to COVID-19: “1300721000000109 | COVID-19 confirmed by laboratory test” and “1300731000000106 | COVID-19 confirmed using clinical diagnostic criteria,” which are related to, but not direct subtypes of “840539006 | Disease caused by Severe acute respiratory syndrome coronavirus 2 (disorder)” (the main COVID-19 diagnosis concept).

### Recommendations for the use of SNOMED CT in defining research phenotypes

Based on our findings, we recommend the use of SNOMED CT concept hierarchies and attributes for generating phenotype definitions in a parsimonious, explainable, and reproducible way. However, there researchers need to be aware of the following caveats:

First, SNOMED CT concepts can become inactive but may have been used to record historic data that exist in research databases. There may be no current equivalent for the meaning of some inactive concepts (if they are ambiguous or deprecated). In this project, we included inactive SNOMED CT concepts using the NHS Digital Query Table, which categorizes the provenance of the link on a 4-level scale (ranging from 0 = subsumption is always true, to 3 = original concept had at least 2 distinct meanings).

Second, the researcher or clinician may have a different intent regarding the inclusion of subtypes compared to the SNOMED CT hierarchy, such as whether gestational diabetes is included within a diabetes phenotype.

Third, SNOMED CT semantic types may not be adhered to strictly when data are entered, which means that essential information may not be in the expected location in the hierarchy. For example, cancer diagnoses may have been recorded using “morphologic abnormality” concepts which are intended for use on histology reports. Semantic tags are not always consistent with the SNOMED CT hierarchy within SNOMED CT itself.^36^

Fourth, the SNOMED CT methods rely on complete and accurate relationships between concepts being modeled within the SNOMED CT ontology. While this is broadly the case for diagnoses, concepts in other domains (eg, symptoms/findings) may not be as well modeled, and it is possible that relevant terms may be overlooked if researchers rely solely on the hierarchy. We recommend that ongoing engagement between clinicians, researchers, and SNOMED CT editors should continuously improve the quality of the SNOMED CT model.

### Limitations of this study

A limitation of this study is that we considered the existing Read V2 phenotype definitions as the gold standard, and assumed that they were complete and accurate based on the manual review process involved in creating them. Thus it was assumed that any additional Read V2 terms selected by the SNOMED CT method and mapping were extraneous, but this may not have been the case.

Second, we relied on accurate mappings between SNOMED CT and Read V2 as supplied by NHS Digital and CPRD in order to ensure that codelists created using different terminologies had the same meaning.

Third, we did not have access to patients’ original text notes, and were unable to verify the accuracy of the original Read or SNOMED CT concepts entered in the health records. CPRD does not provide researchers with access to the free text within EHRs because of concerns over confidentiality. However, previous validation studies using the primary care databases^37^ have shown that coded diagnoses are likely to be correct when present, but may be incomplete and may not use the most specific, precise term available.

Fourth, our study was limited to the primary care setting. We assume that SNOMED CT coding in secondary care settings will be similarly accurate in order to enable data to be used for research, but it will be important to validate the accuracy and completeness of coding.

## CONCLUSION

In conclusion, the SNOMED CT knowledge model provides an efficient way to define disease phenotypes in EHR databases. We have demonstrated that the method can result in similar patient populations compared to manually curated codelists, and we have developed tools to assist researchers in making use of these models. For some disease phenotypes, a simple definition based on a single concept hierarchy may be sufficient to define the phenotype. These methods may facilitate the use of EHR data for research and improving patient care.

## FUNDING

This work was supported by Health Data Research UK, which receives its funding from the UK Medical Research Council, Engineering and Physical Sciences Research Council, Economic and Social Research Council, Department of Health and Social Care (England), Chief Scientist Office of the Scottish Government Health and Social Care Directorates, Health and Social Care Research and Development Division (Welsh Government), Public Health Agency (Northern Ireland), British Heart Foundation, and the Wellcome Trust. This study was supported by National Institute for Health Research (RP-PG-0407-10314) and Wellcome Trust (086091/Z/08/Z). ADS is funded by a postdoctoral fellowship from THIS Institute, NIHR (AI_AWARD01864 and COV-LT-0009), UKRI (Horizon Europe Guarantee for DataTools4Heart) and British Heart Foundation Accelerator Award (AA/18/6/24223). VK is supported by the UKRI/NIHR Strategic Priorities Award in Multimorbidity Research (MR/V033867/1) for the Multimorbidity Mechanism and Therapeutics Research Collaborative. RTL is supported by a UKRI Rutherford Fellowship. SD is supported by: (1) Health Data Research UK London, which receives its funding from HDR UK funded by the UK MRC, EPSRC, ESRC, Department of Health and Social Care (England), Chief Scientist Office of the Scottish Government Health and Social Care Directorates, Health and Social Care Research and Development Division (Welsh government), Public Health Agency (Northern Ireland), BHF, and Wellcome Trust; (2) The NIHR Biomedical Research Centre at University College London Hospital NHS Trust; (3) The Alan Turing Institute (EP/N510129/1); (4) The British Heart Foundation Accelerator Award (ref AA/18/6/24223); (5) The BigData@Heart Consortium, funded by the Innovative Medicines Initiative-2 Joint Undertaking under grant agreement (ref 116074); (6) The British Heart Foundation Data Science Centre (ref SP/19/3/34678); (7) The UKRI/NIHR funded Multimorbidity Mechanism and Therapeutics Research Collaborative (MR/V033867/1); (8) The Longitudinal Health and Wellbeing COVID-19 National Core Study, which was established by the UK Chief Scientific Officer in October 2020 and funded by UK Research and Innovation (grant references MC_PC_20030 and MC_PC_20059); (9) The Data and Connectivity National Core Study, led by Health Data Research UK in partnership with the Office for National Statistics and funded by UK Research and Innovation (grant reference MC_PC_20058); and (10) The CONVALESCENCE study of long COVID, which is funded by NIHR/UKRI.

## AUTHOR CONTRIBUTIONS

ADS devised the protocol, sought approvals and supervised the project. ME, AG, MQ, and ADS analyzed the data. ME drafted the manuscript. All authors contributed intellectual content and approved the final manuscript. ADS is guarantor.

## SUPPLEMENTARY MATERIAL

Supplementary material is available at *Journal of the American Medical Informatics Association* online.

## Supplementary Material

ocac158_Supplementary_Data

## Data Availability

The data underlying this article were provided by the Clinical Practice Research Database (CPRD) under license and cannot be shared publicly in order to protect patient confidentiality. Data can only be provided with the approval of the CPRD’s Research Data Governance (RDG) Process.
