# Supplementary material for: Translating and evaluating historic phenotyping algorithms using SNOMED CT
Source: J Am Med Inform Assoc. 2022 Sep 9;30(2):222–32. doi: 10.1093/jamia/ocac158 (PMC9846670; doi:10.1093/jamia/ocac158)
Supplement: ocac158_Supplementary_Data [file ocac158_supplementary_data.zip › ocac158_Supplementary_Data/Supp_1_Tables.pdf]

Translating and evaluating historic phenotyping algorithms using SNOMED CT:

Supplementary tables

Supplementary Table 1. SNOMED CT concepts used to define diabetes mellitus value set codelist

Concept hierarchies for inclusion in value set	Concept hierarchies used for exclusion
73211009 | Diabetes mellitus (disorder) | 415744000 | Transition of diabetes care options discussed (situation) | 394725008 | Diabetes medication review (procedure) | 170742000 | Diabetic monitoring (regime/therapy) | 365845005 | Hemoglobin A1C - diabetic control finding (finding) | 407569005 | Patient on maximal tolerated therapy for diabetes (finding) | 308755006 | Subcutaneous injection of insulin (procedure) | 308253000 | Admission to diabetic department (procedure) | 183751004 | Diabetology domiciliary visit done (finding) | 183472000 | Diabetic emergency hospital admission (procedure) | 160670007 | Diabetic diet (finding) | 313987006 | Retinopathy follow up (finding) | 870681000000109 | In-house diabetic foot screening (procedure) | 756391000000103 | On examination - Right diabetic foot at increased risk (finding) | 756381000000100 | On examination - Left diabetic foot at increased risk (finding) | 394681008 | On examination - Left diabetic foot at moderate risk (finding) | 394676004 | On examination - Left diabetic foot at high risk (finding) | 394675000 | On examination - Left diabetic foot at low risk (finding) | 394674001 | On examination - Left diabetic foot - ulcerated (finding) | 394673007 | On examination - Right diabetic foot - ulcerated (finding) | 394672002 | On examination - Right diabetic foot at high risk (finding) |	439051004 | Dietary education for gestational diabetes (procedure) | 472699005 | Gestational diabetes mellitus uncontrolled (finding) | 472971004 | History of gestational diabetes mellitus (situation) | 702737001 | Supervision of high risk pregnancy with history of gestational diabetes mellitus (regime/therapy) | 85031000119106 | Supervision of high risk pregnancy with history of gestational diabetes mellitus done (situation) | 764921000000102 | Gestational diabetes information leaflet given (situation) | 816261000000105 | Management of gestational diabetes mellitus (procedure) | 816711000000104 | Gestational diabetes mellitus annual review (regime/therapy) | 837811000000101 | Provision of written information about gestational diabetes (procedure) | 266990005 | History of endocrine disorder (situation) | 281666001 | Family history of disorder (situation) | 160402005 | Family history: Diabetes in pregnancy (situation) | 160274005 | No family history diabetes (situation) |

Supplementary Table 2. SNOMED CT concepts used to define asthma value set codelist

Concept hierarchies for inclusion in value set	Concept hierarchies used for exclusion
195967001 | Asthma (disorder) | 405720007 | Allergic bronchitis (disorder) | 275908000 | Asthma monitoring (regime/therapy) | 56018004 | Wheezing (finding) |	928651000000105 | Review of patient at risk of asthma (procedure) | 928691000000102 | At risk of asthma (finding) | 161527007 | History of asthma (situation) | 704008007 | No family history of asthma (situation) | 160377001 | Family history: Asthma (situation) |

Supplementary Table 3. SNOMED CT concepts used to define heart failure value set codelist

**Concept hierarchies for inclusion in value set**	**Concept hierarchies used for exclusion**
84114007 | Heart failure (disorder) | 915571000000102 | On optimal heart failure therapy (finding) | 871681000000102 | Heart failure monitoring (regime/therapy) | 202231000000106 | Heart failure review completed (situation) | 429589006 | Left ventricular cardiac dysfunction (disorder) | 416683003 | Emergency hospital admission for heart failure (procedure) | 275514001 | Impaired left ventricular function (finding) | 390885007 | Heart failure annual review (regime/therapy) | 473226007 | Heart failure medication review (procedure) | 810971000000105 | Heart failure self-management plan review (procedure) | 195021004 | Primary dilated cardiomyopathy (disorder) | 91604001 | Dilated cardiomyopathy secondary to polyarteritis nodosa (disorder) | 399020009 | Congestive cardiomyopathy (disorder) | 702424003 | Dilated cardiomyopathy 3B (disorder) | 83521008 | Dilated cardiomyopathy caused by alcohol (disorder) | 719451006 | Dilated cardiomyopathy with hypergonadotropic hypogonadism syndrome (disorder) | 719835006 | Wooly hair and palmoplantar keratoderma with dilated cardiomyopathy syndrome (disorder) | 15629591000119103 | Congestive heart failure stage B due to ischemic cardiomyopathy (disorder) | 15629541000119106 | Congestive heart failure stage C due to Ischemic cardiomyopathy (disorder) | 723993005 | Sensorineural deafness with dilated cardiomyopathy syndrome (disorder) | 766883006 | Familial dilated cardiomyopathy with conduction defect due to lamin A/C mutation (disorder) | 111000119104 | Nonischemic congestive cardiomyopathy (disorder) | 74249003 | Dilated cardiomyopathy secondary to malignancy (disorder) | 11104006 | Dilated cardiomyopathy secondary to Refsum's disease (disorder) | 111285003 | Dilated cardiomyopathy secondary to metabolic disorder (disorder) | 20529002 | Secondary dilated cardiomyopathy (disorder) | 6022005 | Dilated cardiomyopathy secondary to sarcoidosis (disorder) | 101281000119107 | Congestive heart failure due to cardiomyopathy (disorder) | 46696008 | Dilated cardiomyopathy secondary to dermatomyositis (disorder) |	394926003 | Heart disease excluded (situation) | 266995000 | History of cardiovascular disease (situation) | 281666001 | Family history of disorder (situation) | 198381000000104 | Family history of hypertrophic obstructive cardiomyopathy (situation) |

Supplementary Table 4. Distribution of SNOMED CT concepts by semantic tag for diabetes, asthma and heart failure value set codelists

Phenotype	Semantic tag	Number of concepts	Percentage
Diabetes mellitus	disorder	185	71.7
	finding	27	10.5
	procedure	11	4.3
	regime/therapy	17	6.6
	situation	18	7.0
Asthma	disorder	31	58.5
	finding	5	9.4
	procedure	3	5.7
	regime/therapy	12	22.6
	situation	2	3.8
Heart failure	disorder	36	81.8
	finding	2	4.5
	procedure	2	4.5
	regime/therapy	2	4.5
	situation	2	4.5

Supplementary Table 5. Definition of ‘primary’ codelists for diabetes mellitus, asthma and heart failure. These codelists contain only the primary concept and its descendants, with exclusion of gestational diabetes in the case of the diabetes mellitus phenotype.

Phenotype	SNOMED CT concept	With descendants?	Include / exclude
Diabetes mellitus	**Diabetes mellitus (disorder)**	**Yes**	**Include**
	**Gestational diabetes mellitus (disorder)**	**Yes**	**Exclude**
Asthma	**Asthma (disorder)**	**Yes**	**Include**
Heart failure	**Heart failure (disorder)**	**Yes**	**Include**

The codelists can also be represented in SNOMED CT Expression Constraint Language, where ‘<<’ means ‘descendants or self of’:

Diabetes mellitus: << 73211009 | Diabetes mellitus (disorder) | MINUS

<< 11687002 | Gestational diabetes mellitus (disorder) |

Asthma: << 195967001 | Asthma (disorder) |

Heart failure: << 84114007 | Heart failure (disorder) |

Supplementary Table 6. Definition of ‘extended’ codelists for diabetes mellitus, asthma and heart failure. These codelists contain descendants of the primary concepts, associated history and situation concepts, and disorders that are ‘due to’ the primary disorder according to the SNOMED CT ontology. The codelists are also available in HTML format to browse in Supplementary Files 2, 3 and 4.

Phenotype	SNOMED CT concept	With descendants?	Include / exclude?
Diabetes mellitus	**Diabetes mellitus (disorder)**	**Yes**	**Include**
	**Gestational diabetes mellitus (disorder)**	**Yes**	**Exclude**
	**Diabetic complication (disorder)**	**Yes**	**Include**
	**History of diabetes mellitus (situation)**	**Yes**	**Include**
	History of gestational diabetes mellitus (situation)	No	Exclude
	**History of admission in last year for diabetes foot problem (situation)**	**Yes**	**Include**
	History of diabetic foot ulcer (situation)	No	Include
	History of diabetic ketoacidosis (situation)	No	Include
	History of diabetic peripheral angiopathy (situation)	No	Include
	History of nocturnal hypoglycemia (situation)	No	Include
	Hyperosmolarity due to type 1 diabetes mellitus (disorder)	No	Include
	On examination - diabetic maculopathy present both eyes (situation)	No	Include
Asthma	**Asthma (disorder)**	**Yes**	**Include**
	**History of asthma (situation)**	**Yes**	**Include**
	Asthma confirmed (situation)	No	Include
Heart failure	**Heart failure (disorder)**	**Yes**	**Include**
	**History of heart failure (situation)**	**Yes**	**Include**
	Cardiac ascites (disorder)	No	Include
	Cardiac edema (disorder)	No	Include
	Heart failure confirmed (situation)	No	Include
	Pleural effusion due to congestive heart failure (disorder)	No	Include

Supplementary Table 7. Summary of SNOMED CT codelists for 276 primary care disease phenotypes in ‘chronological map’ project. Full results (excluding phenotypes with small numbers) are shown in Supplementary File 5.

Phenotype can be represented by a single concept hierarchy?	Performance in cohort definition compared to Read V2 codelist (gold standard)	Number of phenotypes	Example phenotype	Example SNOMED CT concept
No	N/A	19	‘AF’ (atrial flutter or fibrillation)	No map to a single SNOMED CT concept, as it is a collection of two disorders
Yes	Exact (identical cohorts)	29	‘pcos’ (polycystic ovarian syndrome)	69878008 | polycystic ovaries (disorder)
	F1 score ≥ 0.95	73	‘depression’ (depression)	35489007 | Depressive disorder (disorder)
	F1 score ≥ 0.90 and < 0.95	33	‘deaf’ (hearing loss or procedure for hearing loss)	15188001 | Hearing loss (disorder)
	F1 score ≥ 0.80 and < 0.90	27	‘anxiety’ (anxiety disorders)	197480006 | Anxiety disorder (disorder)
	F1 score ≥ 0.50 and < 0.80	47	‘periph_neuro’ (peripheral neuropathy)	42658009 | Disorder of the peripheral nervous system (disorder)
	F1 score < 0.50	48	‘benign_uterus’ (benign neoplasm or polyp of uterus)	92470003 | Benign neoplasm of uterus (disorder)
