# Supplementary material for: Translating and evaluating historic phenotyping algorithms using SNOMED CT
Source: J Am Med Inform Assoc. 2022 Sep 9;30(2):222–32. doi: 10.1093/jamia/ocac158 (PMC9846670; doi:10.1093/jamia/ocac158)
Supplement: ocac158_Supplementary_Data [file ocac158_supplementary_data.zip › ocac158_Supplementary_Data/Supp_2_dm_ext.html]

Extended SNOMED CT codelist for diabetes mellitus (excluding gestational diabetes)

# Extended SNOMED CT codelist for diabetes mellitus (excluding gestational diabetes)

## Instructions

This HTML document presents a hierarchy of SNOMED CT concepts.
In SNOMED CT, each concept has a distinct meaning and can
be linked to more general terms (ancestors) and more specific terms (descendants). The buttons allow you to explore the codelist at
different levels of the hierarchy, and mark whether or not you agree
with the inclusion of individual concepts or concept hierarchies. When
you have finished your review, you can download your final selection as a .CSV file by clicking the **Export** button below.

### Key to buttons for each concept

- Expand Show descendants of this concept
- Contract Hide descendants of this concept
- **?** Mark as unchecked
- **+** Add a concept
- **-** Remove a concept
- **++** Add a concept and all descendants
- **--** Remove a concept and all descendants

## Reviewing tools

Show top-level concepts only Show all concepts

**Mark all concepts as "checked"** **Mark all concepts as "unchecked"** Show unchecked concepts only

**Export** to
 .csv

| Expand | SNOMED CT concept | Comment | Checked | Included |  |
| --- | --- | --- | --- | --- | --- |
| Contract | **Diabetes mellitus (disorder)** | ... |  | Y | **?****+****-****++****--** |
| Contract | · **Acute complication co-occurrent and due to diabetes mellitus (disorder)** | ... |  | Y | **?****+****-****++****--** |
|  | · · Acute painful polyneuropathy co-occurrent and due to diabetes mellitus (disorder) | ... |  | Y | **?****+****-** |
|  | · Alaninuria, microcephaly, dwarfism, enamel hypoplasia, diabetes mellitus syndrome (disorder) | ... |  | Y | **?****+****-** |
|  | · Atypical diabetes mellitus (disorder) | ... |  | Y | **?****+****-** |
| Contract | · **Diabetes mellitus due to genetic defect in beta cell function (disorder)** | ... |  | Y | **?****+****-****++****--** |
| Contract | · · **Maturity-onset diabetes of the young (disorder)** | ... |  | Y | **?****+****-****++****--** |
|  | · · · Maturity onset diabetes of the young, type 1 (disorder) | ... |  | Y | **?****+****-** |
|  | · · · Maturity onset diabetes of the young, type 2 (disorder) | ... |  | Y | **?****+****-** |
|  | · · · Maturity-onset diabetes of the young, type 10 (disorder) | ... |  | Y | **?****+****-** |
|  | · · · Maturity-onset diabetes of the young, type 11 (disorder) | ... |  | Y | **?****+****-** |
|  | · · · Maturity-onset diabetes of the young, type 3 (disorder) | ... |  | Y | **?****+****-** |
|  | · · · Maturity-onset diabetes of the young, type 4 (disorder) | ... |  | Y | **?****+****-** |
|  | · · · Maturity-onset diabetes of the young, type 5 (disorder) | ... |  | Y | **?****+****-** |
|  | · · · Maturity-onset diabetes of the young, type 6 (disorder) | ... |  | Y | **?****+****-** |
|  | · · · Maturity-onset diabetes of the young, type 7 (disorder) | ... |  | Y | **?****+****-** |
|  | · · · Maturity-onset diabetes of the young, type 8 (disorder) | ... |  | Y | **?****+****-** |
|  | · · · Maturity-onset diabetes of the young, type 9 (disorder) | ... |  | Y | **?****+****-** |
| Contract | · · **Neonatal diabetes mellitus (disorder)** | ... |  | Y | **?****+****-****++****--** |
|  | · · · Developmental delay, epilepsy, neonatal diabetes syndrome (disorder) | ... |  | Y | **?****+****-** |
| Contract | · · · **Permanent neonatal diabetes mellitus (disorder)** | ... |  | Y | **?****+****-****++****--** |
|  | · · · · Permanent neonatal diabetes mellitus with cerebellar agenesis syndrome (disorder) | ... |  | Y | **?****+****-** |
|  | · · · · Primary microcephaly, epilepsy, permanent neonatal diabetes syndrome (disorder) | ... |  | Y | **?****+****-** |
| Contract | · · · **Transitory neonatal diabetes mellitus (disorder)** | ... |  | Y | **?****+****-****++****--** |
|  | · · · · Diabetes mellitus, transient neonatal 1 (disorder) | ... |  | Y | **?****+****-** |
|  | · · · · Diabetes mellitus, transient neonatal 2 (disorder) | ... |  | Y | **?****+****-** |
|  | · · · · Diabetes mellitus, transient neonatal 3 (disorder) | ... |  | Y | **?****+****-** |
| Contract | · **Diabetes mellitus due to genetic defect in insulin action (disorder)** | ... |  | Y | **?****+****-****++****--** |
|  | · · Extreme insulin resistance with acanthosis nigricans, hirsutism AND abnormal insulin receptors (disorder) | ... |  | Y | **?****+****-** |
|  | · · Insulin resistance - type A (disorder) | ... |  | Y | **?****+****-** |
|  | · · Insulin resistance - type B (disorder) | ... |  | Y | **?****+****-** |
|  | · · Leprechaunism syndrome (disorder) | ... |  | Y | **?****+****-** |
| Contract | · · **Lipoatrophic diabetes (disorder)** | ... |  | Y | **?****+****-****++****--** |
|  | · · · Lipoatrophic diabetes mellitus without complication (disorder) | ... |  | Y | **?****+****-** |
|  | · · Pineal hyperplasia AND diabetes mellitus syndrome (disorder) | ... |  | Y | **?****+****-** |
|  | · Diabetes mellitus due to pancreatic injury (disorder) | ... |  | Y | **?****+****-** |
| Contract | · **Diabetes mellitus during pregnancy, childbirth and the puerperium (disorder)** | ... |  | Y | **?****+****-****++****--** |
|  | · · Diabetes mellitus during pregnancy - baby delivered (disorder) | ... |  | Y | **?****+****-** |
|  | · · Diabetes mellitus during pregnancy - baby not yet delivered (disorder) | ... |  | Y | **?****+****-** |
| Contract | · · **Diabetes mellitus in mother complicating pregnancy, childbirth AND/OR puerperium (disorder)** | ... |  | Y | **?****+****-****++****--** |
| Contract | · · · **Diabetes mellitus in mother complicating childbirth (disorder)** | ... |  | Y | **?****+****-****++****--** |
|  | · · · · Pre-existing diabetes mellitus in mother complicating childbirth (disorder) | ... |  | Y | **?****+****-** |
|  | · · · Maternal diabetes mellitus with hypoglycemia affecting fetus OR newborn (disorder) | ... |  | Y | **?****+****-** |
|  | · · Diabetes mellitus in the puerperium - baby delivered during current episode of care (disorder) | ... |  | Y | **?****+****-** |
|  | · · Diabetes mellitus in the puerperium - baby delivered during previous episode of care (disorder) | ... |  | Y | **?****+****-** |
| Contract | · · **Gestational diabetes mellitus (disorder)** | ... |  | N | **?****+****-****++****--** |
|  | · · · Gestational diabetes mellitus complicating pregnancy (disorder) | ... |  | N | **?****+****-** |
|  | · · · Gestational diabetes mellitus in childbirth (disorder) | ... |  | N | **?****+****-** |
|  | · · · Gestational diabetes mellitus, class A>1< (disorder) | ... |  | N | **?****+****-** |
|  | · · · Gestational diabetes mellitus, class A>2< (disorder) | ... |  | N | **?****+****-** |
|  | · · · Postpartum gestational diabetes mellitus (disorder) | ... |  | N | **?****+****-** |
| Contract | · · **Pre-existing diabetes mellitus in pregnancy (disorder)** | ... |  | Y | **?****+****-****++****--** |
|  | · · · Pre-existing type 1 diabetes mellitus in pregnancy (disorder) | ... |  | Y | **?****+****-** |
|  | · · · Pre-existing type 2 diabetes mellitus in pregnancy (disorder) | ... |  | Y | **?****+****-** |
|  | · · Pregnancy and type 1 diabetes mellitus (disorder) | ... |  | Y | **?****+****-** |
|  | · · Pregnancy and type 2 diabetes mellitus (disorder) | ... |  | Y | **?****+****-** |
| Contract | · **Diabetes mellitus in remission (disorder)** | ... |  | Y | **?****+****-****++****--** |
|  | · · Type I diabetes mellitus in remission (disorder) | ... |  | Y | **?****+****-** |
|  | · · Type II diabetes mellitus in remission (disorder) | ... |  | Y | **?****+****-** |
| Contract | · **Diabetes mellitus type 1 (disorder)** | ... |  | Y | **?****+****-****++****--** |
|  | · · Blindness co-occurrent and due to type 1 diabetes mellitus (disorder) | ... |  | Y | **?****+****-** |
|  | · · Diabetes mellitus type 1 without retinopathy (disorder) | ... |  | Y | **?****+****-** |
| Contract | · · **Disorder of eye co-occurrent and due to type 1 diabetes mellitus (disorder)** | ... |  | Y | **?****+****-****++****--** |
|  | · · · Rubeosis iridis co-occurrent and due to type 1 diabetes mellitus (disorder) | ... |  | Y | **?****+****-** |
| Contract | · · **Disorder of nerve co-occurrent and due to type 1 diabetes mellitus (disorder)** | ... |  | Y | **?****+****-****++****--** |
| Contract | · · · **Autonomic neuropathy co-occurrent and due to type 1 diabetes mellitus (disorder)** | ... |  | Y | **?****+****-****++****--** |
|  | · · · · Gastroparesis co-occurrent and due to type 1 diabetes mellitus (disorder) | ... |  | Y | **?****+****-** |
|  | · · · Cranial nerve palsy co-occurrent and due to type 1 diabetes mellitus (disorder) | ... |  | Y | **?****+****-** |
| Contract | · · · **Peripheral neuropathy co-occurrent and due to type 1 diabetes mellitus (disorder)** | ... |  | Y | **?****+****-****++****--** |
|  | · · · · Lumbosacral radiculoplexus neuropathy co-occurrent and due to type 1 diabetes mellitus (disorder) | ... |  | Y | **?****+****-** |
|  | · · · · Mononeuropathy co-occurrent and due to type 1 diabetes mellitus (disorder) | ... |  | Y | **?****+****-** |
|  | · · · · Polyneuropathy co-occurrent and due to type 1 diabetes mellitus (disorder) | ... |  | Y | **?****+****-** |
|  | · · · · Sensory neuropathy due to type 1 diabetes mellitus (disorder) | ... |  | Y | **?****+****-** |
|  | · · Erectile dysfunction co-occurrent and due to type 1 diabetes mellitus (disorder) | ... |  | Y | **?****+****-** |
|  | · · Insulin dependent diabetes mellitus type IA (disorder) | ... |  | Y | **?****+****-** |
|  | · · Insulin dependent diabetes mellitus type IB (disorder) | ... |  | Y | **?****+****-** |
|  | · · Intellectual disability, craniofacial dysmorphism, hypogonadism, diabetes mellitus syndrome (disorder) | ... |  | Y | **?****+****-** |
|  | · · Latent autoimmune diabetes mellitus in adult (disorder) | ... |  | Y | **?****+****-** |
|  | · · Neurological disorder co-occurrent and due to type 1 diabetes mellitus (disorder) | ... |  | Y | **?****+****-** |
|  | · · Peripheral angiopathy due to type 1 diabetes mellitus (disorder) | ... |  | Y | **?****+****-** |
|  | · · Pre-existing type 1 diabetes mellitus (disorder) | ... |  | Y | **?****+****-** |
|  | · · Type 1 diabetes mellitus with hyperosmolar coma (disorder) | ... |  | Y | **?****+****-** |
|  | · · Type I diabetes mellitus maturity onset (disorder) | ... |  | Y | **?****+****-** |
|  | · · Type I diabetes mellitus with arthropathy (disorder) | ... |  | Y | **?****+****-** |
|  | · · Type I diabetes mellitus with hypoglycemic coma (disorder) | ... |  | Y | **?****+****-** |
|  | · · Type I diabetes mellitus with ulcer (disorder) | ... |  | Y | **?****+****-** |
|  | · · Type I diabetes mellitus without complication (disorder) | ... |  | Y | **?****+****-** |
| Contract | · · **Pregnancy and type 1 diabetes mellitus (disorder)** | ... |  | Y | **?****+****-****++****--** |
|  | · · · Pre-existing type 1 diabetes mellitus in pregnancy (disorder) | ... |  | Y | **?****+****-** |
|  | · · Type I diabetes mellitus in remission (disorder) | ... |  | Y | **?****+****-** |
| Contract | · **Diabetes mellitus type 2 (disorder)** | ... |  | Y | **?****+****-****++****--** |
|  | · · Blindness co-occurrent and due to type 2 diabetes mellitus (disorder) | ... |  | Y | **?****+****-** |
|  | · · Diabetes mellitus type 2 in nonobese (disorder) | ... |  | Y | **?****+****-** |
|  | · · Diabetes mellitus type 2 in obese (disorder) | ... |  | Y | **?****+****-** |
| Contract | · · **Disorder of eye co-occurrent and due to type 2 diabetes mellitus (disorder)** | ... |  | Y | **?****+****-****++****--** |
|  | · · · Glaucoma due to type 2 diabetes mellitus (disorder) | ... |  | Y | **?****+****-** |
|  | · · · Rubeosis iridis co-occurrent and due to type 2 diabetes mellitus (disorder) | ... |  | Y | **?****+****-** |
|  | · · Erectile dysfunction co-occurrent and due to type 2 diabetes mellitus (disorder) | ... |  | Y | **?****+****-** |
|  | · · Insulin treated type 2 diabetes mellitus (disorder) | ... |  | Y | **?****+****-** |
| Contract | · · **Neurological disorder co-occurrent and due to type 2 diabetes mellitus (disorder)** | ... |  | Y | **?****+****-****++****--** |
|  | · · · Hypoglycemic coma co-occurrent and due to diabetes mellitus type II (disorder) | ... |  | Y | **?****+****-** |
| Contract | · · · **Neuropathy due to type 2 diabetes mellitus (disorder)** | ... |  | Y | **?****+****-****++****--** |
| Contract | · · · · **Autonomic neuropathy co-occurrent and due to type 2 diabetes mellitus (disorder)** | ... |  | Y | **?****+****-****++****--** |
|  | · · · · · Gastroparesis co-occurrent and due to type 2 diabetes mellitus (disorder) | ... |  | Y | **?****+****-** |
|  | · · · · Cranial nerve palsy co-occurrent and due to type 2 diabetes mellitus (disorder) | ... |  | Y | **?****+****-** |
|  | · · Pre-existing type 2 diabetes mellitus (disorder) | ... |  | Y | **?****+****-** |
|  | · · Type 2 diabetes mellitus with acanthosis nigricans (disorder) | ... |  | Y | **?****+****-** |
|  | · · Type 2 diabetes mellitus with hyperosmolar coma (disorder) | ... |  | Y | **?****+****-** |
|  | · · Type 2 diabetes mellitus with peripheral angiopathy (disorder) | ... |  | Y | **?****+****-** |
| Contract | · · **Type II diabetes mellitus with arthropathy (disorder)** | ... |  | Y | **?****+****-****++****--** |
|  | · · · Type II diabetes mellitus with neuropathic arthropathy (disorder) | ... |  | Y | **?****+****-** |
|  | · · Type II diabetes mellitus with ulcer (disorder) | ... |  | Y | **?****+****-** |
| Contract | · · **Type II diabetes mellitus without complication (disorder)** | ... |  | Y | **?****+****-****++****--** |
|  | · · · Diabetes mellitus type 2 without retinopathy (disorder) | ... |  | Y | **?****+****-** |
| Contract | · · **Pregnancy and type 2 diabetes mellitus (disorder)** | ... |  | Y | **?****+****-****++****--** |
|  | · · · Pre-existing type 2 diabetes mellitus in pregnancy (disorder) | ... |  | Y | **?****+****-** |
|  | · · Type II diabetes mellitus in remission (disorder) | ... |  | Y | **?****+****-** |
|  | · Diabetes mellitus with multiple complications (disorder) | ... |  | Y | **?****+****-** |
| Contract | · **Diabetes mellitus without complication (disorder)** | ... |  | Y | **?****+****-****++****--** |
|  | · · Type I diabetes mellitus without complication (disorder) | ... |  | Y | **?****+****-** |
| Contract | · · **Type II diabetes mellitus without complication (disorder)** | ... |  | Y | **?****+****-****++****--** |
|  | · · · Diabetes mellitus type 2 without retinopathy (disorder) | ... |  | Y | **?****+****-** |
|  | · Diabetic mastopathy (disorder) | ... |  | Y | **?****+****-** |
| Contract | · **Disorder of kidney co-occurrent and due to diabetes mellitus (disorder)** | ... |  | Y | **?****+****-****++****--** |
|  | · · Armanni-Ebstein kidney (disorder) | ... |  | Y | **?****+****-** |
| Contract | · · **Diabetic glomerulosclerosis (disorder)** | ... |  | Y | **?****+****-****++****--** |
|  | · · · Nodular type diabetic glomerulosclerosis (disorder) | ... |  | Y | **?****+****-** |
|  | · · Kimmelstiel-Wilson syndrome (disorder) | ... |  | Y | **?****+****-** |
|  | · · Macroalbuminuric diabetic nephropathy (disorder) | ... |  | Y | **?****+****-** |
| Contract | · · **Nephrotic syndrome due to diabetes mellitus (disorder)** | ... |  | Y | **?****+****-****++****--** |
|  | · · · Nephrotic syndrome due to type 2 diabetes mellitus (disorder) | ... |  | Y | **?****+****-** |
| Contract | · · **Renal disorder associated with type I diabetes mellitus (disorder)** | ... |  | Y | **?****+****-****++****--** |
|  | · · · Type 1 diabetes mellitus with persistent microalbuminuria (disorder) | ... |  | Y | **?****+****-** |
|  | · · Renal disorder due to type 2 diabetes mellitus (disorder) | ... |  | Y | **?****+****-** |
|  | · · Renal papillary necrosis due to diabetes mellitus (disorder) | ... |  | Y | **?****+****-** |
| Contract | · **Disorder of nervous system co-occurrent and due to diabetes mellitus (disorder)** | ... |  | Y | **?****+****-****++****--** |
| Contract | · · **Coma associated with diabetes mellitus (disorder)** | ... |  | Y | **?****+****-****++****--** |
|  | · · · Coma associated with malnutrition-related diabetes mellitus (disorder) | ... |  | Y | **?****+****-** |
|  | · · · Diabetic coma with ketoacidosis (disorder) | ... |  | Y | **?****+****-** |
|  | · · · Non-ketotic non-hyperosmolar coma associated with diabetes mellitus (disorder) | ... |  | Y | **?****+****-** |
|  | · · Neurological disorder associated with malnutrition-related diabetes mellitus (disorder) | ... |  | Y | **?****+****-** |
| Contract | · · **Neuropathy co-occurrent and due to diabetes mellitus (disorder)** | ... |  | Y | **?****+****-****++****--** |
|  | · · · Acute painful diabetic neuropathy (disorder) | ... |  | Y | **?****+****-** |
|  | · · · Asymptomatic neuropathy co-occurrent and due to diabetes mellitus (disorder) | ... |  | Y | **?****+****-** |
| Contract | · · · **Autonomic neuropathy co-occurrent and due to diabetes mellitus (disorder)** | ... |  | Y | **?****+****-****++****--** |
|  | · · · · Diarrhea co-occurrent and due to diabetes mellitus (disorder) | ... |  | Y | **?****+****-** |
|  | · · · · Gastroparesis co-occurrent and due to diabetes mellitus (disorder) | ... |  | Y | **?****+****-** |
| Contract | · · · **Chronic painful diabetic neuropathy (disorder)** | ... |  | Y | **?****+****-****++****--** |
|  | · · · · Chronic painful polyneuropathy co-occurrent and due to diabetes mellitus (disorder) | ... |  | Y | **?****+****-** |
|  | · · · Cranial nerve palsy co-occurrent and due to diabetes mellitus (disorder) | ... |  | Y | **?****+****-** |
|  | · · · Diabetic mononeuropathy multiplex (disorder) | ... |  | Y | **?****+****-** |
|  | · · · Diabetic neuropathy with neurologic complication (disorder) | ... |  | Y | **?****+****-** |
| Contract | · **Disorder of soft tissue co-occurrent and due to diabetes mellitus (disorder)** | ... |  | Y | **?****+****-****++****--** |
|  | · · Bullosis diabeticorum (disorder) | ... |  | Y | **?****+****-** |
|  | · · Diabetic intraretinal microvascular anomaly (disorder) | ... |  | Y | **?****+****-** |
| Contract | · · **Ischemic maculopathy co-occurrent and due to diabetes mellitus (disorder)** | ... |  | Y | **?****+****-****++****--** |
|  | · · · Mixed maculopathy co-occurrent and due to diabetes mellitus (disorder) | ... |  | Y | **?****+****-** |
| Contract | · · **Peripheral neuropathy co-occurrent and due to diabetes mellitus (disorder)** | ... |  | Y | **?****+****-****++****--** |
|  | · · · Asymmetric proximal motor neuropathy co-occurrent and due to diabetes mellitus (disorder) | ... |  | Y | **?****+****-** |
| Contract | · · · **Mononeuropathy co-occurrent and due to diabetes mellitus (disorder)** | ... |  | Y | **?****+****-****++****--** |
|  | · · · · Femoral mononeuropathy co-occurrent and due to diabetes mellitus (disorder) | ... |  | Y | **?****+****-** |
|  | · · · · Mononeuritis multiplex co-occurrent and due to diabetes mellitus (disorder) | ... |  | Y | **?****+****-** |
|  | · · · · Mononeuropathy co-occurrent and due to type 2 diabetes mellitus (disorder) | ... |  | Y | **?****+****-** |
|  | · · · · Mononeuropathy simplex co-occurrent and due to diabetes mellitus (disorder) | ... |  | Y | **?****+****-** |
|  | · · · Ophthalmoplegia co-occurrent and due to diabetes mellitus (disorder) | ... |  | Y | **?****+****-** |
| Contract | · · · **Peripheral neuropathy co-occurrent and due to type 2 diabetes mellitus (disorder)** | ... |  | Y | **?****+****-****++****--** |
|  | · · · · Lumbosacral radiculoplexus neuropathy co-occurrent and due to type 2 diabetes mellitus (disorder) | ... |  | Y | **?****+****-** |
|  | · · · · Peripheral sensory neuropathy due to type 2 diabetes mellitus (disorder) | ... |  | Y | **?****+****-** |
|  | · · · · Polyneuropathy co-occurrent and due to type 2 diabetes mellitus (disorder) | ... |  | Y | **?****+****-** |
| Contract | · · · **Polyneuropathy co-occurrent and due to diabetes mellitus (disorder)** | ... |  | Y | **?****+****-****++****--** |
|  | · · · · Asymmetric polyneuropathy co-occurrent and due to diabetes mellitus (disorder) | ... |  | Y | **?****+****-** |
| Contract | · · · · **Motor polyneuropathy co-occurrent and due to diabetes mellitus (disorder)** | ... |  | Y | **?****+****-****++****--** |
|  | · · · · · Mixed sensorimotor polyneuropathy co-occurrent and due to diabetes mellitus (disorder) | ... |  | Y | **?****+****-** |
|  | · · · · Sensory neuropathy co-occurrent and due to diabetes mellitus (disorder) | ... |  | Y | **?****+****-** |
|  | · · · Pseudotabes co-occurrent and due to diabetes mellitus (disorder) | ... |  | Y | **?****+****-** |
| Contract | · · · **Radiculoplexus neuropathy co-occurrent and due to diabetes mellitus (disorder)** | ... |  | Y | **?****+****-****++****--** |
|  | · · · · Cervical radiculoplexus neuropathy co-occurrent and due to diabetes mellitus (disorder) | ... |  | Y | **?****+****-** |
|  | · · · · Lumbosacral radiculoplexus neuropathy co-occurrent and due to diabetes mellitus (disorder) | ... |  | Y | **?****+****-** |
|  | · · · · Thoracic radiculopathy co-occurrent and due to diabetes mellitus (disorder) | ... |  | Y | **?****+****-** |
|  | · · · Symmetric proximal motor neuropathy co-occurrent and due to diabetes mellitus (disorder) | ... |  | Y | **?****+****-** |
|  | · · · Symptomatic diabetic peripheral neuropathy (disorder) | ... |  | Y | **?****+****-** |
| Contract | · · **Retinal microaneurysm co-occurrent and due to diabetes mellitus (disorder)** | ... |  | Y | **?****+****-****++****--** |
|  | · · · Retinal microaneurysm of left eye co-occurrent and due to diabetes mellitus (disorder) | ... |  | Y | **?****+****-** |
|  | · · · Retinal microaneurysm of right eye co-occurrent and due to diabetes mellitus (disorder) | ... |  | Y | **?****+****-** |
| Contract | · · **Retinal venous beading co-occurrent and due to diabetes mellitus (disorder)** | ... |  | Y | **?****+****-****++****--** |
|  | · · · Retinal venous beading of left eye co-occurrent and due to diabetes mellitus (disorder) | ... |  | Y | **?****+****-** |
|  | · · · Retinal venous beading of right eye co-occurrent and due to diabetes mellitus (disorder) | ... |  | Y | **?****+****-** |
|  | · Erectile dysfunction co-occurrent and due to diabetes mellitus (disorder) | ... |  | Y | **?****+****-** |
| Contract | · **Gingival disease co-occurrent with diabetes mellitus (disorder)** | ... |  | Y | **?****+****-****++****--** |
|  | · · Gingivitis co-occurrent with diabetes mellitus (disorder) | ... |  | Y | **?****+****-** |
|  | · Houssay's syndrome (disorder) | ... |  | Y | **?****+****-** |
|  | · Ketosis-prone diabetes mellitus (disorder) | ... |  | Y | **?****+****-** |
|  | · Lactic acidosis co-occurrent and due to diabetes mellitus (disorder) | ... |  | Y | **?****+****-** |
|  | · Maternally inherited diabetes mellitus (disorder) | ... |  | Y | **?****+****-** |
|  | · Metabolic acidosis co-occurrent and due to diabetes mellitus (disorder) | ... |  | Y | **?****+****-** |
|  | · Neovascular glaucoma co-occurrent and due to diabetes mellitus (disorder) | ... |  | Y | **?****+****-** |
|  | · Pancreatic hypoplasia, diabetes mellitus, congenital heart disease syndrome (disorder) | ... |  | Y | **?****+****-** |
| Contract | · **Peripheral vascular disorder co-occurrent and due to diabetes mellitus (disorder)** | ... |  | Y | **?****+****-****++****--** |
|  | · · Peripheral circulatory disorder associated with type I diabetes mellitus (disorder) | ... |  | Y | **?****+****-** |
|  | · · Peripheral circulatory disorder associated with type II diabetes mellitus (disorder) | ... |  | Y | **?****+****-** |
|  | · Pre-existing diabetes mellitus (disorder) | ... |  | Y | **?****+****-** |
| Contract | · **Retinopathy co-occurrent and due to diabetes mellitus (disorder)** | ... |  | Y | **?****+****-****++****--** |
|  | · · Advanced retinal disease co-occurrent and due to diabetes mellitus (disorder) | ... |  | Y | **?****+****-** |
|  | · · Diabetic retinopathy detected by national screening programme (disorder) | ... |  | Y | **?****+****-** |
| Contract | · · **Disorder of macula co-occurrent and due to diabetes mellitus (disorder)** | ... |  | Y | **?****+****-****++****--** |
|  | · · · Advanced maculopathy co-occurrent and due to diabetes mellitus (disorder) | ... |  | Y | **?****+****-** |
| Contract | · · · **Clinically significant macular edema co-occurrent and due to diabetes mellitus (disorder)** | ... |  | Y | **?****+****-****++****--** |
|  | · · · · Clinically significant macular edema of left eye co-occurrent and due to diabetes mellitus (disorder) | ... |  | Y | **?****+****-** |
|  | · · · · Clinically significant macular edema of right eye co-occurrent and due to diabetes mellitus (disorder) | ... |  | Y | **?****+****-** |
|  | · · · · Severe nonproliferative retinopathy with clinically significant macular edema co-occurrent and due to diabetes mellitus (disorder) | ... |  | Y | **?****+****-** |
|  | · · · · Very severe nonproliferative retinopathy with clinically significant macular edema co-occurrent and due to diabetes mellitus (disorder) | ... |  | Y | **?****+****-** |
|  | · · · Diffuse exudative maculopathy co-occurrent and due to diabetes mellitus (disorder) | ... |  | Y | **?****+****-** |
|  | · · · Disorder of left macula co-occurrent and due to diabetes mellitus (disorder) | ... |  | Y | **?****+****-** |
|  | · · · Disorder of right macula co-occurrent and due to diabetes mellitus (disorder) | ... |  | Y | **?****+****-** |
|  | · · · Exudative maculopathy co-occurrent and due to type 1 diabetes mellitus (disorder) | ... |  | Y | **?****+****-** |
|  | · · · Exudative maculopathy co-occurrent and due to type 2 diabetes mellitus (disorder) | ... |  | Y | **?****+****-** |
|  | · · · Focal exudative maculopathy co-occurrent and due to diabetes mellitus (disorder) | ... |  | Y | **?****+****-** |
|  | · · · Macular edema and retinopathy due to type 2 diabetes mellitus (disorder) | ... |  | Y | **?****+****-** |
|  | · · · Macular edema not clinically significant co-occurrent and due to diabetes mellitus (disorder) | ... |  | Y | **?****+****-** |
|  | · · · Non-high-risk proliferative diabetic retinopathy with clinically significant macular edema (disorder) | ... |  | Y | **?****+****-** |
|  | · · · Proliferative diabetic retinopathy - high risk with clinically significant macular edema (disorder) | ... |  | Y | **?****+****-** |
| Contract | · · **Nonproliferative retinopathy co-occurrent and due to diabetes mellitus (disorder)** | ... |  | Y | **?****+****-****++****--** |
| Contract | · · · **Mild nonproliferative retinopathy co-occurrent and due to diabetes mellitus (disorder)** | ... |  | Y | **?****+****-****++****--** |
|  | · · · · Mild nonproliferative retinopathy co-occurrent and due to type 1 diabetes mellitus (disorder) | ... |  | Y | **?****+****-** |
|  | · · · · Mild nonproliferative retinopathy co-occurrent and due to type 2 diabetes mellitus (disorder) | ... |  | Y | **?****+****-** |
|  | · · · · Mild nonproliferative retinopathy of left eye co-occurrent and due to diabetes mellitus (disorder) | ... |  | Y | **?****+****-** |
|  | · · · · Mild nonproliferative retinopathy of right eye co-occurrent and due to diabetes mellitus (disorder) | ... |  | Y | **?****+****-** |
| Contract | · · · **Moderate nonproliferative retinopathy co-occurrent and due to diabetes mellitus (disorder)** | ... |  | Y | **?****+****-****++****--** |
|  | · · · · Moderate nonproliferative retinopathy co-occurrent and due to type 1 diabetes mellitus (disorder) | ... |  | Y | **?****+****-** |
|  | · · · · Moderate nonproliferative retinopathy co-occurrent and due to type 2 diabetes mellitus (disorder) | ... |  | Y | **?****+****-** |
|  | · · · · Moderate nonproliferative retinopathy of left eye co-occurrent and due to diabetes mellitus (disorder) | ... |  | Y | **?****+****-** |
|  | · · · · Moderate nonproliferative retinopathy of right eye co-occurrent and due to diabetes mellitus (disorder) | ... |  | Y | **?****+****-** |
|  | · · · Nonproliferative retinopathy co-occurrent and due to type 1 diabetes mellitus (disorder) | ... |  | Y | **?****+****-** |
|  | · · · Nonproliferative retinopathy co-occurrent and due to type 2 diabetes mellitus (disorder) | ... |  | Y | **?****+****-** |
| Contract | · · · **Preproliferative retinopathy co-occurrent and due to diabetes mellitus (disorder)** | ... |  | Y | **?****+****-****++****--** |
|  | · · · · On examination - left eye preproliferative diabetic retinopathy (disorder) | ... |  | Y | **?****+****-** |
|  | · · · · On examination - right eye preproliferative diabetic retinopathy (disorder) | ... |  | Y | **?****+****-** |
|  | · · · · Preproliferative retinopathy of left eye co-occurrent and due to diabetes mellitus (disorder) | ... |  | Y | **?****+****-** |
|  | · · · · Preproliferative retinopathy of right eye co-occurrent and due to diabetes mellitus (disorder) | ... |  | Y | **?****+****-** |
| Contract | · · · **Severe nonproliferative retinopathy co-occurrent and due to diabetes mellitus (disorder)** | ... |  | Y | **?****+****-****++****--** |
|  | · · · · Severe nonproliferative retinopathy of left eye co-occurrent and due to diabetes mellitus (disorder) | ... |  | Y | **?****+****-** |
|  | · · · · Severe nonproliferative retinopathy of right eye co-occurrent and due to diabetes mellitus (disorder) | ... |  | Y | **?****+****-** |
|  | · · · · Severe nonproliferative retinopathy without macular edema co-occurrent and due to diabetes mellitus (disorder) | ... |  | Y | **?****+****-** |
| Contract | · · · **Very severe nonproliferative retinopathy co-occurrent and due to diabetes mellitus (disorder)** | ... |  | Y | **?****+****-****++****--** |
|  | · · · · Very severe nonproliferative retinopathy of left eye co-occurrent and due to diabetes mellitus (disorder) | ... |  | Y | **?****+****-** |
|  | · · · · Very severe nonproliferative retinopathy of right eye co-occurrent and due to diabetes mellitus (disorder) | ... |  | Y | **?****+****-** |
|  | · · · · Very severe nonproliferative retinopathy without macular edema co-occurrent and due to diabetes mellitus (disorder) | ... |  | Y | **?****+****-** |
|  | · · On examination - left eye background diabetic retinopathy (disorder) | ... |  | Y | **?****+****-** |
|  | · · On examination - right eye background diabetic retinopathy (disorder) | ... |  | Y | **?****+****-** |
|  | · · On examination - sight threatening diabetic retinopathy (disorder) | ... |  | Y | **?****+****-** |
| Contract | · · **Proliferative retinopathy co-occurrent and due to diabetes mellitus (disorder)** | ... |  | Y | **?****+****-****++****--** |
|  | · · · Non-high-risk proliferative diabetic retinopathy with no macular edema (disorder) | ... |  | Y | **?****+****-** |
| Contract | · · · **On examination - left eye proliferative diabetic retinopathy (disorder)** | ... |  | Y | **?****+****-****++****--** |
|  | · · · · On examination - left eye stable treated proliferative diabetic retinopathy (disorder) | ... |  | Y | **?****+****-** |
| Contract | · · · **On examination - right eye proliferative diabetic retinopathy (disorder)** | ... |  | Y | **?****+****-****++****--** |
|  | · · · · On examination - right eye stable treated proliferative diabetic retinopathy (disorder) | ... |  | Y | **?****+****-** |
| Contract | · · · **Proliferative diabetic retinopathy - high risk (disorder)** | ... |  | Y | **?****+****-****++****--** |
|  | · · · · High risk proliferative diabetic retinopathy not amenable to photocoagulation (disorder) | ... |  | Y | **?****+****-** |
|  | · · · · Proliferative diabetic retinopathy - high risk with no macular edema (disorder) | ... |  | Y | **?****+****-** |
|  | · · · · Very severe proliferative diabetic retinopathy (disorder) | ... |  | Y | **?****+****-** |
|  | · · · Proliferative diabetic retinopathy - iris neovascularization (disorder) | ... |  | Y | **?****+****-** |
| Contract | · · · **Proliferative diabetic retinopathy - non high risk (disorder)** | ... |  | Y | **?****+****-****++****--** |
|  | · · · · Proliferative diabetic retinopathy - quiescent (disorder) | ... |  | Y | **?****+****-** |
|  | · · · Proliferative diabetic retinopathy following surgery (disorder) | ... |  | Y | **?****+****-** |
|  | · · · Proliferative diabetic retinopathy with new vessels elsewhere than on disc (disorder) | ... |  | Y | **?****+****-** |
|  | · · · Proliferative diabetic retinopathy with new vessels on disc (disorder) | ... |  | Y | **?****+****-** |
|  | · · · Proliferative retinopathy co-occurrent and due to type 1 diabetes mellitus (disorder) | ... |  | Y | **?****+****-** |
| Contract | · · · **Proliferative retinopathy co-occurrent and due to type 2 diabetes mellitus (disorder)** | ... |  | Y | **?****+****-****++****--** |
|  | · · · · Proliferative retinopathy with retinal edema co-occurrent and due to type 2 diabetes mellitus (disorder) | ... |  | Y | **?****+****-** |
|  | · · · Proliferative retinopathy of left eye co-occurrent and due to diabetes mellitus (disorder) | ... |  | Y | **?****+****-** |
|  | · · · Proliferative retinopathy of right eye co-occurrent and due to diabetes mellitus (disorder) | ... |  | Y | **?****+****-** |
| Contract | · · **Retinal edema co-occurrent and due to diabetes mellitus (disorder)** | ... |  | Y | **?****+****-****++****--** |
|  | · · · Retinal edema co-occurrent and due to type 1 diabetes mellitus (disorder) | ... |  | Y | **?****+****-** |
|  | · · · Retinal edema co-occurrent and due to type 2 diabetes mellitus (disorder) | ... |  | Y | **?****+****-** |
| Contract | · · **Retinal ischemia co-occurrent and due to diabetes mellitus (disorder)** | ... |  | Y | **?****+****-****++****--** |
|  | · · · Retinal ischemia co-occurrent and due to type 1 diabetes mellitus (disorder) | ... |  | Y | **?****+****-** |
|  | · · · Retinal ischemia co-occurrent and due to type 2 diabetes mellitus (disorder) | ... |  | Y | **?****+****-** |
| Contract | · · **Retinopathy co-occurrent and due to type 1 diabetes mellitus (disorder)** | ... |  | Y | **?****+****-****++****--** |
|  | · · · Traction retinal detachment co-occurrent and due to type 1 diabetes mellitus (disorder) | ... |  | Y | **?****+****-** |
| Contract | · · **Retinopathy co-occurrent and due to type 2 diabetes mellitus (disorder)** | ... |  | Y | **?****+****-****++****--** |
|  | · · · Traction retinal detachment co-occurrent and due to type 2 diabetes mellitus (disorder) | ... |  | Y | **?****+****-** |
|  | · · Traction retinal detachment co-occurrent and due to diabetes mellitus (disorder) | ... |  | Y | **?****+****-** |
|  | · · Visually threatening diabetic retinopathy (disorder) | ... |  | Y | **?****+****-** |
| Contract | · **Secondary diabetes mellitus (disorder)** | ... |  | Y | **?****+****-****++****--** |
| Contract | · · **Diabetes mellitus associated with genetic syndrome (disorder)** | ... |  | Y | **?****+****-****++****--** |
|  | · · · Atherosclerosis, deafness, diabetes, epilepsy, nephropathy syndrome (disorder) | ... |  | Y | **?****+****-** |
|  | · · · Diabetes mellitus AND insipidus with optic atrophy AND deafness (disorder) | ... |  | Y | **?****+****-** |
|  | · · · Diabetes-deafness syndrome maternally transmitted (disorder) | ... |  | Y | **?****+****-** |
|  | · · · Hyperproinsulinemia (disorder) | ... |  | Y | **?****+****-** |
|  | · · · Insulin-dependent diabetes mellitus secretory diarrhea syndrome (disorder) | ... |  | Y | **?****+****-** |
|  | · · · Myopathy and diabetes mellitus (disorder) | ... |  | Y | **?****+****-** |
|  | · · · Primary microcephaly, mild intellectual disability, young-onset diabetes syndrome (disorder) | ... |  | Y | **?****+****-** |
|  | · · · Wolfram-like syndrome (disorder) | ... |  | Y | **?****+****-** |
|  | · · · Pineal hyperplasia AND diabetes mellitus syndrome (disorder) | ... |  | Y | **?****+****-** |
|  | · · Diabetes mellitus associated with hormonal etiology (disorder) | ... |  | Y | **?****+****-** |
| Contract | · · **Diabetes mellitus associated with pancreatic disease (disorder)** | ... |  | Y | **?****+****-****++****--** |
|  | · · · Diabetes mellitus due to cystic fibrosis (disorder) | ... |  | Y | **?****+****-** |
|  | · · · Diabetes mellitus due to structurally abnormal insulin (disorder) | ... |  | Y | **?****+****-** |
| Contract | · · · **Fibrocalculous pancreatic diabetes (disorder)** | ... |  | Y | **?****+****-****++****--** |
|  | · · · · Malnutrition-related diabetes mellitus - fibrocalculous (disorder) | ... |  | Y | **?****+****-** |
|  | · · Diabetes mellitus associated with receptor abnormality (disorder) | ... |  | Y | **?****+****-** |
| Contract | · · **Diabetes mellitus caused by chemical (disorder)** | ... |  | Y | **?****+****-****++****--** |
| Contract | · · · **Steroid-induced diabetes (disorder)** | ... |  | Y | **?****+****-****++****--** |
|  | · · · · Steroid-induced diabetes mellitus without complication (disorder) | ... |  | Y | **?****+****-** |
|  | · · · Diabetes mellitus caused by insulin receptor antibodies (disorder) | ... |  | Y | **?****+****-** |
|  | · · Diabetes mellitus co-occurrent and due to cystic fibrosis (disorder) | ... |  | Y | **?****+****-** |
|  | · · Diabetes mellitus in neonate small for gestational age (disorder) | ... |  | Y | **?****+****-** |
| Contract | · · **Drug-induced diabetes mellitus (disorder)** | ... |  | Y | **?****+****-****++****--** |
| Contract | · · · **Diabetes mellitus caused by non-steroid drugs (disorder)** | ... |  | Y | **?****+****-****++****--** |
|  | · · · · Diabetes mellitus caused by non-steroid drugs without complication (disorder) | ... |  | Y | **?****+****-** |
| Contract | · · · **Steroid-induced diabetes (disorder)** | ... |  | Y | **?****+****-****++****--** |
|  | · · · · Steroid-induced diabetes mellitus without complication (disorder) | ... |  | Y | **?****+****-** |
|  | · · · Hyperosmolarity co-occurrent and due to drug induced diabetes mellitus (disorder) | ... |  | Y | **?****+****-** |
|  | · · Insulin autoimmune syndrome (disorder) | ... |  | Y | **?****+****-** |
| Contract | · · **Malnutrition related diabetes mellitus (disorder)** | ... |  | Y | **?****+****-****++****--** |
|  | · · · Malnutrition-related diabetes mellitus - fibrocalculous (disorder) | ... |  | Y | **?****+****-** |
|  | · · · Malnutrition-related diabetes mellitus with ketoacidosis (disorder) | ... |  | Y | **?****+****-** |
|  | · · · Malnutrition-related diabetes mellitus with peripheral circulatory complications (disorder) | ... |  | Y | **?****+****-** |
|  | · · · Malnutrition-related diabetes mellitus with renal complications (disorder) | ... |  | Y | **?****+****-** |
|  | · · · Malnutrition-related diabetes mellitus without complications (disorder) | ... |  | Y | **?****+****-** |
|  | · · · Pre-existing malnutrition-related diabetes mellitus (disorder) | ... |  | Y | **?****+****-** |
|  | · · · Protein-deficient diabetes mellitus (disorder) | ... |  | Y | **?****+****-** |
|  | · · · Malnutrition-related diabetes mellitus with multiple complications (disorder) | ... |  | Y | **?****+****-** |
| Contract | · · **Nonproliferative retinopathy co-occurrent and due to secondary diabetes mellitus (disorder)** | ... |  | Y | **?****+****-****++****--** |
|  | · · · Mild nonproliferative retinopathy co-occurrent and due to secondary diabetes mellitus (disorder) | ... |  | Y | **?****+****-** |
|  | · · · Moderate nonproliferative retinopathy co-occurrent and due to secondary diabetes mellitus (disorder) | ... |  | Y | **?****+****-** |
|  | · · Posttransplant diabetes mellitus (disorder) | ... |  | Y | **?****+****-** |
|  | · · Secondary endocrine diabetes mellitus (disorder) | ... |  | Y | **?****+****-** |
| Contract | **Diabetic complication (disorder)** | ... |  | Y | **?****+****-****++****--** |
| Contract | · **Abnormal metabolic state in diabetes mellitus (disorder)** | ... |  | Y | **?****+****-****++****--** |
| Contract | · · **Acidosis due to type 1 diabetes mellitus (disorder)** | ... |  | Y | **?****+****-****++****--** |
|  | · · · Ketoacidosis in type I diabetes mellitus (disorder) | ... |  | Y | **?****+****-** |
| Contract | · · **Acidosis due to type 2 diabetes mellitus (disorder)** | ... |  | Y | **?****+****-****++****--** |
|  | · · · Ketoacidosis in type II diabetes mellitus (disorder) | ... |  | Y | **?****+****-** |
| Contract | · · **Diabetic severe hyperglycemia (disorder)** | ... |  | Y | **?****+****-****++****--** |
| Contract | · · · **Hyperglycemic crisis in diabetes mellitus (disorder)** | ... |  | Y | **?****+****-****++****--** |
| Contract | · · · · **Diabetic hyperosmolar non-ketotic state (disorder)** | ... |  | Y | **?****+****-****++****--** |
|  | · · · · · Hyperosmolar non-ketotic state in type 2 diabetes mellitus (disorder) | ... |  | Y | **?****+****-** |
|  | · · · · · Hyperosmolar hyperglycemic coma due to diabetes mellitus without ketoacidosis (disorder) | ... |  | Y | **?****+****-** |
| Contract | · · · · **Ketoacidosis in diabetes mellitus (disorder)** | ... |  | Y | **?****+****-****++****--** |
|  | · · · · · Diabetic ketoacidosis without coma (disorder) | ... |  | Y | **?****+****-** |
|  | · · · · · Ketoacidosis in type I diabetes mellitus (disorder) | ... |  | Y | **?****+****-** |
|  | · · · · · Ketoacidosis in type II diabetes mellitus (disorder) | ... |  | Y | **?****+****-** |
|  | · · · · · Ketoacidosis in type I diabetes mellitus (disorder) | ... |  | Y | **?****+****-** |
|  | · · · · · Ketoacidosis in type II diabetes mellitus (disorder) | ... |  | Y | **?****+****-** |
|  | · · Dyslipidemia due to type 1 diabetes mellitus (disorder) | ... |  | Y | **?****+****-** |
|  | · · Hyperglycemia due to type 1 diabetes mellitus (disorder) | ... |  | Y | **?****+****-** |
|  | · · Hyperglycemia due to type 2 diabetes mellitus (disorder) | ... |  | Y | **?****+****-** |
|  | · · Hyperlipidemia due to type 1 diabetes mellitus (disorder) | ... |  | Y | **?****+****-** |
|  | · · Hyperlipidemia due to type 2 diabetes mellitus (disorder) | ... |  | Y | **?****+****-** |
|  | · · Hyperosmolality due to uncontrolled type 1 diabetes mellitus (disorder) | ... |  | Y | **?****+****-** |
|  | · · Hyperosmolarity co-occurrent and due to drug induced diabetes mellitus (disorder) | ... |  | Y | **?****+****-** |
|  | · · Hypoglycemic coma in diabetes mellitus (disorder) | ... |  | Y | **?****+****-** |
|  | · · Malnutrition-related diabetes mellitus with multiple complications (disorder) | ... |  | Y | **?****+****-** |
|  | · · Mixed hyperlipidemia due to type 1 diabetes mellitus (disorder) | ... |  | Y | **?****+****-** |
|  | · · Mixed hyperlipidemia due to type II diabetes mellitus (disorder) | ... |  | Y | **?****+****-** |
|  | · · Lactic acidosis co-occurrent and due to diabetes mellitus (disorder) | ... |  | Y | **?****+****-** |
|  | · · Metabolic acidosis co-occurrent and due to diabetes mellitus (disorder) | ... |  | Y | **?****+****-** |
|  | · Absence of lower limb due to diabetes mellitus (disorder) | ... |  | Y | **?****+****-** |
|  | · Anemia of diabetes (disorder) | ... |  | Y | **?****+****-** |
|  | · Dermatitis due to drug induced diabetes mellitus (disorder) | ... |  | Y | **?****+****-** |
|  | · Diabetes mellitus caused by insulin receptor antibodies (disorder) | ... |  | Y | **?****+****-** |
| Contract | · **Diabetic foot (disorder)** | ... |  | Y | **?****+****-****++****--** |
|  | · · On examination - Left diabetic foot at risk (disorder) | ... |  | Y | **?****+****-** |
|  | · · On examination - Right diabetic foot at risk (disorder) | ... |  | Y | **?****+****-** |
| Contract | · **Diabetic foot ulcer (disorder)** | ... |  | Y | **?****+****-****++****--** |
| Contract | · · **Foot ulcer due to type 1 diabetes mellitus (disorder)** | ... |  | Y | **?****+****-****++****--** |
|  | · · · Forefoot ulcer due to type 1 diabetes mellitus (disorder) | ... |  | Y | **?****+****-** |
|  | · · · Heel AND/OR midfoot ulcer due to type 1 diabetes mellitus (disorder) | ... |  | Y | **?****+****-** |
|  | · · · Skin ulcer of toe due to diabetes mellitus type 1 (disorder) | ... |  | Y | **?****+****-** |
| Contract | · · **Foot ulcer due to type 2 diabetes mellitus (disorder)** | ... |  | Y | **?****+****-****++****--** |
|  | · · · Forefoot ulcer due to type 2 diabetes mellitus (disorder) | ... |  | Y | **?****+****-** |
|  | · · · Heel AND/OR midfoot ulcer due to type 2 diabetes mellitus (disorder) | ... |  | Y | **?****+****-** |
|  | · · · Ischemic foot ulcer due to type 2 diabetes mellitus (disorder) | ... |  | Y | **?****+****-** |
| Contract | · · · **Neuropathic foot ulcer due to type 2 diabetes mellitus (disorder)** | ... |  | Y | **?****+****-****++****--** |
|  | · · · · Neuropathic toe ulcer due to type 2 diabetes mellitus (disorder) | ... |  | Y | **?****+****-** |
|  | · · · Ulcer of left foot co-occurrent and due to diabetes mellitus type 2 (disorder) | ... |  | Y | **?****+****-** |
|  | · · · Ulcer of right foot co-occurrent and due to diabetes mellitus type 2 (disorder) | ... |  | Y | **?****+****-** |
|  | · · · Ulcer of toe due to type 2 diabetes mellitus (disorder) | ... |  | Y | **?****+****-** |
|  | · · Ischemic ulcer diabetic foot (disorder) | ... |  | Y | **?****+****-** |
| Contract | · · **Neuropathic ulcer of foot due to diabetes mellitus (disorder)** | ... |  | Y | **?****+****-****++****--** |
|  | · · · Neuropathic ulcer of midfoot AND/OR heel due to type 2 diabetes mellitus (disorder) | ... |  | Y | **?****+****-** |
| Contract | · **Diabetic hand syndrome (disorder)** | ... |  | Y | **?****+****-****++****--** |
|  | · · Cheiropathy due to type 2 diabetes mellitus (disorder) | ... |  | Y | **?****+****-** |
|  | · Diabetic optic papillopathy (disorder) | ... |  | Y | **?****+****-** |
| Contract | · **Disorder due to type 1 diabetes mellitus (disorder)** | ... |  | Y | **?****+****-****++****--** |
|  | · · Ankle ulcer due to type 1 diabetes mellitus (disorder) | ... |  | Y | **?****+****-** |
|  | · · Cataract of eye due to diabetes mellitus type 1 (disorder) | ... |  | Y | **?****+****-** |
| Contract | · · **Chronic kidney disease due to type 1 diabetes mellitus (disorder)** | ... |  | Y | **?****+****-****++****--** |
|  | · · · Chronic kidney disease stage 1 due to type 1 diabetes mellitus (disorder) | ... |  | Y | **?****+****-** |
|  | · · · Chronic kidney disease stage 2 due to type 1 diabetes mellitus (disorder) | ... |  | Y | **?****+****-** |
|  | · · · Chronic kidney disease stage 3 due to type 1 diabetes mellitus (disorder) | ... |  | Y | **?****+****-** |
|  | · · · Chronic kidney disease stage 4 due to type 1 diabetes mellitus (disorder) | ... |  | Y | **?****+****-** |
|  | · · · Chronic kidney disease stage 5 due to type 1 diabetes mellitus (disorder) | ... |  | Y | **?****+****-** |
|  | · · · Hypertension in chronic kidney disease due to type 1 diabetes mellitus (disorder) | ... |  | Y | **?****+****-** |
|  | · · Chronic ulcer of skin due to type 1 diabetes mellitus (disorder) | ... |  | Y | **?****+****-** |
|  | · · Diabetic dermopathy due to type 1 diabetes mellitus (disorder) | ... |  | Y | **?****+****-** |
|  | · · Diabetic embryopathy (disorder) | ... |  | Y | **?****+****-** |
|  | · · Diabetic neuropathic arthropathy due to type 1 diabetes mellitus (disorder) | ... |  | Y | **?****+****-** |
| Contract | · · **End stage renal disease on dialysis due to type 1 diabetes mellitus (disorder)** | ... |  | Y | **?****+****-****++****--** |
|  | · · · Hypertension concurrent and due to end stage renal disease on dialysis due to type 1 diabetes mellitus (disorder) | ... |  | Y | **?****+****-** |
| Contract | · · **Hypoglycemia due to type 1 diabetes mellitus (disorder)** | ... |  | Y | **?****+****-****++****--** |
|  | · · · Hypoglycemic unawareness in type 1 diabetes mellitus (disorder) | ... |  | Y | **?****+****-** |
|  | · · Hypoglycemic coma in type I diabetes mellitus (disorder) | ... |  | Y | **?****+****-** |
|  | · · Ketoacidotic coma in type I diabetes mellitus (disorder) | ... |  | Y | **?****+****-** |
|  | · · Macular edema due to type 1 diabetes mellitus (disorder) | ... |  | Y | **?****+****-** |
|  | · · Multiple complications of type I diabetes mellitus (disorder) | ... |  | Y | **?****+****-** |
|  | · · Nephrotic syndrome due to type 1 diabetes mellitus (disorder) | ... |  | Y | **?****+****-** |
|  | · · Osteomyelitis due to type 1 diabetes mellitus (disorder) | ... |  | Y | **?****+****-** |
| Contract | · · **Proteinuria due to type 1 diabetes mellitus (disorder)** | ... |  | Y | **?****+****-****++****--** |
|  | · · · Microalbuminuria due to type 1 diabetes mellitus (disorder) | ... |  | Y | **?****+****-** |
|  | · · Severe malnutrition due to type 1 diabetes mellitus (disorder) | ... |  | Y | **?****+****-** |
|  | · · Small vessel disease due to type 1 diabetes mellitus (disorder) | ... |  | Y | **?****+****-** |
|  | · · Ulcer of lower limb due to type 1 diabetes mellitus (disorder) | ... |  | Y | **?****+****-** |
|  | · · Vitreous hemorrhage due to type 1 diabetes mellitus (disorder) | ... |  | Y | **?****+****-** |
|  | · · Blindness co-occurrent and due to type 1 diabetes mellitus (disorder) | ... |  | Y | **?****+****-** |
|  | · · Disorder of nerve co-occurrent and due to type 1 diabetes mellitus (disorder) | ... |  | Y | **?****+****-** |
|  | · · Acidosis due to type 1 diabetes mellitus (disorder) | ... |  | Y | **?****+****-** |
|  | · · Dyslipidemia due to type 1 diabetes mellitus (disorder) | ... |  | Y | **?****+****-** |
|  | · · Hyperglycemia due to type 1 diabetes mellitus (disorder) | ... |  | Y | **?****+****-** |
|  | · · Hyperlipidemia due to type 1 diabetes mellitus (disorder) | ... |  | Y | **?****+****-** |
|  | · · Hyperosmolality due to uncontrolled type 1 diabetes mellitus (disorder) | ... |  | Y | **?****+****-** |
|  | · · Mixed hyperlipidemia due to type 1 diabetes mellitus (disorder) | ... |  | Y | **?****+****-** |
| Contract | · · **Foot ulcer due to type 1 diabetes mellitus (disorder)** | ... |  | Y | **?****+****-****++****--** |
|  | · · · Forefoot ulcer due to type 1 diabetes mellitus (disorder) | ... |  | Y | **?****+****-** |
|  | · · · Heel AND/OR midfoot ulcer due to type 1 diabetes mellitus (disorder) | ... |  | Y | **?****+****-** |
| Contract | · **Disorder due to type 2 diabetes mellitus (disorder)** | ... |  | Y | **?****+****-****++****--** |
|  | · · Angina associated with type II diabetes mellitus (disorder) | ... |  | Y | **?****+****-** |
|  | · · Cataract of eye due to diabetes mellitus type 2 (disorder) | ... |  | Y | **?****+****-** |
| Contract | · · **Chronic kidney disease due to type 2 diabetes mellitus (disorder)** | ... |  | Y | **?****+****-****++****--** |
|  | · · · Chronic kidney disease stage 1 due to type 2 diabetes mellitus (disorder) | ... |  | Y | **?****+****-** |
| Contract | · · · **Chronic kidney disease stage 2 due to type 2 diabetes mellitus (disorder)** | ... |  | Y | **?****+****-****++****--** |
|  | · · · · Hypertension in chronic kidney disease stage 2 due to type 2 diabetes mellitus (disorder) | ... |  | Y | **?****+****-** |
| Contract | · · · **Chronic kidney disease stage 3 due to type 2 diabetes mellitus (disorder)** | ... |  | Y | **?****+****-****++****--** |
|  | · · · · Hypertension in chronic kidney disease stage 3 due to type 2 diabetes mellitus (disorder) | ... |  | Y | **?****+****-** |
| Contract | · · · **Chronic kidney disease stage 4 due to type 2 diabetes mellitus (disorder)** | ... |  | Y | **?****+****-****++****--** |
|  | · · · · Hypertension in chronic kidney disease stage 4 due to type 2 diabetes mellitus (disorder) | ... |  | Y | **?****+****-** |
| Contract | · · · **Chronic kidney disease stage 5 due to type 2 diabetes mellitus (disorder)** | ... |  | Y | **?****+****-****++****--** |
|  | · · · · Hypertension in chronic kidney disease stage 5 due to type 2 diabetes mellitus (disorder) | ... |  | Y | **?****+****-** |
| Contract | · · · **Hypertension in chronic kidney disease due to type 2 diabetes mellitus (disorder)** | ... |  | Y | **?****+****-****++****--** |
|  | · · · · Hypertension in chronic kidney disease stage 2 due to type 2 diabetes mellitus (disorder) | ... |  | Y | **?****+****-** |
|  | · · · · Hypertension in chronic kidney disease stage 3 due to type 2 diabetes mellitus (disorder) | ... |  | Y | **?****+****-** |
|  | · · · · Hypertension in chronic kidney disease stage 4 due to type 2 diabetes mellitus (disorder) | ... |  | Y | **?****+****-** |
|  | · · · · Hypertension in chronic kidney disease stage 5 due to type 2 diabetes mellitus (disorder) | ... |  | Y | **?****+****-** |
|  | · · Diabetic dermopathy associated with diabetes mellitus type 2 (disorder) | ... |  | Y | **?****+****-** |
|  | · · Disorder associated with well controlled type 2 diabetes mellitus (disorder) | ... |  | Y | **?****+****-** |
| Contract | · · **Dyslipidemia due to type 2 diabetes mellitus (disorder)** | ... |  | Y | **?****+****-****++****--** |
|  | · · · Dyslipidemia with high density lipoprotein below reference range and triglyceride above reference range due to type 2 diabetes mellitus (disorder) | ... |  | Y | **?****+****-** |
| Contract | · · **End stage renal disease on dialysis due to type 2 diabetes mellitus (disorder)** | ... |  | Y | **?****+****-****++****--** |
|  | · · · Hypertension concurrent and due to end stage renal disease on dialysis due to type 2 diabetes mellitus (disorder) | ... |  | Y | **?****+****-** |
| Contract | · · **Hypoglycemia due to type 2 diabetes mellitus (disorder)** | ... |  | Y | **?****+****-****++****--** |
|  | · · · Insulin reactive hypoglycemia in type 2 diabetes mellitus (disorder) | ... |  | Y | **?****+****-** |
|  | · · Ketoacidotic coma in type II diabetes mellitus (disorder) | ... |  | Y | **?****+****-** |
|  | · · Macular edema due to type 2 diabetes mellitus (disorder) | ... |  | Y | **?****+****-** |
|  | · · Osteomyelitis due to type 2 diabetes mellitus (disorder) | ... |  | Y | **?****+****-** |
| Contract | · · **Proteinuria due to type 2 diabetes mellitus (disorder)** | ... |  | Y | **?****+****-****++****--** |
|  | · · · Microalbuminuria due to type 2 diabetes mellitus (disorder) | ... |  | Y | **?****+****-** |
|  | · · Severe malnutrition due to type 2 diabetes mellitus (disorder) | ... |  | Y | **?****+****-** |
|  | · · Small vessel disease due to type 2 diabetes mellitus (disorder) | ... |  | Y | **?****+****-** |
|  | · · Type II diabetes mellitus with multiple complications (disorder) | ... |  | Y | **?****+****-** |
| Contract | · · **Ulcer of lower extremity due to diabetes mellitus type 2 (disorder)** | ... |  | Y | **?****+****-****++****--** |
|  | · · · Ankle ulcer due to type 2 diabetes mellitus (disorder) | ... |  | Y | **?****+****-** |
|  | · · · Stasis ulcer due to type 2 diabetes mellitus (disorder) | ... |  | Y | **?****+****-** |
| Contract | · · · **Foot ulcer due to type 2 diabetes mellitus (disorder)** | ... |  | Y | **?****+****-****++****--** |
|  | · · · · Forefoot ulcer due to type 2 diabetes mellitus (disorder) | ... |  | Y | **?****+****-** |
|  | · · · · Heel AND/OR midfoot ulcer due to type 2 diabetes mellitus (disorder) | ... |  | Y | **?****+****-** |
|  | · · · · Ischemic foot ulcer due to type 2 diabetes mellitus (disorder) | ... |  | Y | **?****+****-** |
| Contract | · · · · **Neuropathic foot ulcer due to type 2 diabetes mellitus (disorder)** | ... |  | Y | **?****+****-****++****--** |
|  | · · · · · Neuropathic ulcer of midfoot AND/OR heel due to type 2 diabetes mellitus (disorder) | ... |  | Y | **?****+****-** |
|  | · · · · Ulcer of left foot co-occurrent and due to diabetes mellitus type 2 (disorder) | ... |  | Y | **?****+****-** |
|  | · · · · Ulcer of right foot co-occurrent and due to diabetes mellitus type 2 (disorder) | ... |  | Y | **?****+****-** |
| Contract | · · · · **Ulcer of toe due to type 2 diabetes mellitus (disorder)** | ... |  | Y | **?****+****-****++****--** |
|  | · · · · · Neuropathic toe ulcer due to type 2 diabetes mellitus (disorder) | ... |  | Y | **?****+****-** |
|  | · · · · · Skin ulcer of toe due to diabetes mellitus type 2 (disorder) | ... |  | Y | **?****+****-** |
|  | · · · · Ischemic foot ulcer due to type 2 diabetes mellitus (disorder) | ... |  | Y | **?****+****-** |
| Contract | · · **Ulcer of skin co-occurrent and due to type 2 diabetes mellitus (disorder)** | ... |  | Y | **?****+****-****++****--** |
|  | · · · Skin ulcer of toe due to diabetes mellitus type 2 (disorder) | ... |  | Y | **?****+****-** |
|  | · · Vitreous hemorrhage due to type 2 diabetes mellitus (disorder) | ... |  | Y | **?****+****-** |
|  | · · Blindness co-occurrent and due to type 2 diabetes mellitus (disorder) | ... |  | Y | **?****+****-** |
|  | · · Acidosis due to type 2 diabetes mellitus (disorder) | ... |  | Y | **?****+****-** |
|  | · · Hyperglycemia due to type 2 diabetes mellitus (disorder) | ... |  | Y | **?****+****-** |
|  | · · Hyperlipidemia due to type 2 diabetes mellitus (disorder) | ... |  | Y | **?****+****-** |
|  | · · Mixed hyperlipidemia due to type II diabetes mellitus (disorder) | ... |  | Y | **?****+****-** |
|  | · · Cheiropathy due to type 2 diabetes mellitus (disorder) | ... |  | Y | **?****+****-** |
| Contract | · **Disorder of eye due to diabetes mellitus (disorder)** | ... |  | Y | **?****+****-****++****--** |
| Contract | · · **Cataract of eye due to diabetes mellitus (disorder)** | ... |  | Y | **?****+****-****++****--** |
| Contract | · · · **Cataract of left eye due to diabetes mellitus (disorder)** | ... |  | Y | **?****+****-****++****--** |
|  | · · · · Cataract of bilateral eyes due to diabetes mellitus (disorder) | ... |  | Y | **?****+****-** |
| Contract | · · · **Cataract of right eye due to diabetes mellitus (disorder)** | ... |  | Y | **?****+****-****++****--** |
|  | · · · · Cataract of bilateral eyes due to diabetes mellitus (disorder) | ... |  | Y | **?****+****-** |
|  | · · · Cataract of eye due to diabetes mellitus type 1 (disorder) | ... |  | Y | **?****+****-** |
|  | · · · Cataract of eye due to diabetes mellitus type 2 (disorder) | ... |  | Y | **?****+****-** |
| Contract | · · **Iritis due to diabetes mellitus (disorder)** | ... |  | Y | **?****+****-****++****--** |
| Contract | · · · **Iritis of left eye due to diabetes mellitus (disorder)** | ... |  | Y | **?****+****-****++****--** |
|  | · · · · Iritis of bilateral eyes due to diabetes mellitus (disorder) | ... |  | Y | **?****+****-** |
| Contract | · · · **Iritis of right eye due to diabetes mellitus (disorder)** | ... |  | Y | **?****+****-****++****--** |
|  | · · · · Iritis of bilateral eyes due to diabetes mellitus (disorder) | ... |  | Y | **?****+****-** |
| Contract | · · **Macular edema due to diabetes mellitus (disorder)** | ... |  | Y | **?****+****-****++****--** |
|  | · · · Macular edema of left eye due to diabetes mellitus (disorder) | ... |  | Y | **?****+****-** |
|  | · · · Macular edema of right eye due to diabetes mellitus (disorder) | ... |  | Y | **?****+****-** |
|  | · · · Macular edema due to type 1 diabetes mellitus (disorder) | ... |  | Y | **?****+****-** |
|  | · · · Macular edema due to type 2 diabetes mellitus (disorder) | ... |  | Y | **?****+****-** |
|  | · · Ophthalmic complication of malnutrition-related diabetes mellitus (disorder) | ... |  | Y | **?****+****-** |
| Contract | · · **Vitreous hemorrhage due to diabetes mellitus (disorder)** | ... |  | Y | **?****+****-****++****--** |
|  | · · · Vitreous hemorrhage of left eye due to diabetes mellitus (disorder) | ... |  | Y | **?****+****-** |
|  | · · · Vitreous hemorrhage of right eye due to diabetes mellitus (disorder) | ... |  | Y | **?****+****-** |
|  | · · · Vitreous hemorrhage due to type 1 diabetes mellitus (disorder) | ... |  | Y | **?****+****-** |
|  | · · · Vitreous hemorrhage due to type 2 diabetes mellitus (disorder) | ... |  | Y | **?****+****-** |
|  | · · Disorder of eye co-occurrent and due to type 1 diabetes mellitus (disorder) | ... |  | Y | **?****+****-** |
|  | · · Disorder of eye co-occurrent and due to type 2 diabetes mellitus (disorder) | ... |  | Y | **?****+****-** |
|  | · · Neovascular glaucoma co-occurrent and due to diabetes mellitus (disorder) | ... |  | Y | **?****+****-** |
| Contract | · · **Retinopathy co-occurrent and due to diabetes mellitus (disorder)** | ... |  | Y | **?****+****-****++****--** |
|  | · · · Diabetic intraretinal microvascular anomaly (disorder) | ... |  | Y | **?****+****-** |
|  | · · · Retinal microaneurysm co-occurrent and due to diabetes mellitus (disorder) | ... |  | Y | **?****+****-** |
|  | · · · Retinal venous beading co-occurrent and due to diabetes mellitus (disorder) | ... |  | Y | **?****+****-** |
|  | · · · Advanced retinal disease co-occurrent and due to diabetes mellitus (disorder) | ... |  | Y | **?****+****-** |
|  | · · · Diabetic retinopathy detected by national screening programme (disorder) | ... |  | Y | **?****+****-** |
| Contract | · · · **Disorder of macula co-occurrent and due to diabetes mellitus (disorder)** | ... |  | Y | **?****+****-****++****--** |
|  | · · · · Ischemic maculopathy co-occurrent and due to diabetes mellitus (disorder) | ... |  | Y | **?****+****-** |
|  | · · · · Advanced maculopathy co-occurrent and due to diabetes mellitus (disorder) | ... |  | Y | **?****+****-** |
|  | · · · · Disorder of left macula co-occurrent and due to diabetes mellitus (disorder) | ... |  | Y | **?****+****-** |
|  | · · · · Disorder of right macula co-occurrent and due to diabetes mellitus (disorder) | ... |  | Y | **?****+****-** |
|  | · · · · Ischemic maculopathy co-occurrent and due to diabetes mellitus (disorder) | ... |  | Y | **?****+****-** |
|  | · · · · · Mixed maculopathy co-occurrent and due to diabetes mellitus (disorder) | ... |  | Y | **?****+****-** |
| Contract | · · · **Nonproliferative retinopathy co-occurrent and due to diabetes mellitus (disorder)** | ... |  | Y | **?****+****-****++****--** |
| Contract | · · · · **Mild nonproliferative retinopathy co-occurrent and due to diabetes mellitus (disorder)** | ... |  | Y | **?****+****-****++****--** |
|  | · · · · · Mild nonproliferative retinopathy of left eye co-occurrent and due to diabetes mellitus (disorder) | ... |  | Y | **?****+****-** |
|  | · · · · · Mild nonproliferative retinopathy of right eye co-occurrent and due to diabetes mellitus (disorder) | ... |  | Y | **?****+****-** |
| Contract | · · · · **Moderate nonproliferative retinopathy co-occurrent and due to diabetes mellitus (disorder)** | ... |  | Y | **?****+****-****++****--** |
|  | · · · · · Moderate nonproliferative retinopathy of left eye co-occurrent and due to diabetes mellitus (disorder) | ... |  | Y | **?****+****-** |
|  | · · · · · Moderate nonproliferative retinopathy of right eye co-occurrent and due to diabetes mellitus (disorder) | ... |  | Y | **?****+****-** |
| Contract | · · · · **Preproliferative retinopathy co-occurrent and due to diabetes mellitus (disorder)** | ... |  | Y | **?****+****-****++****--** |
|  | · · · · · On examination - left eye preproliferative diabetic retinopathy (disorder) | ... |  | Y | **?****+****-** |
|  | · · · · · On examination - right eye preproliferative diabetic retinopathy (disorder) | ... |  | Y | **?****+****-** |
|  | · · · · · Preproliferative retinopathy of left eye co-occurrent and due to diabetes mellitus (disorder) | ... |  | Y | **?****+****-** |
|  | · · · · · Preproliferative retinopathy of right eye co-occurrent and due to diabetes mellitus (disorder) | ... |  | Y | **?****+****-** |
| Contract | · · · · **Severe nonproliferative retinopathy co-occurrent and due to diabetes mellitus (disorder)** | ... |  | Y | **?****+****-****++****--** |
|  | · · · · · Severe nonproliferative retinopathy of left eye co-occurrent and due to diabetes mellitus (disorder) | ... |  | Y | **?****+****-** |
|  | · · · · · Severe nonproliferative retinopathy of right eye co-occurrent and due to diabetes mellitus (disorder) | ... |  | Y | **?****+****-** |
|  | · · · · · Severe nonproliferative retinopathy without macular edema co-occurrent and due to diabetes mellitus (disorder) | ... |  | Y | **?****+****-** |
| Contract | · · · · **Very severe nonproliferative retinopathy co-occurrent and due to diabetes mellitus (disorder)** | ... |  | Y | **?****+****-****++****--** |
|  | · · · · · Very severe nonproliferative retinopathy of left eye co-occurrent and due to diabetes mellitus (disorder) | ... |  | Y | **?****+****-** |
|  | · · · · · Very severe nonproliferative retinopathy of right eye co-occurrent and due to diabetes mellitus (disorder) | ... |  | Y | **?****+****-** |
|  | · · · · · Very severe nonproliferative retinopathy without macular edema co-occurrent and due to diabetes mellitus (disorder) | ... |  | Y | **?****+****-** |
|  | · · · · Nonproliferative retinopathy co-occurrent and due to secondary diabetes mellitus (disorder) | ... |  | Y | **?****+****-** |
|  | · · · · · Mild nonproliferative retinopathy co-occurrent and due to secondary diabetes mellitus (disorder) | ... |  | Y | **?****+****-** |
|  | · · · · · Moderate nonproliferative retinopathy co-occurrent and due to secondary diabetes mellitus (disorder) | ... |  | Y | **?****+****-** |
|  | · · · · · Mild nonproliferative retinopathy co-occurrent and due to secondary diabetes mellitus (disorder) | ... |  | Y | **?****+****-** |
|  | · · · · · Moderate nonproliferative retinopathy co-occurrent and due to secondary diabetes mellitus (disorder) | ... |  | Y | **?****+****-** |
|  | · · · · · Mild nonproliferative retinopathy co-occurrent and due to secondary diabetes mellitus (disorder) | ... |  | Y | **?****+****-** |
|  | · · · · · Moderate nonproliferative retinopathy co-occurrent and due to secondary diabetes mellitus (disorder) | ... |  | Y | **?****+****-** |
|  | · · · On examination - left eye background diabetic retinopathy (disorder) | ... |  | Y | **?****+****-** |
|  | · · · On examination - right eye background diabetic retinopathy (disorder) | ... |  | Y | **?****+****-** |
|  | · · · On examination - sight threatening diabetic retinopathy (disorder) | ... |  | Y | **?****+****-** |
| Contract | · · · **Proliferative retinopathy co-occurrent and due to diabetes mellitus (disorder)** | ... |  | Y | **?****+****-****++****--** |
|  | · · · · Non-high-risk proliferative diabetic retinopathy with no macular edema (disorder) | ... |  | Y | **?****+****-** |
| Contract | · · · · **On examination - left eye proliferative diabetic retinopathy (disorder)** | ... |  | Y | **?****+****-****++****--** |
|  | · · · · · On examination - left eye stable treated proliferative diabetic retinopathy (disorder) | ... |  | Y | **?****+****-** |
| Contract | · · · · **On examination - right eye proliferative diabetic retinopathy (disorder)** | ... |  | Y | **?****+****-****++****--** |
|  | · · · · · On examination - right eye stable treated proliferative diabetic retinopathy (disorder) | ... |  | Y | **?****+****-** |
| Contract | · · · · **Proliferative diabetic retinopathy - high risk (disorder)** | ... |  | Y | **?****+****-****++****--** |
|  | · · · · · Proliferative diabetic retinopathy - high risk with clinically significant macular edema (disorder) | ... |  | Y | **?****+****-** |
|  | · · · · · High risk proliferative diabetic retinopathy not amenable to photocoagulation (disorder) | ... |  | Y | **?****+****-** |
|  | · · · · · Proliferative diabetic retinopathy - high risk with no macular edema (disorder) | ... |  | Y | **?****+****-** |
|  | · · · · · Very severe proliferative diabetic retinopathy (disorder) | ... |  | Y | **?****+****-** |
|  | · · · · · Proliferative diabetic retinopathy - high risk with clinically significant macular edema (disorder) | ... |  | Y | **?****+****-** |
|  | · · · · Proliferative diabetic retinopathy - iris neovascularization (disorder) | ... |  | Y | **?****+****-** |
| Contract | · · · · **Proliferative diabetic retinopathy - non high risk (disorder)** | ... |  | Y | **?****+****-****++****--** |
|  | · · · · · Proliferative diabetic retinopathy - quiescent (disorder) | ... |  | Y | **?****+****-** |
|  | · · · · Proliferative diabetic retinopathy following surgery (disorder) | ... |  | Y | **?****+****-** |
|  | · · · · Proliferative diabetic retinopathy with new vessels elsewhere than on disc (disorder) | ... |  | Y | **?****+****-** |
|  | · · · · Proliferative diabetic retinopathy with new vessels on disc (disorder) | ... |  | Y | **?****+****-** |
|  | · · · · Proliferative retinopathy of left eye co-occurrent and due to diabetes mellitus (disorder) | ... |  | Y | **?****+****-** |
|  | · · · · Proliferative retinopathy of right eye co-occurrent and due to diabetes mellitus (disorder) | ... |  | Y | **?****+****-** |
| Contract | · · · **Retinal edema co-occurrent and due to diabetes mellitus (disorder)** | ... |  | Y | **?****+****-****++****--** |
|  | · · · · Clinically significant macular edema co-occurrent and due to diabetes mellitus (disorder) | ... |  | Y | **?****+****-** |
|  | · · · · Diffuse exudative maculopathy co-occurrent and due to diabetes mellitus (disorder) | ... |  | Y | **?****+****-** |
|  | · · · · Focal exudative maculopathy co-occurrent and due to diabetes mellitus (disorder) | ... |  | Y | **?****+****-** |
|  | · · · · Macular edema not clinically significant co-occurrent and due to diabetes mellitus (disorder) | ... |  | Y | **?****+****-** |
|  | · · · · Non-high-risk proliferative diabetic retinopathy with clinically significant macular edema (disorder) | ... |  | Y | **?****+****-** |
|  | · · · · Clinically significant macular edema co-occurrent and due to diabetes mellitus (disorder) | ... |  | Y | **?****+****-** |
|  | · · · · Diffuse exudative maculopathy co-occurrent and due to diabetes mellitus (disorder) | ... |  | Y | **?****+****-** |
|  | · · · · Focal exudative maculopathy co-occurrent and due to diabetes mellitus (disorder) | ... |  | Y | **?****+****-** |
|  | · · · · Macular edema not clinically significant co-occurrent and due to diabetes mellitus (disorder) | ... |  | Y | **?****+****-** |
|  | · · · · Clinically significant macular edema co-occurrent and due to diabetes mellitus (disorder) | ... |  | Y | **?****+****-** |
|  | · · · · Diffuse exudative maculopathy co-occurrent and due to diabetes mellitus (disorder) | ... |  | Y | **?****+****-** |
|  | · · · · Focal exudative maculopathy co-occurrent and due to diabetes mellitus (disorder) | ... |  | Y | **?****+****-** |
|  | · · · · Macular edema not clinically significant co-occurrent and due to diabetes mellitus (disorder) | ... |  | Y | **?****+****-** |
| Contract | · · · · **Clinically significant macular edema co-occurrent and due to diabetes mellitus (disorder)** | ... |  | Y | **?****+****-****++****--** |
|  | · · · · · Clinically significant macular edema of left eye co-occurrent and due to diabetes mellitus (disorder) | ... |  | Y | **?****+****-** |
|  | · · · · · Clinically significant macular edema of right eye co-occurrent and due to diabetes mellitus (disorder) | ... |  | Y | **?****+****-** |
|  | · · · · · Severe nonproliferative retinopathy with clinically significant macular edema co-occurrent and due to diabetes mellitus (disorder) | ... |  | Y | **?****+****-** |
|  | · · · · · Very severe nonproliferative retinopathy with clinically significant macular edema co-occurrent and due to diabetes mellitus (disorder) | ... |  | Y | **?****+****-** |
|  | · · · · · Clinically significant macular edema of left eye co-occurrent and due to diabetes mellitus (disorder) | ... |  | Y | **?****+****-** |
|  | · · · · · Clinically significant macular edema of right eye co-occurrent and due to diabetes mellitus (disorder) | ... |  | Y | **?****+****-** |
|  | · · · · · Severe nonproliferative retinopathy with clinically significant macular edema co-occurrent and due to diabetes mellitus (disorder) | ... |  | Y | **?****+****-** |
|  | · · · · · Very severe nonproliferative retinopathy with clinically significant macular edema co-occurrent and due to diabetes mellitus (disorder) | ... |  | Y | **?****+****-** |
|  | · · · · · Clinically significant macular edema of left eye co-occurrent and due to diabetes mellitus (disorder) | ... |  | Y | **?****+****-** |
|  | · · · · · Clinically significant macular edema of right eye co-occurrent and due to diabetes mellitus (disorder) | ... |  | Y | **?****+****-** |
|  | · · · · · Severe nonproliferative retinopathy with clinically significant macular edema co-occurrent and due to diabetes mellitus (disorder) | ... |  | Y | **?****+****-** |
|  | · · · · · Very severe nonproliferative retinopathy with clinically significant macular edema co-occurrent and due to diabetes mellitus (disorder) | ... |  | Y | **?****+****-** |
|  | · · · · · Clinically significant macular edema of left eye co-occurrent and due to diabetes mellitus (disorder) | ... |  | Y | **?****+****-** |
|  | · · · · · Clinically significant macular edema of right eye co-occurrent and due to diabetes mellitus (disorder) | ... |  | Y | **?****+****-** |
|  | · · · · · Severe nonproliferative retinopathy with clinically significant macular edema co-occurrent and due to diabetes mellitus (disorder) | ... |  | Y | **?****+****-** |
|  | · · · · · Very severe nonproliferative retinopathy with clinically significant macular edema co-occurrent and due to diabetes mellitus (disorder) | ... |  | Y | **?****+****-** |
|  | · · · · · Clinically significant macular edema of left eye co-occurrent and due to diabetes mellitus (disorder) | ... |  | Y | **?****+****-** |
|  | · · · · · Clinically significant macular edema of right eye co-occurrent and due to diabetes mellitus (disorder) | ... |  | Y | **?****+****-** |
|  | · · · · · Severe nonproliferative retinopathy with clinically significant macular edema co-occurrent and due to diabetes mellitus (disorder) | ... |  | Y | **?****+****-** |
|  | · · · · · Very severe nonproliferative retinopathy with clinically significant macular edema co-occurrent and due to diabetes mellitus (disorder) | ... |  | Y | **?****+****-** |
|  | · · · · Diffuse exudative maculopathy co-occurrent and due to diabetes mellitus (disorder) | ... |  | Y | **?****+****-** |
|  | · · · · Focal exudative maculopathy co-occurrent and due to diabetes mellitus (disorder) | ... |  | Y | **?****+****-** |
|  | · · · · Macular edema not clinically significant co-occurrent and due to diabetes mellitus (disorder) | ... |  | Y | **?****+****-** |
|  | · · · · Non-high-risk proliferative diabetic retinopathy with clinically significant macular edema (disorder) | ... |  | Y | **?****+****-** |
|  | · · · Retinal ischemia co-occurrent and due to diabetes mellitus (disorder) | ... |  | Y | **?****+****-** |
|  | · · · Traction retinal detachment co-occurrent and due to diabetes mellitus (disorder) | ... |  | Y | **?****+****-** |
|  | · · · Visually threatening diabetic retinopathy (disorder) | ... |  | Y | **?****+****-** |
|  | · · · Diabetic intraretinal microvascular anomaly (disorder) | ... |  | Y | **?****+****-** |
|  | · · · Retinal microaneurysm co-occurrent and due to diabetes mellitus (disorder) | ... |  | Y | **?****+****-** |
|  | · · · Retinal venous beading co-occurrent and due to diabetes mellitus (disorder) | ... |  | Y | **?****+****-** |
|  | · · · Diabetic intraretinal microvascular anomaly (disorder) | ... |  | Y | **?****+****-** |
| Contract | · · · **Retinal microaneurysm co-occurrent and due to diabetes mellitus (disorder)** | ... |  | Y | **?****+****-****++****--** |
|  | · · · · Retinal microaneurysm of left eye co-occurrent and due to diabetes mellitus (disorder) | ... |  | Y | **?****+****-** |
|  | · · · · Retinal microaneurysm of right eye co-occurrent and due to diabetes mellitus (disorder) | ... |  | Y | **?****+****-** |
|  | · · · · Retinal microaneurysm of left eye co-occurrent and due to diabetes mellitus (disorder) | ... |  | Y | **?****+****-** |
|  | · · · · Retinal microaneurysm of right eye co-occurrent and due to diabetes mellitus (disorder) | ... |  | Y | **?****+****-** |
|  | · · · · Retinal microaneurysm of left eye co-occurrent and due to diabetes mellitus (disorder) | ... |  | Y | **?****+****-** |
|  | · · · · Retinal microaneurysm of right eye co-occurrent and due to diabetes mellitus (disorder) | ... |  | Y | **?****+****-** |
|  | · · · · Retinal microaneurysm of left eye co-occurrent and due to diabetes mellitus (disorder) | ... |  | Y | **?****+****-** |
|  | · · · · Retinal microaneurysm of right eye co-occurrent and due to diabetes mellitus (disorder) | ... |  | Y | **?****+****-** |
|  | · · · · Retinal microaneurysm of left eye co-occurrent and due to diabetes mellitus (disorder) | ... |  | Y | **?****+****-** |
|  | · · · · Retinal microaneurysm of right eye co-occurrent and due to diabetes mellitus (disorder) | ... |  | Y | **?****+****-** |
| Contract | · · · **Retinal venous beading co-occurrent and due to diabetes mellitus (disorder)** | ... |  | Y | **?****+****-****++****--** |
|  | · · · · Retinal venous beading of left eye co-occurrent and due to diabetes mellitus (disorder) | ... |  | Y | **?****+****-** |
|  | · · · · Retinal venous beading of right eye co-occurrent and due to diabetes mellitus (disorder) | ... |  | Y | **?****+****-** |
|  | · · · · Retinal venous beading of left eye co-occurrent and due to diabetes mellitus (disorder) | ... |  | Y | **?****+****-** |
|  | · · · · Retinal venous beading of right eye co-occurrent and due to diabetes mellitus (disorder) | ... |  | Y | **?****+****-** |
|  | · · · · Retinal venous beading of left eye co-occurrent and due to diabetes mellitus (disorder) | ... |  | Y | **?****+****-** |
|  | · · · · Retinal venous beading of right eye co-occurrent and due to diabetes mellitus (disorder) | ... |  | Y | **?****+****-** |
|  | · · · · Retinal venous beading of left eye co-occurrent and due to diabetes mellitus (disorder) | ... |  | Y | **?****+****-** |
|  | · · · · Retinal venous beading of right eye co-occurrent and due to diabetes mellitus (disorder) | ... |  | Y | **?****+****-** |
|  | · · · · Retinal venous beading of left eye co-occurrent and due to diabetes mellitus (disorder) | ... |  | Y | **?****+****-** |
|  | · · · · Retinal venous beading of right eye co-occurrent and due to diabetes mellitus (disorder) | ... |  | Y | **?****+****-** |
| Contract | · · **Disorder of eye co-occurrent and due to type 1 diabetes mellitus (disorder)** | ... |  | Y | **?****+****-****++****--** |
|  | · · · Rubeosis iridis co-occurrent and due to type 1 diabetes mellitus (disorder) | ... |  | Y | **?****+****-** |
|  | · · · Retinopathy co-occurrent and due to type 1 diabetes mellitus (disorder) | ... |  | Y | **?****+****-** |
|  | · · · Rubeosis iridis co-occurrent and due to type 1 diabetes mellitus (disorder) | ... |  | Y | **?****+****-** |
|  | · · · Retinopathy co-occurrent and due to type 1 diabetes mellitus (disorder) | ... |  | Y | **?****+****-** |
|  | · · · · Nonproliferative retinopathy co-occurrent and due to type 1 diabetes mellitus (disorder) | ... |  | Y | **?****+****-** |
|  | · · · · Proliferative retinopathy co-occurrent and due to type 1 diabetes mellitus (disorder) | ... |  | Y | **?****+****-** |
|  | · · · · Retinal edema co-occurrent and due to type 1 diabetes mellitus (disorder) | ... |  | Y | **?****+****-** |
|  | · · · · Retinal ischemia co-occurrent and due to type 1 diabetes mellitus (disorder) | ... |  | Y | **?****+****-** |
|  | · · · · Traction retinal detachment co-occurrent and due to type 1 diabetes mellitus (disorder) | ... |  | Y | **?****+****-** |
|  | · · · · Nonproliferative retinopathy co-occurrent and due to type 1 diabetes mellitus (disorder) | ... |  | Y | **?****+****-** |
|  | · · · · Proliferative retinopathy co-occurrent and due to type 1 diabetes mellitus (disorder) | ... |  | Y | **?****+****-** |
|  | · · · · Retinal edema co-occurrent and due to type 1 diabetes mellitus (disorder) | ... |  | Y | **?****+****-** |
|  | · · · · Retinal ischemia co-occurrent and due to type 1 diabetes mellitus (disorder) | ... |  | Y | **?****+****-** |
|  | · · · · Traction retinal detachment co-occurrent and due to type 1 diabetes mellitus (disorder) | ... |  | Y | **?****+****-** |
|  | · · · · Nonproliferative retinopathy co-occurrent and due to type 1 diabetes mellitus (disorder) | ... |  | Y | **?****+****-** |
|  | · · · · Proliferative retinopathy co-occurrent and due to type 1 diabetes mellitus (disorder) | ... |  | Y | **?****+****-** |
|  | · · · · Retinal edema co-occurrent and due to type 1 diabetes mellitus (disorder) | ... |  | Y | **?****+****-** |
|  | · · · · Retinal ischemia co-occurrent and due to type 1 diabetes mellitus (disorder) | ... |  | Y | **?****+****-** |
|  | · · · · Traction retinal detachment co-occurrent and due to type 1 diabetes mellitus (disorder) | ... |  | Y | **?****+****-** |
|  | · · · · Nonproliferative retinopathy co-occurrent and due to type 1 diabetes mellitus (disorder) | ... |  | Y | **?****+****-** |
|  | · · · · Proliferative retinopathy co-occurrent and due to type 1 diabetes mellitus (disorder) | ... |  | Y | **?****+****-** |
|  | · · · · Retinal edema co-occurrent and due to type 1 diabetes mellitus (disorder) | ... |  | Y | **?****+****-** |
|  | · · · · Retinal ischemia co-occurrent and due to type 1 diabetes mellitus (disorder) | ... |  | Y | **?****+****-** |
|  | · · · · Traction retinal detachment co-occurrent and due to type 1 diabetes mellitus (disorder) | ... |  | Y | **?****+****-** |
|  | · · · · Nonproliferative retinopathy co-occurrent and due to type 1 diabetes mellitus (disorder) | ... |  | Y | **?****+****-** |
|  | · · · · · Mild nonproliferative retinopathy co-occurrent and due to type 1 diabetes mellitus (disorder) | ... |  | Y | **?****+****-** |
|  | · · · · · Moderate nonproliferative retinopathy co-occurrent and due to type 1 diabetes mellitus (disorder) | ... |  | Y | **?****+****-** |
|  | · · · · · Mild nonproliferative retinopathy co-occurrent and due to type 1 diabetes mellitus (disorder) | ... |  | Y | **?****+****-** |
|  | · · · · · Moderate nonproliferative retinopathy co-occurrent and due to type 1 diabetes mellitus (disorder) | ... |  | Y | **?****+****-** |
|  | · · · · · Mild nonproliferative retinopathy co-occurrent and due to type 1 diabetes mellitus (disorder) | ... |  | Y | **?****+****-** |
|  | · · · · · Moderate nonproliferative retinopathy co-occurrent and due to type 1 diabetes mellitus (disorder) | ... |  | Y | **?****+****-** |
|  | · · · · · Mild nonproliferative retinopathy co-occurrent and due to type 1 diabetes mellitus (disorder) | ... |  | Y | **?****+****-** |
|  | · · · · · Moderate nonproliferative retinopathy co-occurrent and due to type 1 diabetes mellitus (disorder) | ... |  | Y | **?****+****-** |
|  | · · · · · Mild nonproliferative retinopathy co-occurrent and due to type 1 diabetes mellitus (disorder) | ... |  | Y | **?****+****-** |
|  | · · · · · Moderate nonproliferative retinopathy co-occurrent and due to type 1 diabetes mellitus (disorder) | ... |  | Y | **?****+****-** |
|  | · · · · · Mild nonproliferative retinopathy co-occurrent and due to type 1 diabetes mellitus (disorder) | ... |  | Y | **?****+****-** |
|  | · · · · · Moderate nonproliferative retinopathy co-occurrent and due to type 1 diabetes mellitus (disorder) | ... |  | Y | **?****+****-** |
|  | · · · · · Mild nonproliferative retinopathy co-occurrent and due to type 1 diabetes mellitus (disorder) | ... |  | Y | **?****+****-** |
|  | · · · · · Moderate nonproliferative retinopathy co-occurrent and due to type 1 diabetes mellitus (disorder) | ... |  | Y | **?****+****-** |
|  | · · · · Proliferative retinopathy co-occurrent and due to type 1 diabetes mellitus (disorder) | ... |  | Y | **?****+****-** |
|  | · · · · Retinal edema co-occurrent and due to type 1 diabetes mellitus (disorder) | ... |  | Y | **?****+****-** |
|  | · · · · · Exudative maculopathy co-occurrent and due to type 1 diabetes mellitus (disorder) | ... |  | Y | **?****+****-** |
|  | · · · · Retinal ischemia co-occurrent and due to type 1 diabetes mellitus (disorder) | ... |  | Y | **?****+****-** |
|  | · · · · Traction retinal detachment co-occurrent and due to type 1 diabetes mellitus (disorder) | ... |  | Y | **?****+****-** |
| Contract | · · **Disorder of eye co-occurrent and due to type 2 diabetes mellitus (disorder)** | ... |  | Y | **?****+****-****++****--** |
|  | · · · Glaucoma due to type 2 diabetes mellitus (disorder) | ... |  | Y | **?****+****-** |
|  | · · · Rubeosis iridis co-occurrent and due to type 2 diabetes mellitus (disorder) | ... |  | Y | **?****+****-** |
|  | · · · Retinopathy co-occurrent and due to type 2 diabetes mellitus (disorder) | ... |  | Y | **?****+****-** |
|  | · · · Glaucoma due to type 2 diabetes mellitus (disorder) | ... |  | Y | **?****+****-** |
|  | · · · Rubeosis iridis co-occurrent and due to type 2 diabetes mellitus (disorder) | ... |  | Y | **?****+****-** |
|  | · · · Retinopathy co-occurrent and due to type 2 diabetes mellitus (disorder) | ... |  | Y | **?****+****-** |
|  | · · · · Nonproliferative retinopathy co-occurrent and due to type 2 diabetes mellitus (disorder) | ... |  | Y | **?****+****-** |
|  | · · · · Proliferative retinopathy co-occurrent and due to type 2 diabetes mellitus (disorder) | ... |  | Y | **?****+****-** |
|  | · · · · Retinal edema co-occurrent and due to type 2 diabetes mellitus (disorder) | ... |  | Y | **?****+****-** |
|  | · · · · Retinal ischemia co-occurrent and due to type 2 diabetes mellitus (disorder) | ... |  | Y | **?****+****-** |
|  | · · · · Traction retinal detachment co-occurrent and due to type 2 diabetes mellitus (disorder) | ... |  | Y | **?****+****-** |
|  | · · · · Nonproliferative retinopathy co-occurrent and due to type 2 diabetes mellitus (disorder) | ... |  | Y | **?****+****-** |
|  | · · · · Proliferative retinopathy co-occurrent and due to type 2 diabetes mellitus (disorder) | ... |  | Y | **?****+****-** |
|  | · · · · Retinal edema co-occurrent and due to type 2 diabetes mellitus (disorder) | ... |  | Y | **?****+****-** |
|  | · · · · Retinal ischemia co-occurrent and due to type 2 diabetes mellitus (disorder) | ... |  | Y | **?****+****-** |
|  | · · · · Traction retinal detachment co-occurrent and due to type 2 diabetes mellitus (disorder) | ... |  | Y | **?****+****-** |
|  | · · · · Nonproliferative retinopathy co-occurrent and due to type 2 diabetes mellitus (disorder) | ... |  | Y | **?****+****-** |
|  | · · · · Proliferative retinopathy co-occurrent and due to type 2 diabetes mellitus (disorder) | ... |  | Y | **?****+****-** |
|  | · · · · Retinal edema co-occurrent and due to type 2 diabetes mellitus (disorder) | ... |  | Y | **?****+****-** |
|  | · · · · Retinal ischemia co-occurrent and due to type 2 diabetes mellitus (disorder) | ... |  | Y | **?****+****-** |
|  | · · · · Traction retinal detachment co-occurrent and due to type 2 diabetes mellitus (disorder) | ... |  | Y | **?****+****-** |
|  | · · · · Nonproliferative retinopathy co-occurrent and due to type 2 diabetes mellitus (disorder) | ... |  | Y | **?****+****-** |
|  | · · · · Proliferative retinopathy co-occurrent and due to type 2 diabetes mellitus (disorder) | ... |  | Y | **?****+****-** |
|  | · · · · Retinal edema co-occurrent and due to type 2 diabetes mellitus (disorder) | ... |  | Y | **?****+****-** |
|  | · · · · Retinal ischemia co-occurrent and due to type 2 diabetes mellitus (disorder) | ... |  | Y | **?****+****-** |
|  | · · · · Traction retinal detachment co-occurrent and due to type 2 diabetes mellitus (disorder) | ... |  | Y | **?****+****-** |
|  | · · · · Nonproliferative retinopathy co-occurrent and due to type 2 diabetes mellitus (disorder) | ... |  | Y | **?****+****-** |
|  | · · · · · Mild nonproliferative retinopathy co-occurrent and due to type 2 diabetes mellitus (disorder) | ... |  | Y | **?****+****-** |
|  | · · · · · Moderate nonproliferative retinopathy co-occurrent and due to type 2 diabetes mellitus (disorder) | ... |  | Y | **?****+****-** |
|  | · · · · · Mild nonproliferative retinopathy co-occurrent and due to type 2 diabetes mellitus (disorder) | ... |  | Y | **?****+****-** |
|  | · · · · · Moderate nonproliferative retinopathy co-occurrent and due to type 2 diabetes mellitus (disorder) | ... |  | Y | **?****+****-** |
|  | · · · · · Mild nonproliferative retinopathy co-occurrent and due to type 2 diabetes mellitus (disorder) | ... |  | Y | **?****+****-** |
|  | · · · · · Moderate nonproliferative retinopathy co-occurrent and due to type 2 diabetes mellitus (disorder) | ... |  | Y | **?****+****-** |
|  | · · · · · Mild nonproliferative retinopathy co-occurrent and due to type 2 diabetes mellitus (disorder) | ... |  | Y | **?****+****-** |
|  | · · · · · Moderate nonproliferative retinopathy co-occurrent and due to type 2 diabetes mellitus (disorder) | ... |  | Y | **?****+****-** |
|  | · · · · · Mild nonproliferative retinopathy co-occurrent and due to type 2 diabetes mellitus (disorder) | ... |  | Y | **?****+****-** |
|  | · · · · · Moderate nonproliferative retinopathy co-occurrent and due to type 2 diabetes mellitus (disorder) | ... |  | Y | **?****+****-** |
|  | · · · · · Mild nonproliferative retinopathy co-occurrent and due to type 2 diabetes mellitus (disorder) | ... |  | Y | **?****+****-** |
|  | · · · · · Moderate nonproliferative retinopathy co-occurrent and due to type 2 diabetes mellitus (disorder) | ... |  | Y | **?****+****-** |
|  | · · · · · Mild nonproliferative retinopathy co-occurrent and due to type 2 diabetes mellitus (disorder) | ... |  | Y | **?****+****-** |
|  | · · · · · Moderate nonproliferative retinopathy co-occurrent and due to type 2 diabetes mellitus (disorder) | ... |  | Y | **?****+****-** |
|  | · · · · Proliferative retinopathy co-occurrent and due to type 2 diabetes mellitus (disorder) | ... |  | Y | **?****+****-** |
|  | · · · · Retinal edema co-occurrent and due to type 2 diabetes mellitus (disorder) | ... |  | Y | **?****+****-** |
|  | · · · · · Exudative maculopathy co-occurrent and due to type 2 diabetes mellitus (disorder) | ... |  | Y | **?****+****-** |
|  | · · · · · Macular edema and retinopathy due to type 2 diabetes mellitus (disorder) | ... |  | Y | **?****+****-** |
|  | · · · · · Proliferative retinopathy with retinal edema co-occurrent and due to type 2 diabetes mellitus (disorder) | ... |  | Y | **?****+****-** |
|  | · · · · · Exudative maculopathy co-occurrent and due to type 2 diabetes mellitus (disorder) | ... |  | Y | **?****+****-** |
|  | · · · · · Macular edema and retinopathy due to type 2 diabetes mellitus (disorder) | ... |  | Y | **?****+****-** |
|  | · · · · · Proliferative retinopathy with retinal edema co-occurrent and due to type 2 diabetes mellitus (disorder) | ... |  | Y | **?****+****-** |
|  | · · · · · Exudative maculopathy co-occurrent and due to type 2 diabetes mellitus (disorder) | ... |  | Y | **?****+****-** |
|  | · · · · · Macular edema and retinopathy due to type 2 diabetes mellitus (disorder) | ... |  | Y | **?****+****-** |
|  | · · · · · Exudative maculopathy co-occurrent and due to type 2 diabetes mellitus (disorder) | ... |  | Y | **?****+****-** |
|  | · · · · · Macular edema and retinopathy due to type 2 diabetes mellitus (disorder) | ... |  | Y | **?****+****-** |
|  | · · · · · Exudative maculopathy co-occurrent and due to type 2 diabetes mellitus (disorder) | ... |  | Y | **?****+****-** |
|  | · · · · · Macular edema and retinopathy due to type 2 diabetes mellitus (disorder) | ... |  | Y | **?****+****-** |
|  | · · · · · Exudative maculopathy co-occurrent and due to type 2 diabetes mellitus (disorder) | ... |  | Y | **?****+****-** |
|  | · · · · · Macular edema and retinopathy due to type 2 diabetes mellitus (disorder) | ... |  | Y | **?****+****-** |
|  | · · · · · Exudative maculopathy co-occurrent and due to type 2 diabetes mellitus (disorder) | ... |  | Y | **?****+****-** |
|  | · · · · · Macular edema and retinopathy due to type 2 diabetes mellitus (disorder) | ... |  | Y | **?****+****-** |
|  | · · · · · Exudative maculopathy co-occurrent and due to type 2 diabetes mellitus (disorder) | ... |  | Y | **?****+****-** |
|  | · · · · · Macular edema and retinopathy due to type 2 diabetes mellitus (disorder) | ... |  | Y | **?****+****-** |
|  | · · · · · Exudative maculopathy co-occurrent and due to type 2 diabetes mellitus (disorder) | ... |  | Y | **?****+****-** |
|  | · · · · · Macular edema and retinopathy due to type 2 diabetes mellitus (disorder) | ... |  | Y | **?****+****-** |
|  | · · · · · Exudative maculopathy co-occurrent and due to type 2 diabetes mellitus (disorder) | ... |  | Y | **?****+****-** |
|  | · · · · · Macular edema and retinopathy due to type 2 diabetes mellitus (disorder) | ... |  | Y | **?****+****-** |
|  | · · · · · Exudative maculopathy co-occurrent and due to type 2 diabetes mellitus (disorder) | ... |  | Y | **?****+****-** |
|  | · · · · · Macular edema and retinopathy due to type 2 diabetes mellitus (disorder) | ... |  | Y | **?****+****-** |
|  | · · · · · Proliferative retinopathy with retinal edema co-occurrent and due to type 2 diabetes mellitus (disorder) | ... |  | Y | **?****+****-** |
|  | · · · · Retinal ischemia co-occurrent and due to type 2 diabetes mellitus (disorder) | ... |  | Y | **?****+****-** |
|  | · · · · Traction retinal detachment co-occurrent and due to type 2 diabetes mellitus (disorder) | ... |  | Y | **?****+****-** |
|  | · Fetal hypertrophic cardiomyopathy due to maternal diabetes mellitus (disorder) | ... |  | N | **?****+****-** |
| Contract | · **Gangrene associated with diabetes mellitus (disorder)** | ... |  | Y | **?****+****-****++****--** |
|  | · · Gangrene associated with type I diabetes mellitus (disorder) | ... |  | Y | **?****+****-** |
|  | · · Gangrene associated with type II diabetes mellitus (disorder) | ... |  | Y | **?****+****-** |
| Contract | · **Glomerulopathy due to diabetes mellitus (disorder)** | ... |  | Y | **?****+****-****++****--** |
|  | · · Diabetic glomerulonephritis (disorder) | ... |  | Y | **?****+****-** |
| Contract | · · **Intracapillary glomerulosclerosis due to diabetes mellitus (disorder)** | ... |  | Y | **?****+****-****++****--** |
|  | · · · Diffuse type diabetic glomerulosclerosis (disorder) | ... |  | Y | **?****+****-** |
|  | · · · Diabetic glomerulosclerosis (disorder) | ... |  | Y | **?****+****-** |
|  | · · · · Nodular type diabetic glomerulosclerosis (disorder) | ... |  | Y | **?****+****-** |
| Contract | · · **Proteinuric nephropathy due to diabetes mellitus (disorder)** | ... |  | Y | **?****+****-****++****--** |
| Contract | · · · **Microalbuminuric nephropathy due to diabetes mellitus (disorder)** | ... |  | Y | **?****+****-****++****--** |
|  | · · · · Persistent microalbuminuria associated with type I diabetes mellitus (disorder) | ... |  | Y | **?****+****-** |
|  | · · · · Persistent microalbuminuria associated with type II diabetes mellitus (disorder) | ... |  | Y | **?****+****-** |
|  | · · · Persistent proteinuria associated with type I diabetes mellitus (disorder) | ... |  | Y | **?****+****-** |
|  | · · · Persistent proteinuria associated with type II diabetes mellitus (disorder) | ... |  | Y | **?****+****-** |
| Contract | · · · **Proteinuria due to type 1 diabetes mellitus (disorder)** | ... |  | Y | **?****+****-****++****--** |
|  | · · · · Microalbuminuria due to type 1 diabetes mellitus (disorder) | ... |  | Y | **?****+****-** |
| Contract | · · · **Proteinuria due to type 2 diabetes mellitus (disorder)** | ... |  | Y | **?****+****-****++****--** |
|  | · · · · Microalbuminuria due to type 2 diabetes mellitus (disorder) | ... |  | Y | **?****+****-** |
| Contract | · **Hyperosmolar coma due to diabetes mellitus (disorder)** | ... |  | Y | **?****+****-****++****--** |
|  | · · Hyperosmolar coma due to secondary diabetes mellitus (disorder) | ... |  | Y | **?****+****-** |
|  | · · Hyperosmolar hyperglycemic coma due to diabetes mellitus without ketoacidosis (disorder) | ... |  | Y | **?****+****-** |
| Contract | · **Hypoglycemic state due to diabetes mellitus (disorder)** | ... |  | Y | **?****+****-****++****--** |
|  | · · Hypoglycaemic warning absent (disorder) | ... |  | Y | **?****+****-** |
|  | · · Hypoglycaemic warning good (disorder) | ... |  | Y | **?****+****-** |
|  | · · Hypoglycaemic warning impaired (disorder) | ... |  | Y | **?****+****-** |
| Contract | · · **Loss of hypoglycemic warning (disorder)** | ... |  | Y | **?****+****-****++****--** |
|  | · · · Hypoglycemia unawareness in type 2 diabetes mellitus (disorder) | ... |  | Y | **?****+****-** |
|  | · · Nocturnal hypoglycemia (disorder) | ... |  | Y | **?****+****-** |
|  | · · Somogyi phenomenon (disorder) | ... |  | Y | **?****+****-** |
| Contract | · · **Hypoglycemia due to type 1 diabetes mellitus (disorder)** | ... |  | Y | **?****+****-****++****--** |
|  | · · · Hypoglycemic unawareness in type 1 diabetes mellitus (disorder) | ... |  | Y | **?****+****-** |
| Contract | · · **Hypoglycemia due to type 2 diabetes mellitus (disorder)** | ... |  | Y | **?****+****-****++****--** |
| Contract | · · · **Insulin reactive hypoglycemia in type 2 diabetes mellitus (disorder)** | ... |  | Y | **?****+****-****++****--** |
|  | · · · · Hypoglycemia unawareness in type 2 diabetes mellitus (disorder) | ... |  | Y | **?****+****-** |
| Contract | · **Infection of foot due to diabetes mellitus (disorder)** | ... |  | Y | **?****+****-****++****--** |
|  | · · Cellulitis in diabetic foot (disorder) | ... |  | Y | **?****+****-** |
| Contract | · **Lesion of skin co-occurrent and due to diabetes mellitus (disorder)** | ... |  | Y | **?****+****-****++****--** |
| Contract | · · **Dermopathy due to diabetes mellitus (disorder)** | ... |  | Y | **?****+****-****++****--** |
|  | · · · Diabetic rubeosis (disorder) | ... |  | Y | **?****+****-** |
|  | · · · Diabetic thick skin syndrome (disorder) | ... |  | Y | **?****+****-** |
|  | · · · Pretibial pigmental patches in diabetes (disorder) | ... |  | Y | **?****+****-** |
|  | · · · Diabetic dermopathy due to type 1 diabetes mellitus (disorder) | ... |  | Y | **?****+****-** |
| Contract | · · · **Diabetic dermopathy associated with diabetes mellitus type 2 (disorder)** | ... |  | Y | **?****+****-****++****--** |
|  | · · · · Type 2 diabetes mellitus with acanthosis nigricans (disorder) | ... |  | Y | **?****+****-** |
| Contract | · · **Skin ulcer associated with diabetes mellitus (disorder)** | ... |  | Y | **?****+****-****++****--** |
|  | · · · Chronic ulcer of skin due to type 1 diabetes mellitus (disorder) | ... |  | Y | **?****+****-** |
| Contract | · · · **Ulcer of lower limb due to type 1 diabetes mellitus (disorder)** | ... |  | Y | **?****+****-****++****--** |
|  | · · · · Skin ulcer of toe due to diabetes mellitus type 1 (disorder) | ... |  | Y | **?****+****-** |
| Contract | · · · **Ulcer of skin co-occurrent and due to type 2 diabetes mellitus (disorder)** | ... |  | Y | **?****+****-****++****--** |
|  | · · · · Stasis ulcer due to type 2 diabetes mellitus (disorder) | ... |  | Y | **?****+****-** |
| Contract | · **Multiple complications due to diabetes mellitus (disorder)** | ... |  | Y | **?****+****-****++****--** |
|  | · · Multiple complications of type I diabetes mellitus (disorder) | ... |  | Y | **?****+****-** |
|  | · Myasthenic syndrome due to diabetic amyotrophy (disorder) | ... |  | Y | **?****+****-** |
| Contract | · **Neuropathic arthropathy due to diabetes mellitus (disorder)** | ... |  | Y | **?****+****-****++****--** |
|  | · · Diabetic neuropathic arthropathy associated with type 2 diabetes mellitus (disorder) | ... |  | Y | **?****+****-** |
|  | · · Diabetic neuropathic arthropathy due to type 1 diabetes mellitus (disorder) | ... |  | Y | **?****+****-** |
|  | · Peripheral angiopathy due to diabetes mellitus (disorder) | ... |  | Y | **?****+****-** |
|  | · Acute complication co-occurrent and due to diabetes mellitus (disorder) | ... |  | Y | **?****+****-** |
|  | · Diabetic mastopathy (disorder) | ... |  | Y | **?****+****-** |
| Contract | · **Disorder of kidney co-occurrent and due to diabetes mellitus (disorder)** | ... |  | Y | **?****+****-****++****--** |
|  | · · Armanni-Ebstein kidney (disorder) | ... |  | Y | **?****+****-** |
|  | · · Kimmelstiel-Wilson syndrome (disorder) | ... |  | Y | **?****+****-** |
|  | · · Macroalbuminuric diabetic nephropathy (disorder) | ... |  | Y | **?****+****-** |
|  | · · Nephrotic syndrome due to diabetes mellitus (disorder) | ... |  | Y | **?****+****-** |
| Contract | · · **Renal disorder associated with type I diabetes mellitus (disorder)** | ... |  | Y | **?****+****-****++****--** |
|  | · · · Type 1 diabetes mellitus with persistent microalbuminuria (disorder) | ... |  | Y | **?****+****-** |
|  | · · Renal disorder due to type 2 diabetes mellitus (disorder) | ... |  | Y | **?****+****-** |
|  | · · Renal papillary necrosis due to diabetes mellitus (disorder) | ... |  | Y | **?****+****-** |
|  | · · Renal disorder due to type 2 diabetes mellitus (disorder) | ... |  | Y | **?****+****-** |
| Contract | · · **Nephrotic syndrome due to diabetes mellitus (disorder)** | ... |  | Y | **?****+****-****++****--** |
|  | · · · Nephrotic syndrome due to type 2 diabetes mellitus (disorder) | ... |  | Y | **?****+****-** |
|  | · · · Nephrotic syndrome due to type 1 diabetes mellitus (disorder) | ... |  | Y | **?****+****-** |
|  | · · · Nephrotic syndrome due to type 2 diabetes mellitus (disorder) | ... |  | Y | **?****+****-** |
| Contract | · **Disorder of nervous system co-occurrent and due to diabetes mellitus (disorder)** | ... |  | Y | **?****+****-****++****--** |
|  | · · Neurological disorder co-occurrent and due to type 1 diabetes mellitus (disorder) | ... |  | Y | **?****+****-** |
|  | · · Neurological disorder co-occurrent and due to type 2 diabetes mellitus (disorder) | ... |  | Y | **?****+****-** |
| Contract | · · **Coma associated with diabetes mellitus (disorder)** | ... |  | Y | **?****+****-****++****--** |
|  | · · · Type 1 diabetes mellitus with hyperosmolar coma (disorder) | ... |  | Y | **?****+****-** |
|  | · · · Type 2 diabetes mellitus with hyperosmolar coma (disorder) | ... |  | Y | **?****+****-** |
| Contract | · · · **Diabetic coma with ketoacidosis (disorder)** | ... |  | Y | **?****+****-****++****--** |
|  | · · · · Ketoacidotic coma in type I diabetes mellitus (disorder) | ... |  | Y | **?****+****-** |
|  | · · · · Ketoacidotic coma in type II diabetes mellitus (disorder) | ... |  | Y | **?****+****-** |
|  | · · · · Ketoacidotic coma in type I diabetes mellitus (disorder) | ... |  | Y | **?****+****-** |
|  | · · · · Ketoacidotic coma in type II diabetes mellitus (disorder) | ... |  | Y | **?****+****-** |
|  | · · · Non-ketotic non-hyperosmolar coma associated with diabetes mellitus (disorder) | ... |  | Y | **?****+****-** |
|  | · · · Type 1 diabetes mellitus with hyperosmolar coma (disorder) | ... |  | Y | **?****+****-** |
|  | · · · Type 2 diabetes mellitus with hyperosmolar coma (disorder) | ... |  | Y | **?****+****-** |
|  | · · · Hypoglycemic coma in diabetes mellitus (disorder) | ... |  | Y | **?****+****-** |
|  | · · · Type 1 diabetes mellitus with hyperosmolar coma (disorder) | ... |  | Y | **?****+****-** |
|  | · · · Type 2 diabetes mellitus with hyperosmolar coma (disorder) | ... |  | Y | **?****+****-** |
| Contract | · · · **Hypoglycemic coma in diabetes mellitus (disorder)** | ... |  | Y | **?****+****-****++****--** |
|  | · · · · Hypoglycemic coma in type I diabetes mellitus (disorder) | ... |  | Y | **?****+****-** |
| Contract | · · **Neurological disorder associated with malnutrition-related diabetes mellitus (disorder)** | ... |  | Y | **?****+****-****++****--** |
|  | · · · Coma associated with malnutrition-related diabetes mellitus (disorder) | ... |  | Y | **?****+****-** |
| Contract | · · **Neuropathy co-occurrent and due to diabetes mellitus (disorder)** | ... |  | Y | **?****+****-****++****--** |
|  | · · · Acute painful diabetic neuropathy (disorder) | ... |  | Y | **?****+****-** |
|  | · · · Asymptomatic neuropathy co-occurrent and due to diabetes mellitus (disorder) | ... |  | Y | **?****+****-** |
| Contract | · · · **Autonomic neuropathy co-occurrent and due to diabetes mellitus (disorder)** | ... |  | Y | **?****+****-****++****--** |
|  | · · · · Autonomic neuropathy co-occurrent and due to type 1 diabetes mellitus (disorder) | ... |  | Y | **?****+****-** |
|  | · · · · Diarrhea co-occurrent and due to diabetes mellitus (disorder) | ... |  | Y | **?****+****-** |
|  | · · · · Gastroparesis co-occurrent and due to diabetes mellitus (disorder) | ... |  | Y | **?****+****-** |
|  | · · · · Autonomic neuropathy co-occurrent and due to type 1 diabetes mellitus (disorder) | ... |  | Y | **?****+****-** |
|  | · · · · · Gastroparesis co-occurrent and due to type 1 diabetes mellitus (disorder) | ... |  | Y | **?****+****-** |
|  | · · · Chronic painful diabetic neuropathy (disorder) | ... |  | Y | **?****+****-** |
| Contract | · · · **Cranial nerve palsy co-occurrent and due to diabetes mellitus (disorder)** | ... |  | Y | **?****+****-****++****--** |
|  | · · · · Cranial nerve palsy co-occurrent and due to type 1 diabetes mellitus (disorder) | ... |  | Y | **?****+****-** |
|  | · · · Diabetic mononeuropathy multiplex (disorder) | ... |  | Y | **?****+****-** |
|  | · · · Diabetic neuropathy with neurologic complication (disorder) | ... |  | Y | **?****+****-** |
|  | · · · Peripheral neuropathy co-occurrent and due to diabetes mellitus (disorder) | ... |  | Y | **?****+****-** |
|  | · · · · Peripheral neuropathy co-occurrent and due to type 1 diabetes mellitus (disorder) | ... |  | Y | **?****+****-** |
|  | · · · · Asymmetric proximal motor neuropathy co-occurrent and due to diabetes mellitus (disorder) | ... |  | Y | **?****+****-** |
|  | · · · · Mononeuropathy co-occurrent and due to diabetes mellitus (disorder) | ... |  | Y | **?****+****-** |
|  | · · · · Ophthalmoplegia co-occurrent and due to diabetes mellitus (disorder) | ... |  | Y | **?****+****-** |
|  | · · · · Polyneuropathy co-occurrent and due to diabetes mellitus (disorder) | ... |  | Y | **?****+****-** |
|  | · · · · Pseudotabes co-occurrent and due to diabetes mellitus (disorder) | ... |  | Y | **?****+****-** |
|  | · · · · Radiculoplexus neuropathy co-occurrent and due to diabetes mellitus (disorder) | ... |  | Y | **?****+****-** |
|  | · · · · Symmetric proximal motor neuropathy co-occurrent and due to diabetes mellitus (disorder) | ... |  | Y | **?****+****-** |
|  | · · · · Symptomatic diabetic peripheral neuropathy (disorder) | ... |  | Y | **?****+****-** |
|  | · · · · Peripheral neuropathy co-occurrent and due to type 1 diabetes mellitus (disorder) | ... |  | Y | **?****+****-** |
|  | · · · · Asymmetric proximal motor neuropathy co-occurrent and due to diabetes mellitus (disorder) | ... |  | Y | **?****+****-** |
|  | · · · · Mononeuropathy co-occurrent and due to diabetes mellitus (disorder) | ... |  | Y | **?****+****-** |
|  | · · · · Ophthalmoplegia co-occurrent and due to diabetes mellitus (disorder) | ... |  | Y | **?****+****-** |
|  | · · · · Polyneuropathy co-occurrent and due to diabetes mellitus (disorder) | ... |  | Y | **?****+****-** |
|  | · · · · Pseudotabes co-occurrent and due to diabetes mellitus (disorder) | ... |  | Y | **?****+****-** |
|  | · · · · Radiculoplexus neuropathy co-occurrent and due to diabetes mellitus (disorder) | ... |  | Y | **?****+****-** |
|  | · · · · Symmetric proximal motor neuropathy co-occurrent and due to diabetes mellitus (disorder) | ... |  | Y | **?****+****-** |
|  | · · · · Symptomatic diabetic peripheral neuropathy (disorder) | ... |  | Y | **?****+****-** |
|  | · · · · Peripheral neuropathy co-occurrent and due to type 1 diabetes mellitus (disorder) | ... |  | Y | **?****+****-** |
|  | · · · · Asymmetric proximal motor neuropathy co-occurrent and due to diabetes mellitus (disorder) | ... |  | Y | **?****+****-** |
|  | · · · · Mononeuropathy co-occurrent and due to diabetes mellitus (disorder) | ... |  | Y | **?****+****-** |
|  | · · · · Ophthalmoplegia co-occurrent and due to diabetes mellitus (disorder) | ... |  | Y | **?****+****-** |
|  | · · · · Polyneuropathy co-occurrent and due to diabetes mellitus (disorder) | ... |  | Y | **?****+****-** |
|  | · · · · Pseudotabes co-occurrent and due to diabetes mellitus (disorder) | ... |  | Y | **?****+****-** |
|  | · · · · Radiculoplexus neuropathy co-occurrent and due to diabetes mellitus (disorder) | ... |  | Y | **?****+****-** |
|  | · · · · Symmetric proximal motor neuropathy co-occurrent and due to diabetes mellitus (disorder) | ... |  | Y | **?****+****-** |
|  | · · · · Symptomatic diabetic peripheral neuropathy (disorder) | ... |  | Y | **?****+****-** |
|  | · · · · Ophthalmoplegia co-occurrent and due to diabetes mellitus (disorder) | ... |  | Y | **?****+****-** |
|  | · · · · Peripheral neuropathy co-occurrent and due to type 1 diabetes mellitus (disorder) | ... |  | Y | **?****+****-** |
|  | · · · · Asymmetric proximal motor neuropathy co-occurrent and due to diabetes mellitus (disorder) | ... |  | Y | **?****+****-** |
| Contract | · · · · **Mononeuropathy co-occurrent and due to diabetes mellitus (disorder)** | ... |  | Y | **?****+****-****++****--** |
|  | · · · · · Femoral mononeuropathy co-occurrent and due to diabetes mellitus (disorder) | ... |  | Y | **?****+****-** |
|  | · · · · · Mononeuritis multiplex co-occurrent and due to diabetes mellitus (disorder) | ... |  | Y | **?****+****-** |
|  | · · · · · Mononeuropathy simplex co-occurrent and due to diabetes mellitus (disorder) | ... |  | Y | **?****+****-** |
|  | · · · · · Femoral mononeuropathy co-occurrent and due to diabetes mellitus (disorder) | ... |  | Y | **?****+****-** |
|  | · · · · · Mononeuritis multiplex co-occurrent and due to diabetes mellitus (disorder) | ... |  | Y | **?****+****-** |
|  | · · · · · Mononeuropathy simplex co-occurrent and due to diabetes mellitus (disorder) | ... |  | Y | **?****+****-** |
|  | · · · · · Femoral mononeuropathy co-occurrent and due to diabetes mellitus (disorder) | ... |  | Y | **?****+****-** |
|  | · · · · · Mononeuritis multiplex co-occurrent and due to diabetes mellitus (disorder) | ... |  | Y | **?****+****-** |
|  | · · · · · Mononeuropathy simplex co-occurrent and due to diabetes mellitus (disorder) | ... |  | Y | **?****+****-** |
|  | · · · · · Femoral mononeuropathy co-occurrent and due to diabetes mellitus (disorder) | ... |  | Y | **?****+****-** |
|  | · · · · · Mononeuritis multiplex co-occurrent and due to diabetes mellitus (disorder) | ... |  | Y | **?****+****-** |
|  | · · · · · Mononeuropathy simplex co-occurrent and due to diabetes mellitus (disorder) | ... |  | Y | **?****+****-** |
|  | · · · · · Femoral mononeuropathy co-occurrent and due to diabetes mellitus (disorder) | ... |  | Y | **?****+****-** |
|  | · · · · · Mononeuritis multiplex co-occurrent and due to diabetes mellitus (disorder) | ... |  | Y | **?****+****-** |
|  | · · · · · Mononeuropathy simplex co-occurrent and due to diabetes mellitus (disorder) | ... |  | Y | **?****+****-** |
| Contract | · · · · **Polyneuropathy co-occurrent and due to diabetes mellitus (disorder)** | ... |  | Y | **?****+****-****++****--** |
|  | · · · · · Acute painful polyneuropathy co-occurrent and due to diabetes mellitus (disorder) | ... |  | Y | **?****+****-** |
|  | · · · · · Chronic painful polyneuropathy co-occurrent and due to diabetes mellitus (disorder) | ... |  | Y | **?****+****-** |
|  | · · · · · Asymmetric polyneuropathy co-occurrent and due to diabetes mellitus (disorder) | ... |  | Y | **?****+****-** |
|  | · · · · · Motor polyneuropathy co-occurrent and due to diabetes mellitus (disorder) | ... |  | Y | **?****+****-** |
|  | · · · · · Sensory neuropathy co-occurrent and due to diabetes mellitus (disorder) | ... |  | Y | **?****+****-** |
|  | · · · · · Acute painful polyneuropathy co-occurrent and due to diabetes mellitus (disorder) | ... |  | Y | **?****+****-** |
|  | · · · · · Chronic painful polyneuropathy co-occurrent and due to diabetes mellitus (disorder) | ... |  | Y | **?****+****-** |
|  | · · · · · Asymmetric polyneuropathy co-occurrent and due to diabetes mellitus (disorder) | ... |  | Y | **?****+****-** |
|  | · · · · · Motor polyneuropathy co-occurrent and due to diabetes mellitus (disorder) | ... |  | Y | **?****+****-** |
|  | · · · · · Sensory neuropathy co-occurrent and due to diabetes mellitus (disorder) | ... |  | Y | **?****+****-** |
|  | · · · · · Acute painful polyneuropathy co-occurrent and due to diabetes mellitus (disorder) | ... |  | Y | **?****+****-** |
|  | · · · · · Chronic painful polyneuropathy co-occurrent and due to diabetes mellitus (disorder) | ... |  | Y | **?****+****-** |
|  | · · · · · Asymmetric polyneuropathy co-occurrent and due to diabetes mellitus (disorder) | ... |  | Y | **?****+****-** |
|  | · · · · · Motor polyneuropathy co-occurrent and due to diabetes mellitus (disorder) | ... |  | Y | **?****+****-** |
|  | · · · · · Sensory neuropathy co-occurrent and due to diabetes mellitus (disorder) | ... |  | Y | **?****+****-** |
|  | · · · · · Acute painful polyneuropathy co-occurrent and due to diabetes mellitus (disorder) | ... |  | Y | **?****+****-** |
|  | · · · · · Chronic painful polyneuropathy co-occurrent and due to diabetes mellitus (disorder) | ... |  | Y | **?****+****-** |
|  | · · · · · Asymmetric polyneuropathy co-occurrent and due to diabetes mellitus (disorder) | ... |  | Y | **?****+****-** |
|  | · · · · · Motor polyneuropathy co-occurrent and due to diabetes mellitus (disorder) | ... |  | Y | **?****+****-** |
|  | · · · · · Sensory neuropathy co-occurrent and due to diabetes mellitus (disorder) | ... |  | Y | **?****+****-** |
|  | · · · · · Acute painful polyneuropathy co-occurrent and due to diabetes mellitus (disorder) | ... |  | Y | **?****+****-** |
|  | · · · · · Chronic painful polyneuropathy co-occurrent and due to diabetes mellitus (disorder) | ... |  | Y | **?****+****-** |
|  | · · · · · Asymmetric polyneuropathy co-occurrent and due to diabetes mellitus (disorder) | ... |  | Y | **?****+****-** |
|  | · · · · · Motor polyneuropathy co-occurrent and due to diabetes mellitus (disorder) | ... |  | Y | **?****+****-** |
| Contract | · · · · · **Sensory neuropathy co-occurrent and due to diabetes mellitus (disorder)** | ... |  | Y | **?****+****-****++****--** |
|  | · · · · · · Mixed sensorimotor polyneuropathy co-occurrent and due to diabetes mellitus (disorder) | ... |  | Y | **?****+****-** |
|  | · · · · · Acute painful polyneuropathy co-occurrent and due to diabetes mellitus (disorder) | ... |  | Y | **?****+****-** |
|  | · · · · · Chronic painful polyneuropathy co-occurrent and due to diabetes mellitus (disorder) | ... |  | Y | **?****+****-** |
|  | · · · · Pseudotabes co-occurrent and due to diabetes mellitus (disorder) | ... |  | Y | **?****+****-** |
| Contract | · · · · **Radiculoplexus neuropathy co-occurrent and due to diabetes mellitus (disorder)** | ... |  | Y | **?****+****-****++****--** |
|  | · · · · · Cervical radiculoplexus neuropathy co-occurrent and due to diabetes mellitus (disorder) | ... |  | Y | **?****+****-** |
|  | · · · · · Lumbosacral radiculoplexus neuropathy co-occurrent and due to diabetes mellitus (disorder) | ... |  | Y | **?****+****-** |
|  | · · · · · Thoracic radiculopathy co-occurrent and due to diabetes mellitus (disorder) | ... |  | Y | **?****+****-** |
|  | · · · · · Cervical radiculoplexus neuropathy co-occurrent and due to diabetes mellitus (disorder) | ... |  | Y | **?****+****-** |
|  | · · · · · Lumbosacral radiculoplexus neuropathy co-occurrent and due to diabetes mellitus (disorder) | ... |  | Y | **?****+****-** |
|  | · · · · · Thoracic radiculopathy co-occurrent and due to diabetes mellitus (disorder) | ... |  | Y | **?****+****-** |
|  | · · · · · Cervical radiculoplexus neuropathy co-occurrent and due to diabetes mellitus (disorder) | ... |  | Y | **?****+****-** |
|  | · · · · · Lumbosacral radiculoplexus neuropathy co-occurrent and due to diabetes mellitus (disorder) | ... |  | Y | **?****+****-** |
|  | · · · · · Thoracic radiculopathy co-occurrent and due to diabetes mellitus (disorder) | ... |  | Y | **?****+****-** |
|  | · · · · · Cervical radiculoplexus neuropathy co-occurrent and due to diabetes mellitus (disorder) | ... |  | Y | **?****+****-** |
|  | · · · · · Lumbosacral radiculoplexus neuropathy co-occurrent and due to diabetes mellitus (disorder) | ... |  | Y | **?****+****-** |
|  | · · · · · Thoracic radiculopathy co-occurrent and due to diabetes mellitus (disorder) | ... |  | Y | **?****+****-** |
|  | · · · · · Cervical radiculoplexus neuropathy co-occurrent and due to diabetes mellitus (disorder) | ... |  | Y | **?****+****-** |
| Contract | · · · · · **Lumbosacral radiculoplexus neuropathy co-occurrent and due to diabetes mellitus (disorder)** | ... |  | Y | **?****+****-****++****--** |
|  | · · · · · · Lumbosacral radiculoplexus neuropathy co-occurrent and due to type 1 diabetes mellitus (disorder) | ... |  | Y | **?****+****-** |
|  | · · · · · · Lumbosacral radiculoplexus neuropathy co-occurrent and due to type 2 diabetes mellitus (disorder) | ... |  | Y | **?****+****-** |
|  | · · · · · · Lumbosacral radiculoplexus neuropathy co-occurrent and due to type 1 diabetes mellitus (disorder) | ... |  | Y | **?****+****-** |
|  | · · · · · · Lumbosacral radiculoplexus neuropathy co-occurrent and due to type 2 diabetes mellitus (disorder) | ... |  | Y | **?****+****-** |
|  | · · · · · · Lumbosacral radiculoplexus neuropathy co-occurrent and due to type 1 diabetes mellitus (disorder) | ... |  | Y | **?****+****-** |
|  | · · · · · · Lumbosacral radiculoplexus neuropathy co-occurrent and due to type 2 diabetes mellitus (disorder) | ... |  | Y | **?****+****-** |
|  | · · · · · · Lumbosacral radiculoplexus neuropathy co-occurrent and due to type 1 diabetes mellitus (disorder) | ... |  | Y | **?****+****-** |
|  | · · · · · · Lumbosacral radiculoplexus neuropathy co-occurrent and due to type 2 diabetes mellitus (disorder) | ... |  | Y | **?****+****-** |
|  | · · · · · · Lumbosacral radiculoplexus neuropathy co-occurrent and due to type 1 diabetes mellitus (disorder) | ... |  | Y | **?****+****-** |
|  | · · · · · · Lumbosacral radiculoplexus neuropathy co-occurrent and due to type 2 diabetes mellitus (disorder) | ... |  | Y | **?****+****-** |
|  | · · · · · · Lumbosacral radiculoplexus neuropathy co-occurrent and due to type 1 diabetes mellitus (disorder) | ... |  | Y | **?****+****-** |
|  | · · · · · · Lumbosacral radiculoplexus neuropathy co-occurrent and due to type 2 diabetes mellitus (disorder) | ... |  | Y | **?****+****-** |
|  | · · · · · Thoracic radiculopathy co-occurrent and due to diabetes mellitus (disorder) | ... |  | Y | **?****+****-** |
|  | · · · · Symmetric proximal motor neuropathy co-occurrent and due to diabetes mellitus (disorder) | ... |  | Y | **?****+****-** |
|  | · · · · Symptomatic diabetic peripheral neuropathy (disorder) | ... |  | Y | **?****+****-** |
|  | · · · · Peripheral neuropathy co-occurrent and due to type 1 diabetes mellitus (disorder) | ... |  | Y | **?****+****-** |
|  | · · · · · Mononeuropathy co-occurrent and due to type 1 diabetes mellitus (disorder) | ... |  | Y | **?****+****-** |
|  | · · · · · Polyneuropathy co-occurrent and due to type 1 diabetes mellitus (disorder) | ... |  | Y | **?****+****-** |
|  | · · · · · Sensory neuropathy due to type 1 diabetes mellitus (disorder) | ... |  | Y | **?****+****-** |
|  | · · · · · Mononeuropathy co-occurrent and due to type 1 diabetes mellitus (disorder) | ... |  | Y | **?****+****-** |
|  | · · · · · Polyneuropathy co-occurrent and due to type 1 diabetes mellitus (disorder) | ... |  | Y | **?****+****-** |
|  | · · · · · Sensory neuropathy due to type 1 diabetes mellitus (disorder) | ... |  | Y | **?****+****-** |
|  | · · · · · Mononeuropathy co-occurrent and due to type 1 diabetes mellitus (disorder) | ... |  | Y | **?****+****-** |
|  | · · · · · Polyneuropathy co-occurrent and due to type 1 diabetes mellitus (disorder) | ... |  | Y | **?****+****-** |
|  | · · · · · Sensory neuropathy due to type 1 diabetes mellitus (disorder) | ... |  | Y | **?****+****-** |
|  | · · · · · Mononeuropathy co-occurrent and due to type 1 diabetes mellitus (disorder) | ... |  | Y | **?****+****-** |
|  | · · · · · Polyneuropathy co-occurrent and due to type 1 diabetes mellitus (disorder) | ... |  | Y | **?****+****-** |
|  | · · · · · Sensory neuropathy due to type 1 diabetes mellitus (disorder) | ... |  | Y | **?****+****-** |
|  | · · · · · Mononeuropathy co-occurrent and due to type 1 diabetes mellitus (disorder) | ... |  | Y | **?****+****-** |
|  | · · · · · Polyneuropathy co-occurrent and due to type 1 diabetes mellitus (disorder) | ... |  | Y | **?****+****-** |
|  | · · · · · Sensory neuropathy due to type 1 diabetes mellitus (disorder) | ... |  | Y | **?****+****-** |
|  | · · · · · Mononeuropathy co-occurrent and due to type 1 diabetes mellitus (disorder) | ... |  | Y | **?****+****-** |
|  | · · · · · Polyneuropathy co-occurrent and due to type 1 diabetes mellitus (disorder) | ... |  | Y | **?****+****-** |
|  | · · · · · Sensory neuropathy due to type 1 diabetes mellitus (disorder) | ... |  | Y | **?****+****-** |
|  | · · · · · Mononeuropathy co-occurrent and due to type 1 diabetes mellitus (disorder) | ... |  | Y | **?****+****-** |
|  | · · · · · Polyneuropathy co-occurrent and due to type 1 diabetes mellitus (disorder) | ... |  | Y | **?****+****-** |
|  | · · Neurological disorder co-occurrent and due to type 1 diabetes mellitus (disorder) | ... |  | Y | **?****+****-** |
|  | · · Neurological disorder co-occurrent and due to type 2 diabetes mellitus (disorder) | ... |  | Y | **?****+****-** |
|  | · · · Hypoglycemic coma co-occurrent and due to diabetes mellitus type II (disorder) | ... |  | Y | **?****+****-** |
|  | · · · Neuropathy due to type 2 diabetes mellitus (disorder) | ... |  | Y | **?****+****-** |
|  | · · · Hypoglycemic coma co-occurrent and due to diabetes mellitus type II (disorder) | ... |  | Y | **?****+****-** |
|  | · · · Neuropathy due to type 2 diabetes mellitus (disorder) | ... |  | Y | **?****+****-** |
|  | · · · Hypoglycemic coma co-occurrent and due to diabetes mellitus type II (disorder) | ... |  | Y | **?****+****-** |
|  | · · · Neuropathy due to type 2 diabetes mellitus (disorder) | ... |  | Y | **?****+****-** |
|  | · · · · Autonomic neuropathy co-occurrent and due to type 2 diabetes mellitus (disorder) | ... |  | Y | **?****+****-** |
|  | · · · · Cranial nerve palsy co-occurrent and due to type 2 diabetes mellitus (disorder) | ... |  | Y | **?****+****-** |
|  | · · · · Peripheral neuropathy co-occurrent and due to type 2 diabetes mellitus (disorder) | ... |  | Y | **?****+****-** |
|  | · · · · Autonomic neuropathy co-occurrent and due to type 2 diabetes mellitus (disorder) | ... |  | Y | **?****+****-** |
|  | · · · · Cranial nerve palsy co-occurrent and due to type 2 diabetes mellitus (disorder) | ... |  | Y | **?****+****-** |
|  | · · · · Peripheral neuropathy co-occurrent and due to type 2 diabetes mellitus (disorder) | ... |  | Y | **?****+****-** |
|  | · · · · Autonomic neuropathy co-occurrent and due to type 2 diabetes mellitus (disorder) | ... |  | Y | **?****+****-** |
|  | · · · · Cranial nerve palsy co-occurrent and due to type 2 diabetes mellitus (disorder) | ... |  | Y | **?****+****-** |
|  | · · · · Peripheral neuropathy co-occurrent and due to type 2 diabetes mellitus (disorder) | ... |  | Y | **?****+****-** |
|  | · · · · Autonomic neuropathy co-occurrent and due to type 2 diabetes mellitus (disorder) | ... |  | Y | **?****+****-** |
|  | · · · · Cranial nerve palsy co-occurrent and due to type 2 diabetes mellitus (disorder) | ... |  | Y | **?****+****-** |
|  | · · · · Peripheral neuropathy co-occurrent and due to type 2 diabetes mellitus (disorder) | ... |  | Y | **?****+****-** |
|  | · · · · Autonomic neuropathy co-occurrent and due to type 2 diabetes mellitus (disorder) | ... |  | Y | **?****+****-** |
|  | · · · · Cranial nerve palsy co-occurrent and due to type 2 diabetes mellitus (disorder) | ... |  | Y | **?****+****-** |
|  | · · · · Peripheral neuropathy co-occurrent and due to type 2 diabetes mellitus (disorder) | ... |  | Y | **?****+****-** |
|  | · · · · Autonomic neuropathy co-occurrent and due to type 2 diabetes mellitus (disorder) | ... |  | Y | **?****+****-** |
|  | · · · · · Gastroparesis co-occurrent and due to type 2 diabetes mellitus (disorder) | ... |  | Y | **?****+****-** |
|  | · · · · Cranial nerve palsy co-occurrent and due to type 2 diabetes mellitus (disorder) | ... |  | Y | **?****+****-** |
|  | · · · · Peripheral neuropathy co-occurrent and due to type 2 diabetes mellitus (disorder) | ... |  | Y | **?****+****-** |
|  | · · · · · Mononeuropathy co-occurrent and due to type 2 diabetes mellitus (disorder) | ... |  | Y | **?****+****-** |
|  | · · · · · Peripheral sensory neuropathy due to type 2 diabetes mellitus (disorder) | ... |  | Y | **?****+****-** |
|  | · · · · · Polyneuropathy co-occurrent and due to type 2 diabetes mellitus (disorder) | ... |  | Y | **?****+****-** |
|  | · · · · · Mononeuropathy co-occurrent and due to type 2 diabetes mellitus (disorder) | ... |  | Y | **?****+****-** |
|  | · · · · · Peripheral sensory neuropathy due to type 2 diabetes mellitus (disorder) | ... |  | Y | **?****+****-** |
|  | · · · · · Polyneuropathy co-occurrent and due to type 2 diabetes mellitus (disorder) | ... |  | Y | **?****+****-** |
|  | · · · · · Mononeuropathy co-occurrent and due to type 2 diabetes mellitus (disorder) | ... |  | Y | **?****+****-** |
|  | · · · · · Peripheral sensory neuropathy due to type 2 diabetes mellitus (disorder) | ... |  | Y | **?****+****-** |
|  | · · · · · Polyneuropathy co-occurrent and due to type 2 diabetes mellitus (disorder) | ... |  | Y | **?****+****-** |
|  | · · · · · Mononeuropathy co-occurrent and due to type 2 diabetes mellitus (disorder) | ... |  | Y | **?****+****-** |
|  | · · · · · Peripheral sensory neuropathy due to type 2 diabetes mellitus (disorder) | ... |  | Y | **?****+****-** |
|  | · · · · · Polyneuropathy co-occurrent and due to type 2 diabetes mellitus (disorder) | ... |  | Y | **?****+****-** |
|  | · · · · · Mononeuropathy co-occurrent and due to type 2 diabetes mellitus (disorder) | ... |  | Y | **?****+****-** |
|  | · · · · · Peripheral sensory neuropathy due to type 2 diabetes mellitus (disorder) | ... |  | Y | **?****+****-** |
|  | · · · · · Polyneuropathy co-occurrent and due to type 2 diabetes mellitus (disorder) | ... |  | Y | **?****+****-** |
|  | · · · · · Mononeuropathy co-occurrent and due to type 2 diabetes mellitus (disorder) | ... |  | Y | **?****+****-** |
|  | · · · · · Peripheral sensory neuropathy due to type 2 diabetes mellitus (disorder) | ... |  | Y | **?****+****-** |
|  | · · · · · Polyneuropathy co-occurrent and due to type 2 diabetes mellitus (disorder) | ... |  | Y | **?****+****-** |
|  | · · · · · Mononeuropathy co-occurrent and due to type 2 diabetes mellitus (disorder) | ... |  | Y | **?****+****-** |
|  | · · · · · Peripheral sensory neuropathy due to type 2 diabetes mellitus (disorder) | ... |  | Y | **?****+****-** |
|  | · · · · · Polyneuropathy co-occurrent and due to type 2 diabetes mellitus (disorder) | ... |  | Y | **?****+****-** |
|  | · · · · · Mononeuropathy co-occurrent and due to type 2 diabetes mellitus (disorder) | ... |  | Y | **?****+****-** |
|  | · · · · · Peripheral sensory neuropathy due to type 2 diabetes mellitus (disorder) | ... |  | Y | **?****+****-** |
|  | · · · · · Polyneuropathy co-occurrent and due to type 2 diabetes mellitus (disorder) | ... |  | Y | **?****+****-** |
|  | · · · · · Mononeuropathy co-occurrent and due to type 2 diabetes mellitus (disorder) | ... |  | Y | **?****+****-** |
|  | · · · · · Peripheral sensory neuropathy due to type 2 diabetes mellitus (disorder) | ... |  | Y | **?****+****-** |
|  | · · · · · Polyneuropathy co-occurrent and due to type 2 diabetes mellitus (disorder) | ... |  | Y | **?****+****-** |
|  | · · · · · Mononeuropathy co-occurrent and due to type 2 diabetes mellitus (disorder) | ... |  | Y | **?****+****-** |
|  | · · · · · Peripheral sensory neuropathy due to type 2 diabetes mellitus (disorder) | ... |  | Y | **?****+****-** |
|  | · · · · · Polyneuropathy co-occurrent and due to type 2 diabetes mellitus (disorder) | ... |  | Y | **?****+****-** |
|  | · · · · · Peripheral sensory neuropathy due to type 2 diabetes mellitus (disorder) | ... |  | Y | **?****+****-** |
|  | · · · · · Mononeuropathy co-occurrent and due to type 2 diabetes mellitus (disorder) | ... |  | Y | **?****+****-** |
|  | · · · · · Polyneuropathy co-occurrent and due to type 2 diabetes mellitus (disorder) | ... |  | Y | **?****+****-** |
| Contract | · **Disorder of soft tissue co-occurrent and due to diabetes mellitus (disorder)** | ... |  | Y | **?****+****-****++****--** |
|  | · · Bullosis diabeticorum (disorder) | ... |  | Y | **?****+****-** |
| Contract | · **Erectile dysfunction co-occurrent and due to diabetes mellitus (disorder)** | ... |  | Y | **?****+****-****++****--** |
|  | · · Erectile dysfunction co-occurrent and due to type 1 diabetes mellitus (disorder) | ... |  | Y | **?****+****-** |
|  | · · Erectile dysfunction co-occurrent and due to type 2 diabetes mellitus (disorder) | ... |  | Y | **?****+****-** |
|  | · · Erectile dysfunction co-occurrent and due to type 1 diabetes mellitus (disorder) | ... |  | Y | **?****+****-** |
|  | · · Erectile dysfunction co-occurrent and due to type 2 diabetes mellitus (disorder) | ... |  | Y | **?****+****-** |
| Contract | · **Peripheral vascular disorder co-occurrent and due to diabetes mellitus (disorder)** | ... |  | Y | **?****+****-****++****--** |
| Contract | · · **Peripheral circulatory disorder associated with type I diabetes mellitus (disorder)** | ... |  | Y | **?****+****-****++****--** |
|  | · · · Peripheral angiopathy due to type 1 diabetes mellitus (disorder) | ... |  | Y | **?****+****-** |
| Contract | · · **Peripheral circulatory disorder associated with type II diabetes mellitus (disorder)** | ... |  | Y | **?****+****-****++****--** |
|  | · · · Type 2 diabetes mellitus with peripheral angiopathy (disorder) | ... |  | Y | **?****+****-** |
|  | History of admission in last year for diabetes foot problem (situation) | ... |  | Y | **?****+****-** |
| Contract | **History of diabetes mellitus (situation)** | ... |  | Y | **?****+****-****++****--** |
|  | · History of diabetes mellitus type 1 (situation) | ... |  | Y | **?****+****-** |
|  | · History of diabetes mellitus type 2 (situation) | ... |  | Y | **?****+****-** |
|  | · History of gestational diabetes mellitus (situation) | ... |  | N | **?****+****-** |
|  | · History of maturity onset diabetes mellitus in young (situation) | ... |  | Y | **?****+****-** |
|  | · History of secondary diabetes mellitus (situation) | ... |  | Y | **?****+****-** |
|  | History of diabetic foot ulcer (situation) | ... |  | Y | **?****+****-** |
|  | History of diabetic ketoacidosis (situation) | ... |  | Y | **?****+****-** |
|  | History of diabetic peripheral angiopathy (situation) | ... |  | Y | **?****+****-** |
|  | History of nocturnal hypoglycemia (situation) | ... |  | Y | **?****+****-** |
|  | Hyperosmolarity due to type 1 diabetes mellitus (disorder) | ... |  | Y | **?****+****-** |
|  | On examination - diabetic maculopathy present both eyes (situation) | ... |  | Y | **?****+****-** |

·
