# Supplementary material for: Translating and evaluating historic phenotyping algorithms using SNOMED CT
Source: J Am Med Inform Assoc. 2022 Sep 9;30(2):222–32. doi: 10.1093/jamia/ocac158 (PMC9846670; doi:10.1093/jamia/ocac158)
Supplement: ocac158_Supplementary_Data [file ocac158_supplementary_data.zip › ocac158_Supplementary_Data/Supp_3_asthma_ext.html]

Extended SNOMED CT codelist for asthma

# Extended SNOMED CT codelist for asthma

## Instructions

This HTML document presents a hierarchy of SNOMED CT concepts.
In SNOMED CT, each concept has a distinct meaning and can
be linked to more general terms (ancestors) and more specific terms (descendants). The buttons allow you to explore the codelist at
different levels of the hierarchy, and mark whether or not you agree
with the inclusion of individual concepts or concept hierarchies. When
you have finished your review, you can download your final selection as a .CSV file by clicking the **Export** button below.

### Key to buttons for each concept

- Expand Show descendants of this concept
- Contract Hide descendants of this concept
- **?** Mark as unchecked
- **+** Add a concept
- **-** Remove a concept
- **++** Add a concept and all descendants
- **--** Remove a concept and all descendants

## Reviewing tools

Show top-level concepts only Show all concepts

**Mark all concepts as "checked"** **Mark all concepts as "unchecked"** Show unchecked concepts only

**Export** to
 .csv

| Expand | SNOMED CT concept | Comment | Checked | Included |  |
| --- | --- | --- | --- | --- | --- |
| Contract | **Asthma (disorder)** | ... |  | Y | **?****+****-****++****--** |
| Contract | · **Acute asthma (disorder)** | ... |  | Y | **?****+****-****++****--** |
| Contract | · · **Acute exacerbation of asthma (disorder)** | ... |  | Y | **?****+****-****++****--** |
| Contract | · · · **Acute exacerbation of asthma co-occurrent with allergic rhinitis (disorder)** | ... |  | Y | **?****+****-****++****--** |
|  | · · · · Acute severe exacerbation of asthma co-occurrent with allergic rhinitis (disorder) | ... |  | Y | **?****+****-** |
| Contract | · · · **Acute exacerbation of immunoglobulin E-mediated allergic asthma (disorder)** | ... |  | Y | **?****+****-****++****--** |
|  | · · · · Acute severe exacerbation of immunoglobin E-mediated allergic asthma (disorder) | ... |  | Y | **?****+****-** |
| Contract | · · · **Acute exacerbation of mild persistent asthma (disorder)** | ... |  | Y | **?****+****-****++****--** |
|  | · · · · Acute severe exacerbation of mild persistent asthma (disorder) | ... |  | Y | **?****+****-** |
| Contract | · · · **Acute exacerbation of moderate persistent asthma (disorder)** | ... |  | Y | **?****+****-****++****--** |
|  | · · · · Acute severe exacerbation of moderate persistent asthma (disorder) | ... |  | Y | **?****+****-** |
| Contract | · · · **Acute severe exacerbation of asthma (disorder)** | ... |  | Y | **?****+****-****++****--** |
|  | · · · · Acute severe refractory exacerbation of asthma (disorder) | ... |  | Y | **?****+****-** |
|  | · · · · Life threatening acute exacerbation of asthma (disorder) | ... |  | Y | **?****+****-** |
|  | · · · Chronic obstructive asthma co-occurrent with acute exacerbation of asthma (disorder) | ... |  | Y | **?****+****-** |
|  | · · · Moderate acute exacerbation of asthma (disorder) | ... |  | Y | **?****+****-** |
|  | · · Acute exacerbation of chronic asthmatic bronchitis (disorder) | ... |  | Y | **?****+****-** |
|  | · · Acute exacerbation of chronic obstructive airways disease with asthma (disorder) | ... |  | Y | **?****+****-** |
|  | · · Near fatal asthma (disorder) | ... |  | Y | **?****+****-** |
| Contract | · **Allergic asthma (disorder)** | ... |  | Y | **?****+****-****++****--** |
|  | · · Allergic asthma caused by Dermatophagoides farinae (disorder) | ... |  | Y | **?****+****-** |
|  | · · Allergic asthma caused by Dermatophagoides pteronyssinus (disorder) | ... |  | Y | **?****+****-** |
|  | · · Asthma caused by wood dust (disorder) | ... |  | Y | **?****+****-** |
|  | · · Colophony asthma (disorder) | ... |  | Y | **?****+****-** |
| Contract | · · **Exacerbation of allergic asthma (disorder)** | ... |  | Y | **?****+****-****++****--** |
| Contract | · · · **Acute severe exacerbation of allergic asthma (disorder)** | ... |  | Y | **?****+****-****++****--** |
|  | · · · · Life threatening acute exacerbation of allergic asthma (disorder) | ... |  | Y | **?****+****-** |
|  | · · · Exacerbation of allergic asthma due to infection (disorder) | ... |  | Y | **?****+****-** |
| Contract | · · **Immunoglobulin E-mediated allergic asthma (disorder)** | ... |  | Y | **?****+****-****++****--** |
|  | · · · Allergic asthma without status asthmaticus (disorder) | ... |  | Y | **?****+****-** |
| Contract | · · **Mild persistent allergic asthma (disorder)** | ... |  | Y | **?****+****-****++****--** |
|  | · · · Acute severe exacerbation of mild persistent allergic asthma (disorder) | ... |  | Y | **?****+****-** |
| Contract | · · **Moderate persistent allergic asthma (disorder)** | ... |  | Y | **?****+****-****++****--** |
|  | · · · Acute severe exacerbation of moderate persistent allergic asthma (disorder) | ... |  | Y | **?****+****-** |
|  | · · Non-immunoglobulin E mediated allergic asthma (disorder) | ... |  | Y | **?****+****-** |
| Contract | · · **Severe persistent allergic asthma (disorder)** | ... |  | Y | **?****+****-****++****--** |
|  | · · · Acute severe exacerbation of severe persistent allergic asthma (disorder) | ... |  | Y | **?****+****-** |
|  | · · Uncomplicated allergic asthma (disorder) | ... |  | Y | **?****+****-** |
|  | · Asthma in mother complicating childbirth (disorder) | ... |  | Y | **?****+****-** |
|  | · Asthma in pregnancy (disorder) | ... |  | Y | **?****+****-** |
|  | · Asthma with irreversible airway obstruction (disorder) | ... |  | Y | **?****+****-** |
| Contract | · **Asthma without status asthmaticus (disorder)** | ... |  | Y | **?****+****-****++****--** |
|  | · · Allergic asthma without status asthmaticus (disorder) | ... |  | Y | **?****+****-** |
|  | · · Intrinsic asthma without status asthmaticus (disorder) | ... |  | Y | **?****+****-** |
|  | · Asthma-chronic obstructive pulmonary disease overlap syndrome (disorder) | ... |  | Y | **?****+****-** |
| Contract | · **Asthmatic bronchitis (disorder)** | ... |  | Y | **?****+****-****++****--** |
| Contract | · · **Chronic asthmatic bronchitis (disorder)** | ... |  | Y | **?****+****-****++****--** |
|  | · · · Acute exacerbation of chronic asthmatic bronchitis (disorder) | ... |  | Y | **?****+****-** |
|  | · Brittle asthma (disorder) | ... |  | Y | **?****+****-** |
|  | · Childhood asthma (disorder) | ... |  | Y | **?****+****-** |
|  | · Chronic asthma with fixed airflow obstruction (disorder) | ... |  | Y | **?****+****-** |
|  | · Cough variant asthma (disorder) | ... |  | Y | **?****+****-** |
| Contract | · **Exacerbation of asthma (disorder)** | ... |  | Y | **?****+****-****++****--** |
|  | · · Exacerbation of intermittent asthma (disorder) | ... |  | Y | **?****+****-** |
|  | · · Exacerbation of mild persistent asthma (disorder) | ... |  | Y | **?****+****-** |
|  | · · Exacerbation of moderate persistent asthma (disorder) | ... |  | Y | **?****+****-** |
| Contract | · · **Exacerbation of severe persistent asthma (disorder)** | ... |  | Y | **?****+****-****++****--** |
| Contract | · · · **Acute severe exacerbation of severe persistent asthma (disorder)** | ... |  | Y | **?****+****-****++****--** |
|  | · · · · Acute severe exacerbation of severe persistent asthma co-occurrent with allergic rhinitis (disorder) | ... |  | Y | **?****+****-** |
| Contract | · · **Acute exacerbation of asthma (disorder)** | ... |  | Y | **?****+****-****++****--** |
|  | · · · Acute exacerbation of asthma co-occurrent with allergic rhinitis (disorder) | ... |  | Y | **?****+****-** |
| Contract | · · · **Acute severe exacerbation of asthma (disorder)** | ... |  | Y | **?****+****-****++****--** |
|  | · · · · Acute severe exacerbation of asthma co-occurrent with allergic rhinitis (disorder) | ... |  | Y | **?****+****-** |
|  | · · · · Acute severe refractory exacerbation of asthma (disorder) | ... |  | Y | **?****+****-** |
|  | · · · · Life threatening acute exacerbation of asthma (disorder) | ... |  | Y | **?****+****-** |
|  | · · · · Acute severe exacerbation of allergic asthma (disorder) | ... |  | Y | **?****+****-** |
|  | · · · · · Acute severe exacerbation of immunoglobin E-mediated allergic asthma (disorder) | ... |  | Y | **?****+****-** |
|  | · · · · · Life threatening acute exacerbation of allergic asthma (disorder) | ... |  | Y | **?****+****-** |
|  | · · · · · Acute severe exacerbation of immunoglobin E-mediated allergic asthma (disorder) | ... |  | Y | **?****+****-** |
|  | · · · · · Life threatening acute exacerbation of allergic asthma (disorder) | ... |  | Y | **?****+****-** |
|  | · · · · · Acute severe exacerbation of immunoglobin E-mediated allergic asthma (disorder) | ... |  | Y | **?****+****-** |
|  | · · · · · Life threatening acute exacerbation of allergic asthma (disorder) | ... |  | Y | **?****+****-** |
|  | · · · · · Acute severe exacerbation of immunoglobin E-mediated allergic asthma (disorder) | ... |  | Y | **?****+****-** |
|  | · · · · Acute severe exacerbation of asthma co-occurrent with allergic rhinitis (disorder) | ... |  | Y | **?****+****-** |
|  | · · · Chronic obstructive asthma co-occurrent with acute exacerbation of asthma (disorder) | ... |  | Y | **?****+****-** |
|  | · · · Moderate acute exacerbation of asthma (disorder) | ... |  | Y | **?****+****-** |
|  | · · · Acute exacerbation of intrinsic asthma (disorder) | ... |  | Y | **?****+****-** |
|  | · · · · Acute severe exacerbation of intrinsic asthma (disorder) | ... |  | Y | **?****+****-** |
|  | · · · · · Life threatening acute exacerbation of intrinsic asthma (disorder) | ... |  | Y | **?****+****-** |
| Contract | · · **Exacerbation of allergic asthma (disorder)** | ... |  | Y | **?****+****-****++****--** |
|  | · · · Acute exacerbation of immunoglobulin E-mediated allergic asthma (disorder) | ... |  | Y | **?****+****-** |
|  | · · · Exacerbation of allergic asthma due to infection (disorder) | ... |  | Y | **?****+****-** |
|  | · · · Acute exacerbation of immunoglobulin E-mediated allergic asthma (disorder) | ... |  | Y | **?****+****-** |
|  | · Exercise-induced asthma (disorder) | ... |  | Y | **?****+****-** |
| Contract | · **Intermittent asthma (disorder)** | ... |  | Y | **?****+****-****++****--** |
|  | · · Intermittent asthma co-occurrent with allergic rhinitis (disorder) | ... |  | Y | **?****+****-** |
|  | · · Intermittent asthma uncontrolled (disorder) | ... |  | Y | **?****+****-** |
|  | · · Mild intermittent asthma (disorder) | ... |  | Y | **?****+****-** |
|  | · · Exacerbation of intermittent asthma (disorder) | ... |  | Y | **?****+****-** |
|  | · Late onset asthma (disorder) | ... |  | Y | **?****+****-** |
| Contract | · **Mild asthma (disorder)** | ... |  | Y | **?****+****-****++****--** |
| Contract | · · **Mild persistent asthma (disorder)** | ... |  | Y | **?****+****-****++****--** |
| Contract | · · · **Mild persistent asthma co-occurrent with allergic rhinitis (disorder)** | ... |  | Y | **?****+****-****++****--** |
|  | · · · · Acute severe exacerbation of mild persistent allergic asthma co-occurrent with allergic rhinitis (disorder) | ... |  | Y | **?****+****-** |
|  | · · · Mild persistent allergic asthma (disorder) | ... |  | Y | **?****+****-** |
| Contract | · · · **Exacerbation of mild persistent asthma (disorder)** | ... |  | Y | **?****+****-****++****--** |
|  | · · · · Acute exacerbation of mild persistent asthma (disorder) | ... |  | Y | **?****+****-** |
|  | · · · · · Acute severe exacerbation of mild persistent asthma (disorder) | ... |  | Y | **?****+****-** |
|  | · · · · · · Acute severe exacerbation of mild persistent allergic asthma (disorder) | ... |  | Y | **?****+****-** |
|  | · · · · · · Acute severe exacerbation of mild persistent allergic asthma co-occurrent with allergic rhinitis (disorder) | ... |  | Y | **?****+****-** |
|  | · · · · · · Acute severe exacerbation of mild persistent allergic asthma (disorder) | ... |  | Y | **?****+****-** |
|  | · · · · · · Acute severe exacerbation of mild persistent allergic asthma co-occurrent with allergic rhinitis (disorder) | ... |  | Y | **?****+****-** |
|  | · · · · · · Acute severe exacerbation of mild persistent allergic asthma (disorder) | ... |  | Y | **?****+****-** |
|  | · · · · · · Acute severe exacerbation of mild persistent allergic asthma co-occurrent with allergic rhinitis (disorder) | ... |  | Y | **?****+****-** |
|  | · · · · · · Acute severe exacerbation of mild persistent allergic asthma (disorder) | ... |  | Y | **?****+****-** |
|  | · · · · · · Acute severe exacerbation of mild persistent allergic asthma co-occurrent with allergic rhinitis (disorder) | ... |  | Y | **?****+****-** |
|  | · · · · · · Acute severe exacerbation of mild persistent allergic asthma (disorder) | ... |  | Y | **?****+****-** |
|  | · · · · · · Acute severe exacerbation of mild persistent allergic asthma co-occurrent with allergic rhinitis (disorder) | ... |  | Y | **?****+****-** |
|  | · · · · · · Acute severe exacerbation of mild persistent allergic asthma (disorder) | ... |  | Y | **?****+****-** |
|  | · · · · · · Acute severe exacerbation of mild persistent allergic asthma co-occurrent with allergic rhinitis (disorder) | ... |  | Y | **?****+****-** |
|  | · · · · · · Acute severe exacerbation of mild persistent allergic asthma (disorder) | ... |  | Y | **?****+****-** |
|  | · · · · · · Acute severe exacerbation of mild persistent allergic asthma co-occurrent with allergic rhinitis (disorder) | ... |  | Y | **?****+****-** |
|  | · · · Uncomplicated mild persistent asthma (disorder) | ... |  | Y | **?****+****-** |
|  | · · Mild intermittent asthma (disorder) | ... |  | Y | **?****+****-** |
|  | · Mixed asthma (disorder) | ... |  | Y | **?****+****-** |
| Contract | · **Moderate asthma (disorder)** | ... |  | Y | **?****+****-****++****--** |
| Contract | · · **Moderate persistent asthma (disorder)** | ... |  | Y | **?****+****-****++****--** |
| Contract | · · · **Moderate persistent asthma co-occurrent with allergic rhinitis (disorder)** | ... |  | Y | **?****+****-****++****--** |
|  | · · · · Acute severe exacerbation of moderate persistent asthma co-occurrent with allergic rhinitis (disorder) | ... |  | Y | **?****+****-** |
|  | · · · Moderate persistent allergic asthma (disorder) | ... |  | Y | **?****+****-** |
| Contract | · · · **Exacerbation of moderate persistent asthma (disorder)** | ... |  | Y | **?****+****-****++****--** |
|  | · · · · Acute exacerbation of moderate persistent asthma (disorder) | ... |  | Y | **?****+****-** |
|  | · · · · · Acute severe exacerbation of moderate persistent asthma (disorder) | ... |  | Y | **?****+****-** |
|  | · · · · · · Acute severe exacerbation of moderate persistent allergic asthma (disorder) | ... |  | Y | **?****+****-** |
|  | · · · · · · Acute severe exacerbation of moderate persistent asthma co-occurrent with allergic rhinitis (disorder) | ... |  | Y | **?****+****-** |
|  | · · · · · · Acute severe exacerbation of moderate persistent allergic asthma (disorder) | ... |  | Y | **?****+****-** |
|  | · · · · · · Acute severe exacerbation of moderate persistent asthma co-occurrent with allergic rhinitis (disorder) | ... |  | Y | **?****+****-** |
|  | · · · · · · Acute severe exacerbation of moderate persistent allergic asthma (disorder) | ... |  | Y | **?****+****-** |
|  | · · · · · · Acute severe exacerbation of moderate persistent asthma co-occurrent with allergic rhinitis (disorder) | ... |  | Y | **?****+****-** |
|  | · · · · · · Acute severe exacerbation of moderate persistent allergic asthma (disorder) | ... |  | Y | **?****+****-** |
|  | · · · · · · Acute severe exacerbation of moderate persistent asthma co-occurrent with allergic rhinitis (disorder) | ... |  | Y | **?****+****-** |
|  | · · · · · · Acute severe exacerbation of moderate persistent allergic asthma (disorder) | ... |  | Y | **?****+****-** |
|  | · · · · · · Acute severe exacerbation of moderate persistent asthma co-occurrent with allergic rhinitis (disorder) | ... |  | Y | **?****+****-** |
|  | · · · · · · Acute severe exacerbation of moderate persistent allergic asthma (disorder) | ... |  | Y | **?****+****-** |
|  | · · · · · · Acute severe exacerbation of moderate persistent asthma co-occurrent with allergic rhinitis (disorder) | ... |  | Y | **?****+****-** |
|  | · · · · · · Acute severe exacerbation of moderate persistent allergic asthma (disorder) | ... |  | Y | **?****+****-** |
|  | · · · · · · Acute severe exacerbation of moderate persistent asthma co-occurrent with allergic rhinitis (disorder) | ... |  | Y | **?****+****-** |
|  | · · · Uncomplicated moderate persistent asthma (disorder) | ... |  | Y | **?****+****-** |
| Contract | · **Non-allergic asthma (disorder)** | ... |  | Y | **?****+****-****++****--** |
| Contract | · · **Acute exacerbation of intrinsic asthma (disorder)** | ... |  | Y | **?****+****-****++****--** |
| Contract | · · · **Acute severe exacerbation of intrinsic asthma (disorder)** | ... |  | Y | **?****+****-****++****--** |
|  | · · · · Life threatening acute exacerbation of intrinsic asthma (disorder) | ... |  | Y | **?****+****-** |
|  | · · Uncomplicated non-allergic asthma (disorder) | ... |  | Y | **?****+****-** |
|  | · · Intrinsic asthma without status asthmaticus (disorder) | ... |  | Y | **?****+****-** |
|  | · Occasional asthma (disorder) | ... |  | Y | **?****+****-** |
| Contract | · **Seasonal asthma (disorder)** | ... |  | Y | **?****+****-****++****--** |
|  | · · Hay fever with asthma (disorder) | ... |  | Y | **?****+****-** |
| Contract | · **Severe asthma (disorder)** | ... |  | Y | **?****+****-****++****--** |
|  | · · Severe asthma with fungal sensitisation (disorder) | ... |  | Y | **?****+****-** |
| Contract | · · **Severe persistent asthma (disorder)** | ... |  | Y | **?****+****-****++****--** |
|  | · · · Severe controlled persistent asthma (disorder) | ... |  | Y | **?****+****-** |
|  | · · · Severe persistent asthma co-occurrent with allergic rhinitis (disorder) | ... |  | Y | **?****+****-** |
|  | · · · Severe uncontrolled persistent asthma (disorder) | ... |  | Y | **?****+****-** |
|  | · · · Severe persistent allergic asthma (disorder) | ... |  | Y | **?****+****-** |
| Contract | · · · **Exacerbation of severe persistent asthma (disorder)** | ... |  | Y | **?****+****-****++****--** |
|  | · · · · Acute severe exacerbation of severe persistent asthma (disorder) | ... |  | Y | **?****+****-** |
|  | · · · · · Acute severe exacerbation of severe persistent allergic asthma (disorder) | ... |  | Y | **?****+****-** |
|  | · · · · · Acute severe exacerbation of severe persistent asthma co-occurrent with allergic rhinitis (disorder) | ... |  | Y | **?****+****-** |
|  | · · · · · Acute severe exacerbation of severe persistent allergic asthma (disorder) | ... |  | Y | **?****+****-** |
|  | · · · · · Acute severe exacerbation of severe persistent asthma co-occurrent with allergic rhinitis (disorder) | ... |  | Y | **?****+****-** |
|  | · · · · · Acute severe exacerbation of severe persistent allergic asthma (disorder) | ... |  | Y | **?****+****-** |
|  | · · · · · Acute severe exacerbation of severe persistent asthma co-occurrent with allergic rhinitis (disorder) | ... |  | Y | **?****+****-** |
|  | · · · · · Acute severe exacerbation of severe persistent allergic asthma (disorder) | ... |  | Y | **?****+****-** |
|  | · · · · · Acute severe exacerbation of severe persistent asthma co-occurrent with allergic rhinitis (disorder) | ... |  | Y | **?****+****-** |
|  | · · · Uncomplicated severe persistent asthma (disorder) | ... |  | Y | **?****+****-** |
|  | · · Near fatal asthma (disorder) | ... |  | Y | **?****+****-** |
| Contract | · **Steroid dependent asthma (disorder)** | ... |  | Y | **?****+****-****++****--** |
|  | · · Oral steroid-dependent asthma (disorder) | ... |  | Y | **?****+****-** |
| Contract | · **Substance induced asthma (disorder)** | ... |  | Y | **?****+****-****++****--** |
| Contract | · · **Allergic bronchopulmonary mycosis (disorder)** | ... |  | Y | **?****+****-****++****--** |
|  | · · · Allergic bronchopulmonary aspergillosis (disorder) | ... |  | Y | **?****+****-** |
| Contract | · · **Chemical-induced asthma (disorder)** | ... |  | Y | **?****+****-****++****--** |
| Contract | · · · **Aspirin-induced asthma (disorder)** | ... |  | Y | **?****+****-****++****--** |
|  | · · · · Aspirin exacerbated respiratory disease (disorder) | ... |  | Y | **?****+****-** |
|  | · · · Isocyanate induced asthma (disorder) | ... |  | Y | **?****+****-** |
|  | · · · Sulfite-induced asthma (disorder) | ... |  | Y | **?****+****-** |
|  | · · · Allergic asthma caused by Dermatophagoides farinae (disorder) | ... |  | Y | **?****+****-** |
|  | · · · Allergic asthma caused by Dermatophagoides pteronyssinus (disorder) | ... |  | Y | **?****+****-** |
| Contract | · · **Drug-induced asthma (disorder)** | ... |  | Y | **?****+****-****++****--** |
| Contract | · · · **Aspirin-induced asthma (disorder)** | ... |  | Y | **?****+****-****++****--** |
|  | · · · · Aspirin exacerbated respiratory disease (disorder) | ... |  | Y | **?****+****-** |
| Contract | · · **Occupational asthma (disorder)** | ... |  | Y | **?****+****-****++****--** |
|  | · · · Bakers' asthma (disorder) | ... |  | Y | **?****+****-** |
| Contract | · · · **Byssinosis (disorder)** | ... |  | Y | **?****+****-****++****--** |
|  | · · · · Byssinosis grade 3 (disorder) | ... |  | Y | **?****+****-** |
|  | · · · · Flax-dressers' disease (disorder) | ... |  | Y | **?****+****-** |
|  | · · · Cheese-makers' asthma (disorder) | ... |  | Y | **?****+****-** |
|  | · · · Detergent asthma (disorder) | ... |  | Y | **?****+****-** |
|  | · · · Meat-wrappers' asthma (disorder) | ... |  | Y | **?****+****-** |
|  | · · · Millers' asthma (disorder) | ... |  | Y | **?****+****-** |
|  | · · · Platinum asthma (disorder) | ... |  | Y | **?****+****-** |
|  | · · · Printers' asthma (disorder) | ... |  | Y | **?****+****-** |
|  | · · · Tea-makers' asthma (disorder) | ... |  | Y | **?****+****-** |
|  | · · · Weavers' cough (disorder) | ... |  | Y | **?****+****-** |
|  | · · · Colophony asthma (disorder) | ... |  | Y | **?****+****-** |
|  | · · · Isocyanate induced asthma (disorder) | ... |  | Y | **?****+****-** |
|  | · · Asthma caused by wood dust (disorder) | ... |  | Y | **?****+****-** |
| Contract | · **Uncomplicated asthma (disorder)** | ... |  | Y | **?****+****-****++****--** |
|  | · · Uncomplicated mild persistent asthma (disorder) | ... |  | Y | **?****+****-** |
|  | · · Uncomplicated moderate persistent asthma (disorder) | ... |  | Y | **?****+****-** |
|  | · · Uncomplicated severe persistent asthma (disorder) | ... |  | Y | **?****+****-** |
|  | · · Uncomplicated allergic asthma (disorder) | ... |  | Y | **?****+****-** |
|  | · · Uncomplicated non-allergic asthma (disorder) | ... |  | Y | **?****+****-** |
|  | Asthma confirmed (situation) | ... |  | Y | **?****+****-** |
| Contract | **History of asthma (situation)** | ... |  | Y | **?****+****-****++****--** |
|  | · History of aspirin exacerbated respiratory disease (situation) | ... |  | Y | **?****+****-** |

·
