# Supplementary material for: Translating and evaluating historic phenotyping algorithms using SNOMED CT
Source: J Am Med Inform Assoc. 2022 Sep 9;30(2):222–32. doi: 10.1093/jamia/ocac158 (PMC9846670; doi:10.1093/jamia/ocac158)
Supplement: ocac158_Supplementary_Data [file ocac158_supplementary_data.zip › ocac158_Supplementary_Data/Supp_4_hf_ext.html]

Extended SNOMED CT codelist for heart failure

# Extended SNOMED CT codelist for heart failure

## Instructions

This HTML document presents a hierarchy of SNOMED CT concepts.
In SNOMED CT, each concept has a distinct meaning and can
be linked to more general terms (ancestors) and more specific terms (descendants). The buttons allow you to explore the codelist at
different levels of the hierarchy, and mark whether or not you agree
with the inclusion of individual concepts or concept hierarchies. When
you have finished your review, you can download your final selection as a .CSV file by clicking the **Export** button below.

### Key to buttons for each concept

- Expand Show descendants of this concept
- Contract Hide descendants of this concept
- **?** Mark as unchecked
- **+** Add a concept
- **-** Remove a concept
- **++** Add a concept and all descendants
- **--** Remove a concept and all descendants

## Reviewing tools

Show top-level concepts only Show all concepts

**Mark all concepts as "checked"** **Mark all concepts as "unchecked"** Show unchecked concepts only

**Export** to
 .csv

| Expand | SNOMED CT concept | Comment | Checked | Included |  |
| --- | --- | --- | --- | --- | --- |
|  | Cardiac ascites (disorder) | ... |  | Y | **?****+****-** |
|  | Cardiac edema (disorder) | ... |  | Y | **?****+****-** |
| Contract | **Heart failure (disorder)** | ... |  | Y | **?****+****-****++****--** |
| Contract | · **Acute heart failure (disorder)** | ... |  | Y | **?****+****-****++****--** |
| Contract | · · **Acute congestive heart failure (disorder)** | ... |  | Y | **?****+****-****++****--** |
|  | · · · Acute exacerbation of chronic congestive heart failure (disorder) | ... |  | Y | **?****+****-** |
|  | · · · Acute left-sided congestive heart failure (disorder) | ... |  | Y | **?****+****-** |
| Contract | · · **Acute cor pulmonale (disorder)** | ... |  | Y | **?****+****-****++****--** |
|  | · · · Acute cor pulmonale co-occurrent and due to saddle embolus of pulmonary artery (disorder) | ... |  | Y | **?****+****-** |
| Contract | · · **Acute diastolic heart failure (disorder)** | ... |  | Y | **?****+****-****++****--** |
| Contract | · · · **Acute combined systolic and diastolic heart failure (disorder)** | ... |  | Y | **?****+****-****++****--** |
|  | · · · · Acute on chronic combined systolic and diastolic heart failure (disorder) | ... |  | Y | **?****+****-** |
|  | · · · Acute on chronic diastolic heart failure (disorder) | ... |  | Y | **?****+****-** |
|  | · · Acute kidney injury due to circulatory failure (disorder) | ... |  | Y | **?****+****-** |
|  | · · Acute left-sided heart failure (disorder) | ... |  | Y | **?****+****-** |
|  | · · Acute right-sided heart failure (disorder) | ... |  | Y | **?****+****-** |
| Contract | · · **Acute systolic heart failure (disorder)** | ... |  | Y | **?****+****-****++****--** |
|  | · · · Acute on chronic systolic heart failure (disorder) | ... |  | Y | **?****+****-** |
|  | · Cardiac failure after obstetrical surgery AND/OR other procedure including delivery (disorder) | ... |  | Y | **?****+****-** |
| Contract | · **Cardiac insufficiency during AND/OR resulting from a procedure (disorder)** | ... |  | Y | **?****+****-****++****--** |
|  | · · Cardiac insufficiency due to prosthesis (disorder) | ... |  | Y | **?****+****-** |
| Contract | · · **Cardiac insufficiency following cardiac surgery (disorder)** | ... |  | Y | **?****+****-****++****--** |
|  | · · · Postvalvulotomy syndrome (disorder) | ... |  | Y | **?****+****-** |
| Contract | · **Cardiorenal syndrome (disorder)** | ... |  | Y | **?****+****-****++****--** |
|  | · · Acute kidney injury due to circulatory failure (disorder) | ... |  | Y | **?****+****-** |
|  | · Cardiorespiratory failure (disorder) | ... |  | Y | **?****+****-** |
| Contract | · **Chronic heart failure (disorder)** | ... |  | Y | **?****+****-****++****--** |
| Contract | · · **Chronic congestive heart failure (disorder)** | ... |  | Y | **?****+****-****++****--** |
|  | · · · Chronic left-sided congestive heart failure (disorder) | ... |  | Y | **?****+****-** |
| Contract | · · **Chronic diastolic heart failure (disorder)** | ... |  | Y | **?****+****-****++****--** |
|  | · · · Chronic combined systolic and diastolic heart failure (disorder) | ... |  | Y | **?****+****-** |
|  | · · Chronic left-sided heart failure (disorder) | ... |  | Y | **?****+****-** |
| Contract | · · **Chronic right-sided heart failure (disorder)** | ... |  | Y | **?****+****-****++****--** |
|  | · · · Chronic cor pulmonale (disorder) | ... |  | Y | **?****+****-** |
|  | · · Chronic systolic heart failure (disorder) | ... |  | Y | **?****+****-** |
|  | · · Decompensated chronic heart failure (disorder) | ... |  | Y | **?****+****-** |
|  | · Compensated cardiac failure (disorder) | ... |  | Y | **?****+****-** |
|  | · Congenital cardiac failure (disorder) | ... |  | Y | **?****+****-** |
| Contract | · **Congestive heart failure (disorder)** | ... |  | Y | **?****+****-****++****--** |
|  | · · Benign hypertensive heart disease with congestive cardiac failure (disorder) | ... |  | Y | **?****+****-** |
|  | · · Biventricular congestive heart failure (disorder) | ... |  | Y | **?****+****-** |
|  | · · Congestive heart failure as early postoperative complication (disorder) | ... |  | Y | **?****+****-** |
|  | · · Congestive heart failure due to cardiomyopathy (disorder) | ... |  | Y | **?****+****-** |
|  | · · Congestive heart failure due to left ventricular systolic dysfunction (disorder) | ... |  | Y | **?****+****-** |
|  | · · Congestive heart failure due to valvular disease (disorder) | ... |  | Y | **?****+****-** |
| Contract | · · **Congestive heart failure stage B (disorder)** | ... |  | Y | **?****+****-****++****--** |
|  | · · · Congestive heart failure stage B due to ischemic cardiomyopathy (disorder) | ... |  | Y | **?****+****-** |
| Contract | · · **Congestive heart failure stage C (disorder)** | ... |  | Y | **?****+****-****++****--** |
|  | · · · Congestive heart failure stage C due to Ischemic cardiomyopathy (disorder) | ... |  | Y | **?****+****-** |
|  | · · Congestive heart failure stage D (disorder) | ... |  | Y | **?****+****-** |
|  | · · Congestive heart failure with right heart failure (disorder) | ... |  | Y | **?****+****-** |
| Contract | · · **Congestive rheumatic heart failure (disorder)** | ... |  | Y | **?****+****-****++****--** |
|  | · · · Rheumatic left ventricular failure (disorder) | ... |  | Y | **?****+****-** |
|  | · · Exacerbation of congestive heart failure (disorder) | ... |  | Y | **?****+****-** |
| Contract | · · **Hypertensive heart and renal disease with (congestive) heart failure (disorder)** | ... |  | Y | **?****+****-****++****--** |
|  | · · · Hypertensive heart AND chronic kidney disease with congestive heart failure (disorder) | ... |  | Y | **?****+****-** |
|  | · · Hypertensive heart and renal disease with both (congestive) heart failure and renal failure (disorder) | ... |  | Y | **?****+****-** |
| Contract | · · **Hypertensive heart disease with congestive heart failure (disorder)** | ... |  | Y | **?****+****-****++****--** |
|  | · · · Malignant hypertensive heart disease with congestive heart failure (disorder) | ... |  | Y | **?****+****-** |
|  | · · Symptomatic congestive heart failure (disorder) | ... |  | Y | **?****+****-** |
|  | · · Acute congestive heart failure (disorder) | ... |  | Y | **?****+****-** |
| Contract | · · **Chronic congestive heart failure (disorder)** | ... |  | Y | **?****+****-****++****--** |
|  | · · · Acute exacerbation of chronic congestive heart failure (disorder) | ... |  | Y | **?****+****-** |
| Contract | · **Decompensated cardiac failure (disorder)** | ... |  | Y | **?****+****-****++****--** |
|  | · · Decompensated chronic heart failure (disorder) | ... |  | Y | **?****+****-** |
| Contract | · **Diastolic heart failure (disorder)** | ... |  | Y | **?****+****-****++****--** |
|  | · · Diastolic heart failure stage C (disorder) | ... |  | Y | **?****+****-** |
|  | · · Diastolic heart failure stage D (disorder) | ... |  | Y | **?****+****-** |
|  | · · Acute diastolic heart failure (disorder) | ... |  | Y | **?****+****-** |
| Contract | · · **Chronic diastolic heart failure (disorder)** | ... |  | Y | **?****+****-****++****--** |
|  | · · · Acute on chronic diastolic heart failure (disorder) | ... |  | Y | **?****+****-** |
| Contract | · **Fetal heart failure (disorder)** | ... |  | Y | **?****+****-****++****--** |
|  | · · Fetal heart failure due to extracardiac disease (disorder) | ... |  | Y | **?****+****-** |
|  | · · Fetal heart failure with myocardial hypertrophy (disorder) | ... |  | Y | **?****+****-** |
|  | · · Fetal heart failure with redistribution of cardiac output (disorder) | ... |  | Y | **?****+****-** |
|  | · Heart failure as a complication of care (disorder) | ... |  | Y | **?****+****-** |
|  | · Heart failure due to end stage congenital heart disease (disorder) | ... |  | Y | **?****+****-** |
|  | · Heart failure with normal ejection fraction (disorder) | ... |  | Y | **?****+****-** |
| Contract | · **Heart failure with reduced ejection fraction (disorder)** | ... |  | Y | **?****+****-****++****--** |
|  | · · Heart failure with reduced ejection fraction due to cardiomyopathy (disorder) | ... |  | Y | **?****+****-** |
|  | · · Heart failure with reduced ejection fraction due to coronary artery disease (disorder) | ... |  | Y | **?****+****-** |
|  | · · Heart failure with reduced ejection fraction due to heart valve disease (disorder) | ... |  | Y | **?****+****-** |
|  | · · Heart failure with reduced ejection fraction due to myocarditis (disorder) | ... |  | Y | **?****+****-** |
|  | · High output heart failure (disorder) | ... |  | Y | **?****+****-** |
| Contract | · **Hypertensive heart failure (disorder)** | ... |  | Y | **?****+****-****++****--** |
| Contract | · · **Hypertensive heart disease with congestive heart failure (disorder)** | ... |  | Y | **?****+****-****++****--** |
|  | · · · Malignant hypertensive heart disease with congestive heart failure (disorder) | ... |  | Y | **?****+****-** |
|  | · Induced termination of pregnancy complicated by cardiac failure (disorder) | ... |  | Y | **?****+****-** |
| Contract | · **Left heart failure (disorder)** | ... |  | Y | **?****+****-****++****--** |
|  | · · Acute left ventricular failure (disorder) | ... |  | Y | **?****+****-** |
|  | · · Cardiac asthma (disorder) | ... |  | Y | **?****+****-** |
|  | · · Rheumatic left ventricular failure (disorder) | ... |  | Y | **?****+****-** |
|  | · · Sepsis-associated left ventricular failure (disorder) | ... |  | Y | **?****+****-** |
| Contract | · · **Acute left-sided heart failure (disorder)** | ... |  | Y | **?****+****-****++****--** |
|  | · · · Acute left-sided congestive heart failure (disorder) | ... |  | Y | **?****+****-** |
| Contract | · · **Chronic left-sided heart failure (disorder)** | ... |  | Y | **?****+****-****++****--** |
|  | · · · Chronic left-sided congestive heart failure (disorder) | ... |  | Y | **?****+****-** |
| Contract | · **Low output heart failure (disorder)** | ... |  | Y | **?****+****-****++****--** |
|  | · · Low cardiac output syndrome (disorder) | ... |  | Y | **?****+****-** |
| Contract | · **Neonatal cardiac failure (disorder)** | ... |  | Y | **?****+****-****++****--** |
|  | · · Neonatal cardiac failure due to decreased left ventricular output (disorder) | ... |  | Y | **?****+****-** |
|  | · · Neonatal cardiac failure due to pulmonary overperfusion (disorder) | ... |  | Y | **?****+****-** |
|  | · Refractory heart failure (disorder) | ... |  | Y | **?****+****-** |
| Contract | · **Right heart failure (disorder)** | ... |  | Y | **?****+****-****++****--** |
|  | · · Acute right-sided congestive heart failure (disorder) | ... |  | Y | **?****+****-** |
|  | · · Bernheim's syndrome (disorder) | ... |  | Y | **?****+****-** |
| Contract | · · **Cor pulmonale (disorder)** | ... |  | Y | **?****+****-****++****--** |
|  | · · · Ayerza's syndrome (disorder) | ... |  | Y | **?****+****-** |
| Contract | · · · **Acute cor pulmonale (disorder)** | ... |  | Y | **?****+****-****++****--** |
|  | · · · · Acute cor pulmonale co-occurrent and due to saddle embolus of pulmonary artery (disorder) | ... |  | Y | **?****+****-** |
|  | · · Right heart failure due to pulmonary hypertension (disorder) | ... |  | Y | **?****+****-** |
|  | · · Right heart failure secondary to left heart failure (disorder) | ... |  | Y | **?****+****-** |
| Contract | · · **Chronic right-sided heart failure (disorder)** | ... |  | Y | **?****+****-****++****--** |
|  | · · · Chronic cor pulmonale (disorder) | ... |  | Y | **?****+****-** |
|  | · · · Chronic right-sided congestive heart failure (disorder) | ... |  | Y | **?****+****-** |
|  | · · · Chronic cor pulmonale (disorder) | ... |  | Y | **?****+****-** |
|  | · · · Chronic right-sided congestive heart failure (disorder) | ... |  | Y | **?****+****-** |
|  | · · Biventricular congestive heart failure (disorder) | ... |  | Y | **?****+****-** |
|  | · · Congestive heart failure with right heart failure (disorder) | ... |  | Y | **?****+****-** |
|  | · · Biventricular congestive heart failure (disorder) | ... |  | Y | **?****+****-** |
| Contract | · **Right ventricular failure (disorder)** | ... |  | Y | **?****+****-****++****--** |
|  | · · Chronic right-sided congestive heart failure (disorder) | ... |  | Y | **?****+****-** |
|  | · · Sepsis-associated right ventricular failure (disorder) | ... |  | Y | **?****+****-** |
| Contract | · · **Acute right-sided heart failure (disorder)** | ... |  | Y | **?****+****-****++****--** |
|  | · · · Acute right-sided congestive heart failure (disorder) | ... |  | Y | **?****+****-** |
|  | · · Bernheim's syndrome (disorder) | ... |  | Y | **?****+****-** |
| Contract | · **Systolic heart failure (disorder)** | ... |  | Y | **?****+****-****++****--** |
| Contract | · · **Systolic heart failure stage C (disorder)** | ... |  | Y | **?****+****-****++****--** |
|  | · · · Systolic heart failure stage C due to ischemic cardiomyopathy (disorder) | ... |  | Y | **?****+****-** |
|  | · · Systolic heart failure stage D (disorder) | ... |  | Y | **?****+****-** |
| Contract | · · **Acute systolic heart failure (disorder)** | ... |  | Y | **?****+****-****++****--** |
|  | · · · Acute combined systolic and diastolic heart failure (disorder) | ... |  | Y | **?****+****-** |
| Contract | · · **Chronic systolic heart failure (disorder)** | ... |  | Y | **?****+****-****++****--** |
|  | · · · Acute on chronic systolic heart failure (disorder) | ... |  | Y | **?****+****-** |
|  | · · · Chronic combined systolic and diastolic heart failure (disorder) | ... |  | Y | **?****+****-** |
|  | · · · Acute on chronic systolic heart failure (disorder) | ... |  | Y | **?****+****-** |
|  | · · · · Acute on chronic combined systolic and diastolic heart failure (disorder) | ... |  | Y | **?****+****-** |
|  | Heart failure confirmed (situation) | ... |  | Y | **?****+****-** |
| Contract | **History of heart failure (situation)** | ... |  | Y | **?****+****-****++****--** |
|  | · History of heart failure in last year (situation) | ... |  | Y | **?****+****-** |
|  | Pleural effusion due to congestive heart failure (disorder) | ... |  | Y | **?****+****-** |

·
